# Supplementary material for: Nanoencapsulation of buriti oil (Mauritia flexuosa L.f.) in porcine gelatin enhances the antioxidant potential and improves the effect on the antibiotic activity modulation
Source: PLoS One. 2022 Mar 18;17(3):e0265649. doi: 10.1371/journal.pone.0265649 (PMC8932573; doi:10.1371/journal.pone.0265649)
Supplement: Attachment — Submitted filename: PONE-D-21-38955_reviewer.pdf [file pone.0265649.s002.pdf]

PLOS ONE

Nanoencapsulation of buriti oil in porcine gelatin enhances the antioxidant potential and improves the effect on the antibiotic activity modulation

--Manuscript Draft--

**Manuscript Number:**	PONE-D-21-38955
**Article Type:**	Research Article
**Full Title:**	Nanoencapsulation of buriti oil in porcine gelatin enhances the antioxidant potential and improves the effect on the antibiotic activity modulation
**Short Title:**	Nanoencapsulation of buriti oil in porcine gelatin
**Corresponding Author:**	Cristiane Fernandes de Assis, PHD<br>Universidade Federal do Rio Grande<br>Natal, Rio Grande do Norte BRAZIL
**Keywords:**	Cytotoxicity; Total Antioxidant Capacity, ABTS, Gentamicin; Norfloxacin
**Abstract:**	The present study evaluated the cytotoxicity, antioxidant potential, and antimicrobial effect on the antibiotic activity modulation of gelatin nanoparticles containing buriti oil (OPG). The cytotoxicity analysis was performed on Chinese Hamster Ovary Cells (CHO) using a MTT test. The antioxidant potential of buriti oil and OPG was determined by total antioxidant capacity, reducing power, and the ABTS test. The modulating antimicrobial activity was evaluated by determining the minimum inhibitory concentration (MIC) using Escherichia coli, Pseudomonas aeruginosa, Staphylococcus aureus, gentamicin and norflaxacillin. The nanoformulation did not show a cytotoxic effect on CHO cells. OPG presented higher antioxidant potential than free buriti oil ($p<0.05$). The combination of antibiotics with free buriti oil and OPG was more efficient in inhibiting E. coli and P. aeruginosa than isolated antibiotics ($p<0.05$). Regarding the inhibition of S. aureus , OPG in combination with norfloxacillin reduced MIC by 50%. Nanoencapsulation was a viable alternative to enhance functionality, adding commercial value to buriti oil.
**Order of Authors:**	Neyna de Santos Morais
	Thaís Souza Passos
	Gabriela Rocha Ramos
	Victória Azevedo Freire Ferreira
	Suzana Margarida Gomes Moreira
	Gildácio Pereira Chaves Filho
	Ana Paula Gomes Barreto
	Pedro Ivo Palacio Leite
	Ray Silva de Almeida
	Cícera Laura Roque Paulo
	Rafael Fernandes
	Sara Sayonara da Cruz Nascimento
	Sebastião Ânderson Dantas da Silva
	Francisco Canindé Sousa Júnior
	Cristiane Fernandes de Assis, PHD
**Additional Information:**	
**Question**	**Response**
**Financial Disclosure**	This study was partly financed by the<br>Coordenação de Aperfeiçoamento de Pessoal de Nível Superior - Brasil (CAPES) -

Enter a financial disclosure statement that describes the sources of funding for the work included in this submission. Review the submission guidelines for detailed requirements. View published research articles from *PLOS ONE* for specific examples.<br><br>This statement is required for submission and **will appear in the published article** if the submission is accepted. Please make sure it is accurate.<br><br>**Unfunded studies**<br>Enter: *The author(s) received no specific funding for this work.*<br><br>**Funded studies**<br>Enter a statement with the following details:<br>• Initials of the authors who received each award<br>• Grant numbers awarded to each author<br>• The full name of each funder<br>• URL of each funder website<br>• Did the sponsors or funders play any role in the study design, data collection and analysis, decision to publish, or preparation of the manuscript?<br>• **NO** - Include this sentence at the end of your statement: *The funders had no role in study design, data collection and analysis, decision to publish, or preparation of the manuscript.*<br>• **YES** - Specify the role(s) played.<br><br>* typeset	Finance Code 001.
**Competing Interests**<br><br>Use the instructions below to enter a competing interest statement for this submission. On behalf of all authors, disclose any competing interests that could be perceived to bias this work—acknowledging all financial support and any other relevant financial or non-financial competing interests.<br><br>This statement is required for submission and **will appear in the published article** if the submission is accepted. Please make	The authors have declared that no competing interests exist.

sure it is accurate and that any funding sources listed in your Funding Information later in the submission form are also declared in your Financial Disclosure statement.<br><br>View published research articles from *PLOS ONE* for specific examples.<br><br>**NO authors have competing interests**<br><br>Enter: *The authors have declared that no competing interests exist.*<br><br>**Authors with competing interests**<br><br>Enter competing interest details beginning with this statement:<br><br>*I have read the journal's policy and the authors of this manuscript have the following competing interests: [insert competing interests here]*<br><br>* typeset	
**Ethics Statement**<br><br>Enter an ethics statement for this submission. This statement is required if the study involved:<br><br>• Human participants<br>• Human specimens or tissue<br>• Vertebrate animals or cephalopods<br>• Vertebrate embryos or tissues<br>• Field research<br><br>Write "N/A" if the submission does not require an ethics statement.<br><br>General guidance is provided below. Consult the submission guidelines for detailed instructions. **Make sure that all information entered here is included in the Methods section of the manuscript.**	N/A

Powered by Editorial Manager® and ProduXion Manager® from Aries Systems Corporation

**Format for specific study types**<br><br>**Human Subject Research (involving human participants and/or tissue)**<br>• Give the name of the institutional review board or ethics committee that approved the study<br>• Include the approval number and/or a statement indicating approval of this research<br>• Indicate the form of consent obtained (written/oral) or the reason that consent was not obtained (e.g. the data were analyzed anonymously)<br><br>**Animal Research (involving vertebrate animals, embryos or tissues)**<br>• Provide the name of the Institutional Animal Care and Use Committee (IACUC) or other relevant ethics board that reviewed the study protocol, and indicate whether they approved this research or granted a formal waiver of ethical approval<br>• Include an approval number if one was obtained<br>• If the study involved *non-human primates*, add *additional details* about animal welfare and steps taken to ameliorate suffering<br>• If anesthesia, euthanasia, or any kind of animal sacrifice is part of the study, include briefly which substances and/or methods were applied<br><br>**Field Research**<br><br>Include the following details if this study involves the collection of plant, animal, or other materials from a natural setting:<br>• Field permit number<br>• Name of the institution or relevant body that granted permission	
**Data Availability**<br><br>Authors are required to make all data underlying the findings described fully available, without restriction, and from the time of publication. PLOS allows rare exceptions to address legal and ethical concerns. See the PLOS Data Policy and FAQ for detailed information.	Yes - all data are fully available without restriction

A Data Availability Statement describing where the data can be found is required at submission. Your answers to this question constitute the Data Availability Statement and **will be published in the article**, if accepted.<br><br>**Important:** Stating 'data available on request from the author' is not sufficient. If your data are only available upon request, select 'No' for the first question and explain your exceptional situation in the text box.<br><br>Do the authors confirm that all data underlying the findings described in their manuscript are fully available without restriction?	
**Describe where the data may be found in full sentences. If you are copying our sample text, replace any instances of XXX with the appropriate details.**<br><br>• If the data are **held or will be held in a public repository**, include URLs, accession numbers or DOIs. If this information will only be available after acceptance, indicate this by ticking the box below. For example: *All XXX files are available from the XXX database (accession number(s) XXX, XXX.*).<br>• If the data are all contained **within the manuscript and/or Supporting Information files**, enter the following: *All relevant data are within the manuscript and its Supporting Information files.*<br>• If neither of these applies but you are able to provide **details of access elsewhere**, with or without limitations, please do so. For example:<br><br>*Data cannot be shared publicly because of [XXX]. Data are available from the XXX Institutional Data Access / Ethics Committee (contact via XXX) for researchers who meet the criteria for access to confidential data.*<br><br>*The data underlying the results presented in the study are available from (include the name of the third party*	All relevant data are within the manuscript and its Supporting Information files.

Powered by Editorial Manager® and ProduXion Manager® from Aries Systems Corporation

and contact information or URL).<br>• This text is appropriate if the data are owned by a third party and authors do not have permission to share the data.<br><br>* typeset	
Additional data availability information:	Tick here if the URLs/accession numbers/DOIs will be available only after acceptance of the manuscript for publication so that we can ensure their inclusion before publication.

Powered by Editorial Manager® and ProduXion Manager® from Aries Systems Corporation

Manuscript

Click here to access/download;Manuscript;Morais, et al., 2021.docx

Nanoencapsulation of buriti oil [illegible] porcine gelatin enhances the antioxidant potential and improves the effect on the antibiotic activity modulation

[1]Neyna de Santos Morais, [2]Thaís de Souza Passos, [3]Gabriela Rocha Ramos, [3]Victoria Azevedo Freire Ferreira, [4]Suzana Margarida Gomes Moreira, [4]Gildácio Pereira Chaves Filho, [3]Ana Paula Gomes Barreto, [5]Pedro Ivo Palacio Leite, [5]Ray Silva de Almeida, [5]Cícera Laura Roque Paula, [6]Rafael Fernandes, [1]Sebastião Ânderson Dantas da Silva, [7]Sara Sayonara da Cruz Nascimento, [1,3]Francisco Canindé de Sousa Júnioar,[1,3]*Cristiane Fernandes de Assis

[1]Nutrition Postgraduate Program, Center for Health Sciences, Federal University of Rio Grande do Norte, Natal, RN, Brazil.

[2]Department of Nutrition, Center for Health Sciences, Federal University of Rio Grande do Norte, Natal, RN, Brazil.

[3]Department of Pharmacy, Center for Health Sciences, Federal University of Rio Grande do Norte, Natal, RN, Brazil.

[4]Department of Cellular and Molecular Biology, Biosciences Center, Federal University of Rio Grande do Norte, Natal, RN, Brazil.

[5]Department of Chemical Biology, Regional University of Cariri, Crato, CE, Brasil.

[6]Chemical Intitute, Federal University of Rio Grande do Norte, Natal, RN, Brazil.

[7]Postgraduate Program in Biotechnology - RENORBIO, Biosciences Center, Federal University of Rio Grande do Norte, Natal, RN, Brazil.

*Corresponding author: cristianeassis@hotmail.com

Abstract

The present study evaluated the cytotoxicity, antioxidant potential, and antimicrobial effect on the antibiotic activity modulation of gelatin nanoparticles containing buriti oil (OPG). The cytotoxicity analysis was performed on Chinese Hamster Ovary Cells (CHO) using a MTT test. The antioxidant potential of buriti oil and OPG was determined by total antioxidant capacity, reducing power, and the ABTS test. The modulating antimicrobial activity was evaluated by determining the minimum inhibitory concentration (MIC) using *Escherichia coli, Pseudomonas aeruginosa, Staphylococcus aureus,* gentamicin and norflaxacillin. The nanoformulation did not show a cytotoxic effect on CHO cells. OPG presented higher antioxi[illegible]t potential than free buriti oil ($p<0.05$). The combination of antibiotics with free buriti oil and OPG was more efficient in inhibiting *E. coli* and *P. aeruginosa* than isolated antibiotics ($p<0.05$). Regarding the inhibition of *S. aureus*, OPG in combination with norfloxacillin reduced MIC by 50%. Nanoencapsulation was a viable alternative to enhance functionality, adding commercial value to buriti oil.

Introduction

Mauritia palm or buriti (*Mauritia flexuosa* L.f.) is an abundant palm tree in South America, predominant in extensive areas of Brazil and native to areas of the Amazon Forest and Cerrado biome(1). Buriti fruit is considered [illegible]unctional food due to its high levels of carotenoids. The pulp is regarded one of the primary sources of provitamin A found in Brazilian flora diversity, in particular β carotene(2), in addition to containing minerals (calcium, zinc, sodium), fibers, proteins, and unsaturated fatty acids(3)

Buriti oil can be obtained from the fruit by extracting the pulp and/or pulp and peel by conventional methods (artisanal, mechanical, and chemical extraction)(4–6), capable of influencing the product characteristics. This oil is of great interest in the cosmetic and food product industries because it has a wide and varied application due to its chemical composition(7).

Buriti oil contains tocopherols which compose vitamin E, considered an important antioxidant capable of interrupting the action of free radicals and protecting the cytoplasmic membranes from oxidation, reducing pre-cancerous lesions(8–1[illegible] In addition, it also has unsaturated fatty acids (mono and polyunsaturate[illegible] which are associated with reduced levels of triglycerides and total cholesterol, consequently constituting a protective factor for cardiovascular diseases(11), and a protective effect on platelet activation and thrombosis(12).

It is well-known that buriti oil has numerous biological properties. However, the bioactive compounds present are susceptible to degradation as they are sensitive to food processing and storage factors, such as temperature, pH, presence of light, and oxygen(13,14[illegible]he oil's oxidation process causes unpleasant tastes, loses nutrients, and produces many toxic compound[illegible]at cause mutation, aging, stroke, emphysema, heart disease, and cancer(15). Another technological obstacle associated with the use of buriti oil in foods is associated with its lipophilic nature, which makes it challenging to apply in products with aqueous matrices, limiting the development of new products(16).

In this context, nanoencapsulation is a strategy that can be used to protect substances against environmental factors (heat, light, oxygen, humidity), improve stability, acceptability, and handling conditions, restrict contact with other components and control the release and delivery of bioactives to the target site. In addition, reducing the size to the nanometric scale (< 100 nm) facilitates dispersion of lipophilic substances in an aqueous matrix due to the increase in the contact surface. It can also preserve or enhance the biological properties of bioactive compounds(15–18).

Encapsulated vegetable oils hold promise in food products because they can improve fatty acid profiles and extend shelf li[illegible]ue to their numerous functionalities, such as antioxidant and antimicrobial activity, they become a potential option for formulating functional foods by increasing the protection of bioactive compounds against oxidation(19).

Considering the properties present in vegetable oils, especially buriti oil, and the current needs of the food and pharmaceutical industry, our research group has been developing studies with nanoencapsulated vegetable oils(16,18). The results obtained from the nanoencapsulation

of buriti oil(16) indicate the need for further studies to understand the effects promoted by nanoencapsulation on the functionality of buriti oil, and evaluate the toxicity of the nanoformulation. Therefore, this study aimed to assess the cytotoxicity, antioxidant potential, and modulating action on antibiotics of nanoparticles based on porcine gelatin containing buriti oil (OPG).

Materials and Methods

Materials

Buriti oil from the *Mauritia flexuosa* species was supplied by the Plantus® S.A. cosmetics company (Nísia Floresta, in the Rio Grande do Norte, Brazil) with an IBD certificate (Agricultural and Food Inspections and Certification). The samples were transported under protection from light and refrigerated to the Bromatology Laboratory of the Department of Pharmacy at the Federal University of Rio Grande do Norte (UFRN), where they were stored at 4 ºC.

Porcine gelatin (Type A), the surfactant Tween 20, 2,2’-azinobis (3-ethylbenzothiazoline-6-sulfonic acid) (ABTS), and resazurin obtained from Sigma-Aldrich®.

Microorganisms

Multiresistant strains of *Staphylococcus aureus* 10, *Pseudomonas aeruginosa* 24, and *Escherichia coli* 06 were supplied by the Laboratory of Microbiology and Molecular Biology of the Regional University of Cariri - URCA (Crato - CE, Brazil). The Chinese hamster ovary cell line (CHO-K1[illegible]s) was purchased from ATCC® CCL-61™ (American Type Culture Collection), and Dulbecco’s Modified Eagle Culture Medium (DMEM; Gibco, Gaithersbug, MD, USA) was used, while 3-4,5-Dimethyl-thiazol-2-yl-2,5-diphenyltetrazolium bromide (MTT) was purchased from Invitrogen (MTT; Invitrogen, Oregon, USA).

Methods

Obtaining the nanoformulation containing buriti oil

The nanoformulation based on porcine gelatine containing buriti oil (OPG) was obtained through the technique of O/W emulsification followed by dispersion of a solution containing encapsulating agent in the obtained emulsion based on Medeiros et al.(20) with modifications proposed by Castro et al.(16). Porcine gelatin (Sigma®) and Tween 20 (Sigma®) were used as encapsulating agent and surfactant. After the encapsulation process, the resulting emulsion was subjected to drying by lyophilization (LioTop L101) at -57ºC and pressure of 43 μHg for further characterization, evaluation of cytotoxicity, antioxidant potential, and effect on the modulation of antibiotic activity.

Characterization of the obtained nanoparticles

The new batch of OPG obtained for this study was characterized to determine its morphology, particle diameter, and chemical interactions by Scanning Electron Microscopy, Dynamic Light Scattering, and Fourier Transform Infrared Spectroscopy, respectively, according to Castro et al.(16). Furthermore, it was also evaluated concerning the distribution of charges on the surface of the particles and in terms of thermal resistance by Zeta Potential, Thermogravimetry, and Differential Thermal Analysis, respectively.

Scanning electron microscopy (SEM)

OPG was suspended in acetone, and the dispersion was dropped onto silicon plates attached to stubs using carbon tape. It was subsequently analyzed at different magnifications in high vacuum at 2-3 kV and without metallization using a FEG-SEM ZEISS microscope (AURIGA).

Dynamic light scattering (DLS)

The nanoformulation was subjected to crosslinking with glutaraldehyde to measure the diameter of the particles, according to Castro et al.(16), to promote particle deagglomeration. It was subsequently dispersed using an ultrasonic bath in DMSO (1 mg/mL). Measurements were performed in triplicate (60 s each) using only 2 mL of suspension. Data were analyzed using the NANO-flex Control 0.9.7 software program. The entire experiment was carried out in triplicate. The data obtained referring to the mean and standard deviation were plotted in the Origin® 8 program to obtain the histogram with the particle size distribution.

Fourier transform infrared spectroscopy (FTIR)

Buriti oil, porcine gelatin, Tween 20, and OPG were homogenized separately with potassium bromide (KBr). Then they were macerated and pressed to obtain pellets. The spectra were recorded in transmittance with the mid-infrared region (400 to 4000 cm^{-1}). A Shimadzu spectrometer (FTIR-8400S, IRAFFINITY-1 series, IR SOLUTION version 1.60 software) was used with a scan number of 32 and resolution of 4 cm^{-1}.

Zeta Potential

The Zeta Potential measurements of the OPG nanoformulation were determined using a STABINO II Particle charge Titration device (Colloid Metrix). First, 10 mg of the sample was diluted in 10 mL of ultrapure water (≥ 18 MΩ cm-1) and then transferred to the cylindrical Teflon cell. The zeta potential measurement with pH variation (similar to a titration) consisted of individually adding aliquots (10 μL) of a strong acid (HCl - 0.1 N) or a strong base (NaOH – 0.025 N).

Thermogravimetry (tg) and differential thermal analysis (DTA)

We employed thermoanalytical analyzes to evaluate the buriti oil, Tween 20, porcine gelatin, and OPG nanoformulation. Thus, from 6 to 7 mg of each material were evaluated in a Shimadzu® DTG-60 thermal analyzer with a heating rate of 10 °C/min^{-1} in the temperature range of 30°C to 800°C in a nitrogen atmosphere with a flow of 50 mL/min^{-1}.

It is noteworthy that the variations in the residual mass of the materials concerning the variation in time and temperature were provided by the equipment. The Origin Pro Graphing & Analysis version 9.8.0.200 software program from OriginLab® was used to build the TG and DTA graphs.

Incorporation efficiency (%)

The incorporation efficiency was determined based on El-Messery et al.(21) with modifications. 15 mL of hexane in 1.5 g of the nanoformulation were added under agitation in a TE-421 rotary incubator (Tecnal®) for 2 min, and then the mixture was filtered on Whatman No.1 filter paper.

Next, the content retained on the filter paper was washed twice with 20 [illegible] of hexane. The liquid phase was collected, and the hexane was removed in an incubator at 60°C for 48 h. The remaining extract represents the unencapsulated oil present on the surface of the encapsulates. The amount of nanoencapsulated oil was obtained through the difference between the initial amount of oil used to promote the nanoencapsulation process and the free oil present on the surface of the nanoparticles after the hexane evaporates, as described below (Equation 1). The average amount of OPG obtained from the triplicate produced and measured with the aid of an analytical balance was considered (Edutec, EEQ9003F-B).

$$EO\ (g) = OUE - RO$$

EO: Quantity of encapsulated oil; OUE: Weight of oil used in encapsulation; RO: Weight of remaining oil.

The encapsulation incorporation was calculated using Equation 2, described below according to El-Messery et al.(21), considering the average amount of OPG obtained from the triplicate produced and measured using an analytical scale (Edutec, EEQ9003F-B).

$$EI\ (\%) = [\frac{EO}{IO}]x100$$

EI: Encapsulation incorporation; EO: Amount of encapsulated oil; IO: Initial oil quantity

Cytotoxicity evaluation

MTT assay

Chinese hamster ovary (CHO) cells were seeded in 96-well plates at a density of $2x10^3$ cells/m[illegible]d cultured in α-MEM medium supplemented with 10% FCS (fetal bovine serum), 1% antibiotic and antimycotic solution, and 1% glutamine (basal medium).

At the end of 24 hours, the medium was changed with the medium containing the buriti oil and OPG samples at concentrations 50, 100, and 500 µg/mL, and cytotoxicity was evaluated after 24 h, 48 h, and 72 [illegible]rs. Cells maintained in the basal medium were used as a negative control. At the end of each time, 1 mg.mL^{-1} of 3-(4,5-dimethylthiazol-2-yl)-2,5-diphenyltetrazolium bromide (MTT) solubilized in PBS buffer was added, the supernatant was aspirated after incubation at 37°C C for 4 hours, and the formazan crystals solubilized with 200 µl of DMSO. Absorbance was measured at 570 nm in a plate reader (BioTek, µQuant model), and cell viability was calculated according to Equation 3 described below.

$$MTT\ reduction(\%) = \frac{Abs\ treatment}{Abs\ negative\ control}\ x\ 100$$

Antioxidant activity

Total antioxidant capacity (TAC)

The method proposed by Prieto et al(22). was used to determine TAC. In each test tube, 100 µL of 40 mM sulfuric acid-ammonium molybdate, 280 mM sodium phosphate, and 100 µL of the sample solutions were added at a concentration of 2 mg/mL and then 700 µL of distilled water in the test tubes.

Then, the tubes were shaken and incubated in a water bath (QUIMIS, Mod. Q334M-28) at 90ºC for 90 minutes to subsequently take the absorbance reading by spectrophotometry (Biospectrum, Mod. SP-220) at 695 nm. The experiment was carried out in triplicate using solutions containing the encapsulating agents as a control. Antioxidant activity was expressed in milligrams of ascorbic acid per gram of sample (mg AA/g of sample). The standard curve was constructed using different ascorbic acid concentrations (25 -250 mg/g).

Reducing power test

The reducing power of the samples was quantified as described by Wang et al.(23). First, 4 mL of a reaction mixture containing different buriti oil sample (1-50 mg/mL) and OPG (3-20 mg/mL) concentrations in phosphate buffer (0.2 M, pH 6.6) were incubated with potassium ferrocyanide (1% m/v) at 50 ºC for 20 minutes. The reaction was terminated by a TCA solution (10% m/v). The solution was then mixed with distilled water and iron chloride (0.1% m/v), and the absorbance was measured at 700 nm. The control was carried out with the encapsulating agent. The result was expressed in the reduction of Fe^{+3} to Fe^{+2} (%).

Antioxidant activity by 2,2'-azinobis-3-ethylbenzothiazoline-6-sulfonic acid (ABTS•)

The ability to scavenge the 2,2'-azinobis-3-ethylbenzothiazoline-6-sulfonic acid (ABTS•) radical cation was determined by adapting the methodology described by Rufino et al.(24). The ABTS• solution was prepared by adding ABTS• (7 mM) and potassium persulfate (2.45 mM) and incubated at room temperature in the dark for 16 h. Next, 1 mL of this solution was diluted in 50 mL in a volumetric flask containing DMSO to obtain an absorbance of 0.8[112] at a wavelength of 734 nm. Finally, 200 µL of this ABTS• radical solution was carefully added to the microplate well together with 40 µL of the evaluated samples.

The control was prepared to contain 200 µL of ABTS and 40 µL of DMSO. The absorbance reading at 734 nm was performed in a microplate reader (Loccus – Model LMR FLEX). After reading the absorbances, the antioxidant activity (AA) percentage was estimated using Equation 4 described below.

$$ABTS\ radical\ inhibition = 100\ X\ \frac{Abs\ control - Abs\ sample}{Abs\ control}$$

The linear regression curve was determined to calculate the concentration required for the samples (crude oil and OPG) to inhibit 50% of the ABTS radical (IC 50).

Determination of the modulating antimicrobial activity of antibiotics

The antimicrobial activity test was performed by determining the Minimum Inhibitory Concentration (MIC) of antibiotics (norfloxacin and gentamycin) and modulating activity using buriti oil and OPG (dilution of in DMSO, in the concentration of 1024 µg/mL).

The bacteria used were multiresistant *Pseudomonas auriginosa* 24, *Staphylococcus aureus* 10, and *Escherichia coli* [illegible] strains collected in exams provided by the Regional University of Cariri (Juazeiro do Norte – CE). The origin and resistance profile of these strains were described by Bezerra et al.(25).

Bacteria cultures of 10^7 CFU/mL kept in agar were subcultured in brain-heart broth (Brain Heart Infusion - BHI) at 37ºC for 24 h. Then, the distribution medium was serially prepared at 1:10, and 100 µL were added into Eppendorfs of BHI medium in 900 µL of inoculum. This distribution medium was transferred to the microplate, 100 µL in each well. Norfloxacin and gentamycin antibiotics were added to the test, and their inhibitory effects were investigated in their combinations with buriti oil and OPG. Both concentrations of antibiotics, crude buriti oil and OPG, were fixed at 1024 µg/mL with a dilution of 100 µL in octuplicate. Controls were included in the trials, namely the medium and the inoculum, the porcine gelatin, and the Triton X-100. The filled plates were incubated at 35 (±2)ºC for 24 hours(26).

Next, an indicator solution of sodium resazurin (Sigma-Aldrich, USA) was prepared in sterile distilled water at a concentration of 0.01% (m/v) to evidence the MIC of the samples. After incubation, 20 µL of the indicator solution was added to each well and the plates underwent an

incubation period of 1 hour at room temperature. A change from blue to pink color due to the reduction of resazurin indicated bacterial growth(26). MIC was defined as the lowest concentration capable of inhibiting microbial growth, as evidenced by the unaltered blue color.

Statistical analysis

Statistical analysis was performed using Graph Pad Prism version 5.0 software. Results were expressed as mean and standard deviation. First, the data obtained for the analyzes related to antioxidant potential and antimicrobial activity and modulation of antibiotic action were evaluated for normality using the Shapiro-Wilk test. Data were evaluated using the Student's t-test to determine the antioxidant potential through CAT and ABTS tests and assess antimicrobial activity and cytotoxicity. An analysis of variance and Tukey's post-test were used for the reducing power. A value of $p<0.05$ was considered statistically significant in all cases.

Results and Discussion

Characterization of porcine gelatin-based nanoparticles containing buriti oil (OPG)

The micrographs obtained through Scanning Electron Microscopy (SEM) analysis (Fig 1A) showed particles with a spherical shape, smooth surface without cracks and physical size at the nanometer scale (<100 nm), demonstrating good protection of the core through the encapsulation. The results obtained by Castro et al.(16) for buriti oil nanoencapsulated in porcine gelatin (OPG) were similar. Lira et al.(18) obtained the same characteristics observed for shape and surface in all formulations containing quinoa oil.

Figure 1. Characterization of buriti oil nanoencapsulated in porcine gelatin. A. Micrograph obtained by Scanning Electron Microscopy. B. Dynamic light scattering. C. Fourier Transform Infrared Spectroscopy of nanoencapsulated buriti oil (a), buriti oil (b), Tween 20 (c), porcine gelatin (d).

The data obtained for particle diameter using DLS (Fig 1B) showed mean diameter and polydispersion index for OPG equal 72.0(0.68) and 0.371(0.0251), respectively. Thus, it is possible to state that OPG presented unimodal size distribution and reinforced the physical diameter observed by SEM, confirming that it is a nanoparticle. The nanometric particle size

enables the food industry to improve the bioavailability of poorly soluble substances, such as lipids and natural antioxidants(27).

Castro et al.(16) found values of 51.00 (6.07) nm and 0.40 (0.05) for particle size and polydispersion index for OPG, respectively, constituting values close to those found in this work. Particles with a large diameter and size distribution can affect the texture and compromise the incorporated bioactive compounds, which did not occur in this study. The polydispersion index less than 1 indicates homogeneity in the analyzed particle(16,28). Thus, a particle that presents itself more homogeneously is inserted in a product more easily, not affecting the texture, therefore being positive for consumer use(28,29).

Fig 1C shows the FTIR spectra obtained for buriti oil, porcine gelatin, Tween 20, and OPG. Hydrocarbon groups were detected in the buriti oil spectrum (Fig 1C, line b) by vibrational bands in the range of 2924 - 2852 cm^{-1} (C-H), in addition to bands in the region of 3007 cm^{-1} and 1462 cm^{-1}, which respectively characterize the –OH and –CH3 groups, meaning that they reflect the strong constitution of this oil described in the literature as a source ingredient of carotenoids[16]. The stretching of the vibrational band at 1743 cm^{-1} indicates the presence of double bonds in the oil chain (C = C; C = O), which shows the content of unsaturated fatty acids(30).

The vibrational band at 1651 cm^{-1} in the porcine gelatin spectrum (Fig 1C, line d) reflects the presence of the C=O bond (amide I), according to Silverstein & Webster(30). Another band is observed at 1529 cm-1, indicating amide (N-H)(31).

The spectrum of the Tween 20 surfactant (Fig 1C, line c) presented vibrations at 2920 cm^{-1} and 2864 cm^{-1} referring to stretching of the asymmetric and symmetrical methylene vibrations; at 1735 cm^{-1}, referring to the C=O connection; and at 1095 cm^{-1}, referring to the vibration stretching of $–CH_2$-O-CH_2-(30).

When observing the spectrum obtained for OPG (Fig 1C, line a), it can be seen that there was an interaction between porcine gelatin, buriti oil, and Tween 20 surfactant due to the attenuation and/or displacement of the vibrational bands that characterize the presence of the buriti oil (3007, 2924, 2852 and 1743 cm^{-1}), indicating protection of the oil in the obtained particles. The formation of new bands was also observed, which were not observed in the spectra of the raw materials (1556, 1348, and 1097 cm^{-1}), which may indicate hydrophobic interactions between buriti oil and the non-polar amino acids of porcine gelatin[16]. Castro et al.(16) also observed chemical interactions between OPG constituents, meaning buriti oil with porcine gelatin.

The encapsulation efficiency determines the oil content (%) that was successfully encapsulated inside the particles, also being an indicator of free oil present on the surface of the particles(32). The nanoformulation yield was 13.65g (1.14), and the result of the encapsulation efficiency was 89.56% (1.14), which according to the literature is considered a high efficiency, or when ≥ 80%(33,34).

Thus, this analysis confirmed the results obtained through the FTIR, which indicated a chemical interaction between gelatin, buriti oil, and Tween 20, which managed to provide greater oil retention. This result can be explained by the fact that proteins have physicochemical properties which favor forming and stabilizing emulsions in relation to carbohydrates, thus allowing greater oil retention in the particles(16,35).

Because of these characterization results, it can be stated that the process for obtaining nanoparticles used in this study is standardized and reproducible, which is essential to maintain the OPG nanoformulation properties even when obtaining new batc[illegible]. Based on this, it is possible to state that the nanoparticles produced have the same physical and chemical characteristics as those obtained by Castro et al.(16) as desired, being possible to proceed with the study for further investigation of physical and chemical characterization, safety, antioxidant potential, and antimicrobial activity.

Zeta potential

Fig 2 shows OPG Zeta Potential values with the pH variation that resulted in a change in charge density around the nanoparticle. Low zeta potential values show a tendency to settle in an aqueous medium, but this low charge indicates steric stability of OPG as they are values with charges close to zero, so they can aggregate more easily, but they also have eased to disperse(36).

Figure 2. Zeta Potential of buriti oil nanoencapsulated in porcine gelatin under different pH conditions.

As the nanoparticle is composed of porcine gelatin and has an amphoteric character, changes in pH alter the OPG surface charge between positive and negative values(17). It is noteworthy that this change in surface charge influenced by the pH of the medium is an interesting feature when working with the application of nanoparticles in biofilms(37,38).

Thermogravimetry (TG) and differential thermal analysis (DTA)

The TG/DTA curves of crude buriti oil, OPG, porcine gelatin, and Tween 20 are shown in Fig 3.

Figure 3. Thermogravimetry graphs and differential thermal analysis. a) Porcine gelatin b) Tween 20 c) Buriti oil d) OPG.

Buriti oil presents TG mass loss onset at approximately 365°C associated with three endothermic events in the DTA between 239 and 446ºC, which may be related to the complete breakdown of fatty acids(39). The total degradation of buriti oil ends at 596 ºC and thus presents two stages of mass loss, with a total loss of 98.87%. The thermal stability of buriti oil is high when compared to other vegetable oils such as coconut oil (257 ºC) and sesame oil (282 ºC)(39,40).

The OPG TG curve in Fig 3B shows an initial and progressive mass loss up to approximately 100 °C, corresponding to the endothermic peak in the DTA, which may indicate water evaporation in the sample(41). Then, the decomposition of the nanoparticle starts before the buriti oil at a temperature onset of 248 °C. After this event, mass loss occurs during the endothermic decomposition peak in both free buriti oil and nanoformulation. The mass loss is 95% and 83%, respectively, which may be related to the decomposition of the material wall(42). The OPG DTA curve presents an exothermic peak characteristic of the sample crystallization, as the XRD indicates that the sample is partially amorphous, confirming this event.

The TG curve of porcine gelatin in Fig 3C had an initial thermal degradation between 35 and 160°C, which may be related to the loss of adsorbed and bound water commonly seen with air-dried gelatin(38), confirmed by the endothermic peak in the DTA in the same temperature range. It still has the beginning of its degradation between 250 °C and 656 °C, with two stages of very evident mass loss and with a total loss of 98%. In the DTA curve, it is possible to confirm the degradation with two endothermic peaks and one exothermic peak in this same interval.

The Tween 20 TG curve in Fig 3D showed four decomposition stages. The first stage corresponds to water loss (32-196ºC) and the DTA curve presenting an endothermic peak in this temperature range, which corresponds to dehydration or desolvation. The beginning of Tween 20 degradation can be seen in the TG curve at 198 °C with the end of degradation at 654 °C, with total mass loss in the TG curve of 100%.

In evaluating the TG curves, it was possible to note that buriti oil is more stable than the other samples. However, although OPG has a lower initial degradation temperature than pure buriti oil, it is still a very high temperature for a product with a greater possibility of handling and, consequently, application than pure oil. Tween 20 and porcine gelatin may have contributed to maintaining buriti oil's stability in the form of a nanoparticle and the same amount of degradation steps. Escobar-García et al.(43) performed a thermal analysis of oil rich in free eicosapentaenoic acid (EPA) and microencapsulated in concentrated whey protein, and observed that the encapsulated sample had a delay in the degradation curve when compared to the unencapsulated sample, with this result being attributed to the protection of the encapsulating agent(44).

Table 1 shows the steps of oil decomposition, encapsulating agent, Tween 20, and nanoencapsulated. Buriti oil has two degradation stages, with the first stage between 239.09 °C - 446.21 °C with a significant mass loss of 87.45%. The initial degradation temperature is similar to the data found for buriti oil in the study of Lima et al. (231.9 °C), but it differs concerning the mass loss, which was 59.6%. The results of the studies are often contradictory, as vegetable oils

are very complex because they have a complex chemical composition, including lipid oxidation products, free fatty acids, phenolic compounds, glycerides, and non-glycerides that are present in oils in different proportions(45).

Table 1: The stages (TG) and mass loss of buriti oil, porcine gelatin, Tween 20 and OPG

Sample	Number of decomposition steps	Start decomposition temperature/ °C	End decomposition temperature /°C	Mass loss (%)
Buriti oil	1	239.09	446.21	87.45
	2	446.00	596.00	11.42
Porcine gelatin	1	38.10	166.71	10.07
	2	252.69	380.57	38.66
	3	380.57	648.62	47.20
Tween 20	1	32.84	196.17	3.80
	2	198.34	313.14	20.35
	3	313.14	432.29	71.42
	4	432.29	654.29	4.70
OPG	1	36.62	107.08	4.35
	2	218.00	388.47	44.32
	3	388.47	463.93	32.93
	4	463.93	672.59	15.93

The physicochemical characteristics in comparing buriti oils are different due to their location and other factors(46). There are disagreements among authors regarding these degradation steps. Garcia et al.(44) found only two steps for the degradation of buriti oil since the mass loss was initially high. There are some hypotheses for these differences found in vegetable oils, mainly regarding their fatty acid composition. Lima et al.(47) associated the mass losses in two stages: the triglycerides' evaporation and/or pyrolysis. Garcia et al.(44) reported the first and second weight loss as the oxidation of unsaturated and saturated fatty acids, respectively. The third step was reported as the decomposition of the polymer formed during the oxidation process.

OPG showed four degradation stages, and in the first stage, there was a mass loss of 4.35% at an initial temperature of 36.62 °C to 107.08 °C. Porcine gelatin and Tween 20 present an initial degradation temperature between 38.10 °C and 32.84 °C, respectively. This data suggests that the initial degradation observed in OPG may come from the degradation of gelatin and Tween 20. The second stage of nanoformulation degradation occurs at 218 to 388.47°C, with a mass loss of less than 50%, partially corresponding to buriti oil. This shows anticipation in the OPG mass loss compared to free buriti oil. However, the oil has a loss greater than 87%.

According to the data, it is observed that the stability of the free oil was higher in terms of initial decomposition temperature, but its degradation is very drastic, while the nanoformulation loses mass more slowly. It is important to emphasize that OPG is more stable than porcine gelatin and Tween 2 0 and that it has stability approaching that of pure buriti oil. In a way, OPG has significant advantages as it is a product with better handling, more innovative, and maintains high oil stability. These data are important for the food area since nanoencapsulation exerts the possibility of using the product at different temperatures.

Table 2 presents the data of the DTA curves for buriti oil, encapsulating agent, Tween 20, and nanoencapsulated. It is possible to observe that all samples showed four endothermic or exothermic events characteristic of the analyzed samples. DTA can accurately measure the enthalpic transition temperatures of energy absorption and release. In this case, it is observed that the buriti oil and the nanoencapsulated present four characteristic endothermic events of their decomposition. The first endothermic decomposition event in buriti oil occurs at 259.03 °C with a maximum peak of 282.98 °C (ΔH = 115.86), while this event seems to happen at a lower temperature of 214.05 °C in OPG, with a maximum peak at 245.47 ºC (ΔH=286.40), corroborating the data obtained in the TG. It is also possible to visualize the endothermic peak in the nanoformulation characteristic of the dehydration of the encapsulating agent and Tween 20 around 31 and 78 ºC.

Table 2: The stages DTA of buriti oil, porcine gelatin, Tween 20 and OPG.

Sample	Stage	T_{onset}/° C	T_{peak}/° C	**Δ**H (J/g)
Buriti oil	1	259.03 ↓	282.98	115.86
	2	350.09 ↓	381.57	2.85x10^{6*}
	3	400.41 ↓	432.56	964.53
	4	476.98 ↓	480.94	98.64
Porcine Gelatin	1	37.69 ↓	84.81	509.39
	2	215.42 ↓	227.48	12.87
	3	399.46 ↓	409.59	370.38
	4	633.63 ↑	641.97	114.80
Tween 20	1	31.77 ↓	78.14	592.78
	2	281.13 ↓	305.44	1.90x10^{6*}
	3	397.64 ↑	402.05	6.03
	4	500.21↓	523.07	354.57
OPG	1	31.78 ↓	58.95	219.36
	2	214.05 ↓	245.47	286.40
	3	387.36 ↓	431.87	6.18x10^{6*}
	4	583.34 ↓	601.43	154.45

*J/Kg; ↓ Endothermic ↑ Exothermic

Cytotoxicity - MTT assay

Determining the toxicity of nanoparticles from essential oils, vegetables and algae is necessary due to the unpredictable behavior that nanoscale materials have demonstrated(48). ISO 10993 compiles several tests on biomaterial/biological tissue interaction and determines that biocompatibility starts with evaluating material from cytotoxicity(49). Part 5 of ISO 10993 is responsible for suggesting the tests and conditions necessary for evaluating the cytotoxicity test(49). According to the standard, a reduction greater than 30% in cell viability in the tests is indicative of the material's cytotoxicity.

Fig 4 shows the MTT assay result. According to the data at the tested concentrations (0.5, 2.5, and 5 µg/ml), crude buriti oil and OPG were not cytotoxic to CHO cells, and there is no significant difference between oil a[illegible]PG. Zanatta et al.(50) evaluated the cytotoxic effect of an emulsion containing buriti oil on 3T3 cells (1000 mg/mL) and found no toxic effect, corroborating the results found in this work.

Figure 4. Cell viability (%) through the MTT assay in CHO-[illegible]ells evaluated at different times (A – 24h, B – 48h, and C – 72h) and concentrations of buriti oil and OPG.

The viability has cytotoxic potential when reduced to < 70%, which was not seen in this study(49). Therefore, the buriti oil and the nanoformulation results are favorable because it does not present toxicity to the cells, assuming that their use is safe.

Total antioxidant capacity (TAC)

TAC is determined using the phosphomolybdenum blue complex based on reducing Mo^{+6} to Mo^{+5} by antioxidant compounds and the formation of green Mo^{+5} complexes(51). The results obtained (Table 3) showed that the nanoencapsulation process increased the antioxidant capacity of buriti oil threefold. This result can be explained by the greater dispersibility in water observed for the OPG due to the obtained particle size, suggesting an increase in the contact surface for chemical interaction with water, and consequently increased the antioxidant activity of the encapsulate (16,18).

Table 3 – Total antioxidant capacity of buriti oil and OPG

Samples	mg AA.g^{-1}
Buriti oil	14.60 (1.63)^{a}

OPG	48.34 (3.71)^{b}

OPG: buriti oil nanoencapsulated in porcine gelatin
AA: ascorbic acid
Mean and standard deviation (SD), n = 3. The different lowercase letters indicate a statistical difference.

TAC analyzes for crude and encapsulated buriti oil were not found in the literature. Ribeiro et al.(52) evaluated the total antioxidant capacity of oil from faveleira seeds. The value found was 0.04 (0.00) mg AA/g, constituting a lower result than its methanolic fraction [0.08 (0.00) mg AA/g] and higher than its non-polar fraction [0.02 (0.00) mg AA/g].

Silva et al.(53) extracted astaxanthin from shrimp of the *Litopenaeus vannamei* species using soybean oil and evaluated the antioxidant potential. The TAC results showed that the astaxanthin-pigmented soybean oil had higher antioxidant activity than soybean oil (control).

Zhong and Shahidi(54) compared the antioxidant activities of polar and non-polar compounds and observed that a higher concentration of non-polar samples is needed to achieve the optimal antioxidant activity. These data corroborate this study since the buriti oil concentration required to present antioxidant activity was higher than that of OPG, which has a solubility in water.

Reducing power

The reducing power assesses the ability of a sample to donate electrons in the presence of ferric chloride under acidic conditions and thus reduce Fe^{+3} to Fe^{+2}(55). The results (Fig 5) showed that a lower OPG concentration than crude buriti oil was necessary to present similar antioxidant activity. Therefore, it is noteworthy that there is 0.98 mg of buriti oil in 2 mg/mL of OPG according to the encapsulation efficiency and considering the amount and proportion of the encapsulating agents, and it is possible to note that this concentration was sufficient to promote a reduction from Fe^{+3} to Fe^{+2} in 100%.

Figure 5. Test of the reducing power of crude buriti oil (A) and OPG nanoformulat[illegible] The data obtained presented parametric distribution. Therefore, the ANOVA test with Tukey's post-test was used to determine the significant differences. *Equal letters indicate that the values do not differ statistically ($p > 0.05$).

On the other hand, 1 mg/mL of crude buriti oil promoted a reduction from Fe^{+3} to Fe^{+2} by only 0.74%. The encapsulate concentration must take into account that there are also encapsulating agents in addition to the oil (swine gelatin and Tween 20 in a ratio of 1:2.15). In addition, encapsulation efficiency must also be considered, as not all of the oil placed in the system was encapsulated.

Based on this, the result obtained demonstrates that the nanoencapsulation process enhanced the reducing power of buriti oil, with the reduction being greater as the concentration

used in the test increases. No reduction power studies were found for crude or encapsulated buriti oil. In analyzing turmeric essential oil, Mau et al.(56) observed that the reducing power increases as the concentration of oil increases, corroborating the data found in this work.

Antioxidant activity by 2,2'-azinobis-3-ethylbenzothiazoline-6-sulfonic acid (ABTS•)

Nanoencapsulation is known to increase the antioxidant potential of natural molecules(57–59). ABTS radical scavenging was evaluated using buriti oil and OPG (Table 4).

Table 4 – Antioxidant activity (IC 50) buriti oil and OPG.

**Samples**	**IC50 (mg. mL^{-1})**
Buriti oil	4.18 (0.05)^{a}
OPG	0.94 (0.02)^{b}

OPG: buriti oil nanoencapsulated in porcine gelatin
Mean and standard deviation (SD), n = 3. The different lowercase letters indicate a statistical difference.

The results obtained demonstrate that the technological process of nanoencapsulation favored the antioxidant activity since the amount of OPG used was about four times smaller than that of buriti oil to reach the IC50.

Lira et al.(18) performed an analysis for crude quinoa oil and its three nanoencapsulated formulations (encapsulated with porcine gelatin, encapsulated with whey protein and porcine gelatin in aqueous phase 1, and encapsulated with whey protein in aqueous phase 1 and gelatin in aqueous phase 2). The IC 50 of the formulations was lower than that of the oil, corroborating the data found in this study.

Eugenol essential oil (EO) was nanoencapsulated by the O/W emulsification method with sodium caseinate, maltodextrin and Tween 80(60). The results showed that the nanoencapsulated eugenol EO had a lower IC50 for the ABTS radical assay than crude eugenol(60).

This analysis showed that the OPG nanoparticle has greater antioxidant activity than its unencapsulated form. This difference is relevant for its applications in food matrices as a way to prolong the biological action and provide health benefits (58,61).

Determination of the modulating activity of antibiotics

Due to the emergence of multidrug-resistant strains to antibiotics, a strategy that has been used against these resistance mechanisms is the use of combined drugs(62). One example is the use of plant extracts that exhibit synergistic activity against microorganisms, as they present

a complex mixture of natural products and present a low risk in the development of bacterial resistance(63). Some natural products such as plant extracts, essential oils(64) and fixed oils(65) have modified the activity of antibiotics. Fig 6 shows the result of the MIC of buriti oil and OPG combined with norfloxacillin and gentamycin antibiotics.

Figure 6. MIC values (µg/mL) of antibiotics in a bacterial growth assay in the presence of buriti oil (A) and buriti oil nanoencapsulated in porcine gelatin (OPG) (B). The tests were performed under the following conditions: norfloxacillin (white bar), norfloxacillin + crude buriti oil/OPG (light gray bar), gentamicin (dark gray bar) and gentamicin + crude buriti oil/OPG (black bar). ***p<0.0001.

Gentamycin is a drug used to treat infections, especially by gram-negative bacteria, such as *E. coli* and *P. aeruginosa*, and it can also be used in combination(66). Its MIC for *E. coli* and *P. aeruginosa* is 1 µg/ml and 2 µg/ml, respectively(67,68). Norfloxacin is a broad-spectrum antibiotic, used for gram-positive and gram-negative bacteria(69); it has a MIC of 25 µg/ml for *E. coli*, 2 µg/ml for *P. aeruginosa* and 128 µg/ ml for *S. aureus*(70–72). Fixed buriti oil has a MIC >1024 µg/ml for several strains, including *E. coli*, *P. aeruginosa* and *S. aureus*(73).

Modulation with vegetable oils promotes better action on bacteria when there is a synergism of this combination, reducing the amount or increasing the amount of medication used(73). The mechanisms of action between antibiotic and oil(74,75) may be related to the interaction with the lipid bilayer and cell membrane, affecting the respiratory chain and energy production by the bacteria(76), or even interference with bacterial enzymatic systems that may enhance the mechanism of action of antibiotics in combination with oils(77).

The combination of antibiotics with crude buriti oil (Fig 5A) and OPG (Fig 6B) showed greater efficiency in inhibiting the action of the *E. coli* bacteria when compared to isolated antibiotics. The combination of norfloxacillin and gentamycin antibiotics with the crude buriti oil reduced the MIC by 93.75% and 75%, respectively. Combining the same antibiotics with OPG reduced the MIC of norfloxacillin by 98% and gentamycin by 75% in *E. coli*. For gentamycin, the nanoencapsulated oil maintained the modulating activity of the crude oil in 75%.

Leão et al.(78) observed that a nanoemulsion containing buriti oil inhibited the microbial growth of *E. coli* by 61% at a concentration of 3.14 mg/mL, demonstrating a bacteriostatic effect. With this, the researchers observed that the physicochemical characteristics of the nanoemulsion contribute to its biological activity. The particle size can directly affect the antimicrobial activity of the emulsion based on essential oils, meaning a smaller size (nanometric scale) induces a greater inactivation of *E. coli* when compared to conventional emulsions(78).

Norfloxacillin alone, when compared with norfloxacillin combined with crude buriti oil (Fig 6A), did not present modulating activity for *S. aureus* with a statistically significant difference (p>0.05). However, the antibiotic activity combined with OPG decreased the MIC by 50% (Fig 6B). For gentamycin combined with crude oil such as OPG, there was an antagonism characterized by an increase in MIC by 100% in both demonstrated cases. Gentamycin does not

act on Gram-positive bacteria, as is the case with *S. aureus*(66), demonstrating that the nanoformulation maintained the crude buriti oil activity. Two main mechanisms are involved in the production of antagonistic effects associated with natural products and antibiotics: chelation of antibiotic constituents by the natural product and competition of substances for the same binding site(79).

Norfloxacillin combined with buriti oil and OPG had greater inhibition for *P. aeruginosa* bacteria than with norfloxacillin alone, with a statistical difference between them ($p<0.05$) and a 99% reduction in MIC with OPG and 97% with crude buriti oil. Gentamycin showed a significant difference in the two combinations (with free oil and OPG), in which there was a 50% reduction in MIC. In this case, both the free oil and OPG had a synergistic effect with the antibiotics.

Oils modify the antimicrobial effect depending on the antibiotic, oil tested and the bacterial species analyzed(80), which can be attributed to an interaction of the oil with the plasma membrane of the bacteria, increasing the permeability of the plasma membrane to the antibiotic(80–82).

Pereira et al.(73) used two antibiotics (gentamycin and amikacin) and two bacterial strains (*S. aureus* and *E. coli*) to verify the modulating activity of fixed buriti oil. The synergistic effect was observed in both antibiotics for *S. aureus*, with no MIC changes in *E. coli*.

Combinations of natural products and antibiotics(83) can affect more than one target for bacterial growth inhibition. This strategy is known as “herbal shotgun” or “synergistic multi-effect targeting” in which different therapeutic components collaborate in a synergistic-agonistic way(79).

Thus, the present study showed promising results for using free buriti oil and OPG in association with the modification of antibiotic resistance against multiresistant strains such as *E. coli* and *P. aeroginosa*. There are no reports in the literature of encapsulated oils and modulating antimicrobial activity, and therefore this is a preliminary study.

Conclusions

With the results obtained for the characterization, it can be seen that the particles presented physical and chemical characteristics similar to those obtained in a previous study by our research group, which contributed to maintaining the evaluated biological and modulating activities.

The crude buriti oil and the OPG nanoformulation did not demonstrate cytotoxic effects for the tested cell line. In addition, the oil showed antioxidant activity enhanced by the applied technological process. Buriti oil and nanoformulation showed positive results in combination with drugs in the modulating activity with antibiotics, decreasing the MIC in most combinations. However, the nanoformulation stood out in relation to free oil and for promoting a reduction in MIC when combined with norfloxacillin, inhibiting the *S. aureus* bacteria.

With this, OPG presents itself as a viable alternative to promote the preservation and/or enhancement of the oil's bioactive properties, which favors its use by the food and pharmaceutical industry, in addition to generating benefits to consumers' health. The application of nanotechnology in materials of plant origin promotes valorization of this resource and can contribute to the local development of the buruti-producing region within the perspective of circular economy and sustainable production.

References

1. Cattani IM, Baruque-Ramos J. Brazilian buriti palm fiber (Mauritia flexuosa Mart.). In Natural fibres: Advances in science and technology towards industrial applications. Springer, Dordrecht. 2016;89–98.

2. Cândido TLNN, Silva MR, Agostini-Costa TS. Bioactive compounds and antioxidant capacity of buriti (Mauritia flexuosa L.f.) from the Cerrado and Amazon biomes. Food Chem [Internet]. 2015;177:313–9. Available from: http://dx.doi.org/10.1016/j.foodchem.2015.01.041

3. Cândido TLN, Silva MR. Comparison of the physicochemical profiles of buriti from the Brazilian Cerrado and the Amazon region. Food Sci Technol. 2017;37:78–82.

4. Silva SM, Sampaio KA, Taham T, Rocco SA, Ceriani R, Meirelles AJA. Characterization of oil extracted from buriti fruit (Mauritia flexuosa) grown in the Brazilian Amazon Region. JAOCS, J Am Oil Chem Soc. 2009;86(7):611–6.

5. Ferreira BS, De Almeida CG, Faza LP, De Almeida A, Diniz CG, Da Silva VL, et al. Comparative properties of amazonian oils obtained by different extraction methods. Molecules. 2011;16(7):5874–85.

6. de Souza FG, de Araújo FF, de Paulo Farias D, Zanotto AW, Neri-Numa IA, Pastore GM. Brazilian fruits of Arecaceae family: An overview of some representatives with promising food, therapeutic and industrial applications. Food Res Int. 2020;138(September).

7. Zanatta CF, Mitjans M, Urgatondo V, Rocha-Filho PA, Vinardell MP. Photoprotective potential of emulsions formulated with Buriti oil (Mauritia flexuosa) against UV irradiation on keratinocytes and fibroblasts cell lines. Food Chem Toxicol [Internet]. 2010;48(1):70–5. Available from: http://dx.doi.org/10.1016/j.fct.2009.09.017

8. Aquino JDS, Vasconcelos MHDA, Pessoa DCNDP, Soares JKB, Prado JPDS, Mascarenhas RDJ, et al. Intake of cookies made with buriti oil (: Mauritia flexuosa) improves vitamin A status and lipid profiles in young rats. Food Funct. 2016;7(10):4442–50.

9. Hamacek FR, Della Lucia CM, Silva BP da, Moreira AVB, Pinheiro-Sant'ana HM. Buriti of the cerrado of Minas Gerais, Brazil: physical and chemical characterization and content of carotenoids and vitamins. Food Sci Technol. 2018;38(suppl 1):263–9.

10. Galli F, Azzi A, Birringer M, Cook-Mills JM, Eggersdorfer M, Frank J, et al. Vitamin E: Emerging aspects and new directions. Free Radic Biol Med [Internet]. 2017;102(June 2016):16–36. Available from: http://dx.doi.org/10.1016/j.freeradbiomed.2016.09.017

11. Leslie MA, Cohen DJA, Liddle DM, Robinson LE, Ma DWL. A review of the effect of omega-3 polyunsaturated fatty acids on blood triacylglycerol levels in normolipidemic and borderline hyperlipidemic individuals. Lipids Health Dis [Internet]. 2015;14(1). Available from: http://dx.doi.org/10.1186/s12944-015-0049-7

12. Fuentes E, Rodríguez-Pérez W, Guzmán L, Alarcón M, Navarrete S, Forero-Doria O, et al. Mauritia flexuosa presents in vitro and in vivo antiplatelet and antithrombotic activities. Evidence-based Complement Altern Med. 2013;2013.

13. Moser P, Ferreira S, Nicoletti VR. Buriti oil microencapsulation in chickpea protein/pectin matrix as affected by spray drying parameters. Food Bioprod Process [Internet]. 2019;34. Available from: https://linkinghub.elsevier.com/retrieve/pii/S0960308519303724

14. Santana AA, Kurozawa LE, de Oliveira RA, Park KJ. Influence of Process Conditions on the Physicochemical Properties of Pequi Powder Produced by Spray Drying. Dry Technol. 2013;31(7):825–36.

15. Sharma S, Cheng S-F, Bhattacharya B, Chakkaravarthi S. Efficacy of free and encapsulated natural antioxidants in oxidative stability of edible oil: Special emphasis on nanoemulsion-based encapsulation. Trends Food Sci Technol [Internet]. 2019;91:305–18. Available from: https://doi.org/10.1016/j.tifs.2019.07.030

16. Castro GMMA, Passos TS, Nascimento SS da C, Medeiros I, Araújo NK, Maciel BLL, et al. Gelatin nanoparticles enable water dispersibility and potentialize the antimicrobial activity of Buriti (Mauritia flexuosa) oil. BMC Biotechnol [Internet]. 2020 Dec 16;20(55):13. Available from: https://bmcbiotechnol.biomedcentral.com/articles/10.1186/s12896-020-00649-4

17. Kaushik P, Dowling K, Barrow CJ, Adhikari B. Microencapsulation of omega-3 fatty acids: A review of microencapsulation and characterization methods. J Funct Foods [Internet]. 2015;19:868–81. Available from: http://dx.doi.org/10.1016/j.jff.2014.06.029

18. Lira KHD da S, Passos TS, Ramalho HMM, Rodrigues KD da SR, Vieira É de A, Cordeiro AMT de M, et al. Whey protein isolate-gelatin nanoparticles enable the water-dispersibility and potentialize the antioxidant activity of quinoa oil (Chenopodium quinoa). PLoS One. 2020;15(10 October):1–17.

19. Delshadi R, Bahrami A, Tafti AG, Barba FJ, Williams LL. Micro and nano-encapsulation of vegetable and essential oils to develop functional food products with improved nutritional profiles. Trends Food Sci Technol [Internet]. 2020;104(May):72–83. Available from: https://doi.org/10.1016/j.tifs.2020.07.004

20. Medeiros AK de OC, Gomes C de C, Amaral MLQ de A, Medeiros LDG de, Medeiros I, Porto DL, et al. Nanoencapsulation improved water solubility and color stability of carotenoids extracted from Cantaloupe melon (Cucumis melo L.). Food Chem [Internet]. 2019;270(April 2018):562–72. Available from: https://doi.org/10.1016/j.foodchem.2018.07.099

21. El-Messery TM, Altuntas U, Altin G, Özçelik B. The effect of spray-drying and freeze-drying on encapsulation efficiency, in vitro bioaccessibility and oxidative stability of krill oil nanoemulsion system. Food Hydrocoll. 2020;106(March).

22. Prieto P, Pineda M, Aguilar M. Spectrophotometric quantitation of antioxidant capacity through the formation of a …. Anal Biochem [Internet]. 1999;269:337–41. Available from: http://linkinghub.elsevier.com/retrieve/pii/S0003269799940198

23. Wang J, Zhang Q, Zhang Z, Li Z. Antioxidant activity of sulfated polysaccharide fractions extracted from Laminaria japonica. Int J Biol Macromol. 2008;42(2):127–32.

24. Rufino M do S, Alves RE, Brito ES De, Morais SM De, Sampaio CDG, Saura-calixto FD. Metodologia Científica: Determinação da Atividade Antioxidante Total em Frutas pela Captura do Radical Livre ABTS +. Comun Técnico Embrapa. 2007;128:0–3.

25. Bezerra CF, Camilo CJ, do Nascimento Silva MK, de Freitas TS, Ribeiro-Filho J, Coutinho HDM. Vanillin selectively modulates the action of antibiotics against resistant bacteria. Microb Pathog [Internet]. 2017;113(October):265–8. Available from: https://doi.org/10.1016/j.micpath.2017.10.052

26. Patel J.B., Cockerill R.F., Bradford A.P., Eliopoulos M.G., Hindler A.J., Jenkins G.S., Lewis S.J., Limbago B., Miller A.L., Nicolau P.D., Pwell M., Swenson M.J., Traczewski M.M., Turnidge J.D. WPMZLB. M07-A10: Methods for Dilution Antimicrobial Susceptibility Tests for Bacteria That Grow Aerobically; Approved Standard—Tenth Edition. CLSI (Clinical Lab Stand Institute). 2015;35(2).

27. Chen L, Remondetto GE, Subirade M. Food protein-based materials as nutraceutical delivery systems. Trends Food Sci Technol. 2006;17(5):272–83.

28. Kumar A, Dixit CK. Methods for characterization of nanoparticles. In: Advances in Nanomedicine for the Delivery of Therapeutic Nucleic Acids. 2017. p. 44–58.

29. Danaei M, Dehghankhold M, Ataei S, Hasanzadeh Davarani F, Javanmard R, Dokhani A, et al. Impact of particle size and polydispersity index on the clinical applications of lipidic nanocarrier systems. Pharmaceutics. 2018;10(2):1–17.

30. Silverstein RM, Webster FX, Kiemle DJ. Silverstein-Spectrometric Identification of Organic Compounds 7th ed. 7th ed. New York: John Wiley & Sons; 2005. 550 p.

31. Guerrero P, Stefani PM, Ruseckaite RA, De La Caba K. Functional properties of films based on soy protein isolate and gelatin processed by compression molding. J Food Eng [Internet]. 2011;105(1):65–72. Available from: http://dx.doi.org/10.1016/j.jfoodeng.2011.02.003

32. Esfahani R, Jafari SM, Jafarpour A, Dehnad D. Loading of fish oil into nanocarriers prepared through gelatin-gum Arabic complexation. Food Hydrocoll [Internet]. 2019;90(December 2018):291–8. Available from: https://doi.org/10.1016/j.foodhyd.2018.12.044

33. Batista JS, Olinda RG, Medeiros VB, Rodrigues CMF, Oliveira AF, Paiva ES, et al. Atividade antibacteriana e cicatrizante do óleo de buriti Mauritia flexuosa L. Ciência Rural [Internet]. 2012;42(1):136–41. Available from: http://www.scielo.br/pdf/cr/2011nahead/a1012cr5053

34. Sagiri SS, Anis A, Pal K. Review on Encapsulation of Vegetable Oils: Strategies, Preparation Methods, and Applications. Polym - Plast Technol Eng [Internet]. 2016;55(3):291–311. Available from: http://dx.doi.org/10.1080/03602559.2015.1050521

35. Can Karaca A, Low NH, Nickerson MT. Potential use of plant proteins in the microencapsulation of lipophilic materials in foods. Trends Food Sci Technol [Internet]. 2015;42(1):5–12. Available from: http://dx.doi.org/10.1016/j.tifs.2014.11.002

36. da Silva BC, de Oliveira M, Ferreira JGL, Sierakowski MR, Simas-Tosin FF, Orth ES, et al. Polyelectrolyte complexes from gum arabic and gelatin: Optimal complexation pH as a key parameter to obtain reproducible microcapsules. Food Hydrocoll. 2015;46:201–7.

37. Wu Y, Guo J, Yang W, Wang C, Fu S. Preparation and characterization of chitosan-poly(acrylic acid) polymer magnetic microspheres. Polymer (Guildf). 2006;47(15):5287–94.

38. Zhao Y, Sun Z. Effects of gelatin-polyphenol and gelatin–genipin cross-linking on the structure of gelatin hydrogels. Int J Food Prop [Internet]. 2018;20(3):S2822–32. Available from: https://doi.org/10.1080/10942912.2017.1381111

39. Durães JA, Drummond AL, Pimentel TAPF, Murta MM, Moreira SGC, Sales MJA. Thermal and structural behavior of Buriti oil/poly(methyl methacrylate) and Buriti oil/polystyrene materials. J Therm Anal Calorim. 2008;92(2):529–34.

40. Jayadas NH, Nair KP. Coconut oil as base oil for industrial lubricants-evaluation and modification of thermal, oxidative and low temperature properties. Tribol Int. 2006;39(9):873–8.

41. Klein T, Longhini R, Bruschi ML, De Mello JCP. Microparticles containing Guaraná extract obtained by spray-drying technique: Development and characterization. Rev Bras Farmacogn [Internet]. 2015;25(3):292–300. Available from: http://dx.doi.org/10.1016/j.bjp.2015.03.010

42. Liz GR de, Verruck S, Canella MHM, Dantas A, Garcia SG, Maran BM, et al. Stability of bifidobacteria entrapped in goat's whey freeze concentrate and inulin as wall materials and powder properties. Food Res Int. 2020;127(February 2019):10.

43. Escobar-García JD, Prieto C, Pardo-Figuerez M, Lagaron JM. Room temperature nanoencapsulation of bioactive eicosapentaenoic acid rich oil within whey protein microparticles. Nanomaterials. 2021;11(3):1–16.

44. Garcia CC, Franco PIBM, Zuppa TO, Filho NRA, Leles MIG. Thermal stability studies of some cerrado plant oils. J Therm Anal Calorim. 2007;87(3):645–8.

45. Vecchio S, Cerretani L, Bendini A, Chiavaro E. Thermal decomposition study of monovarietal extra virgin olive oil by simultaneous thermogravimetry/differential scanning calorimetry: Relation with chemical composition. J Agric Food Chem. 2009;57(11):4793–800.

46. Nde DB, Anuanwen CF. Optimization methods for the extraction of vegetable oils: A review. Processes. 2020;8(2):21.

47. Pereira Lima R, Souza da Luz PT, Braga M, dos Santos Batista PR, Ferreira da Costa CE, Zamian JR, et al. Murumuru (Astrocaryum murumuru Mart.) butter and oils of buriti (Mauritia flexuosa Mart.) and pracaxi (Pentaclethra macroloba (Willd.) Kuntze) can be used for biodiesel production: Physico-chemical properties and thermal and kinetic studies. Ind Crops Prod [Internet]. 2017;97:536–44. Available from: http://dx.doi.org/10.1016/j.indcrop.2016.12.052

48. Schappo FB. Nanopartículas de óleo de palma bruto: estabilidade e citotoxicidade. Programa de Pós-Gaduação em Ciência dos Alimentos. Universidade Federala de Santa Catarina; 2021.

49. International Organization for Standardization. ISO 10993-5: Tests for in vitro cytotoxicity. In: Biological evaluation of medical devices. 2009. p. 42.

50. Zanatta CF, Ugartondo V, Mitjans M, Rocha-Filho PA, Vinardell MP. Low cytotoxicity of creams and lotions formulated with Buriti oil (Mauritia flexuosa) assessed by the neutral red release test. Food Chem Toxicol. 2008;46(8):2776–81.

51. Melo-Silveira RF, Fidelis GP, Pereira Costa MSS, Telles CBS, Dantas-Santos N, de Oliveira Elias S, et al. In vitro antioxidant, anticoagulant and antimicrobial activity and in inhibition of cancer cell proliferation by xylan extracted from corn cobs. Int J Mol Sci. 2012;13(1):409–26.

52. Ribeiro PPC, E Silva DMDL, De Assis CF, Correia RTP, Damasceno KSFDSC. Bioactive properties of faveleira (Cnidoscolus quercifolius) seeds, oil and press cake obtained during oilseed processing. PLoS One. 2017;12(8):1–12.

53. Silva DMLE, Damasceno KSFSC, Ribeiro PPC, Alcântra MA, Cordeiro AMTM, Passos TS, et al. Chemical characteristics and antioxidant activity of astaxanthin extracted from shrimp residues using soybean oil. J Braz Chem Soc. 2021;32(6):1277–85.

54. Zhong Y, Shahidi F. Antioxidant behavior in bulk oil: Limitations of polar paradox theory. J Agric Food Chem. 2012;60(1):4–6.

55. Melo-Silveira RF, Fidelis GP, Viana RLS, Soeiro VC, Da Silva RA, Machado D, et al. Antioxidant and antiproliferative activities of methanolic extract from a neglected agricultural product: Corn cobs. Molecules. 2014;19(4):5360–78.

56. Mau JL, Lai EYC, Wang NP, Chen CC, Chang CH, Chyau CC. Composition and antioxidant activity of the essential oil from Curcuma zedoaria. Food Chem. 2003;82(4):583–91.

57. Dos Santos PDF, Francisco CRL, Coqueiro A, Leimann FV, Pinela J, Calhelha RC, et al. The nanoencapsulation of curcuminoids extracted from: Curcuma longa L. and an evaluation of their cytotoxic, enzymatic, antioxidant and anti-inflammatory activities. Food Funct. 2019;10(2):573–82.

58. Huang X, Liu Y, Zou Y, Liang X, Peng Y, McClements DJ, et al. Encapsulation of resveratrol in zein/pectin

core-shell nanoparticles: Stability, bioaccessibility, and antioxidant capacity after simulated gastrointestinal digestion. Food Hydrocoll [Internet]. 2019;93(December 2018):261–9. Available from: https://doi.org/10.1016/j.foodhyd.2019.02.039

59. Luo Y. Food colloids binary and ternary nanocomplexes: Innovations and discoveries. Colloids Surfaces B Biointerfaces [Internet]. 2020;196(August):111309. Available from: https://doi.org/10.1016/j.colsurfb.2020.111309

60. Nagaraju PG, Sindhu P, Dubey T, Chinnathambi S, Poornima Priyadarshini CG, Rao PJ. Influence of sodium caseinate, maltodextrin, pectin and their Maillard conjugate on the stability, in vitro release, anti-oxidant property and cell viability of eugenol-olive oil nanoemulsions. Int J Biol Macromol [Internet]. 2021;183:158–70. Available from: https://doi.org/10.1016/j.ijbiomac.2021.04.122

61. Bilenler T, Gokbulut I, Sislioglu K, Karabulut I. Antioxidant and antimicrobial properties of thyme essential oil encapsulated in zein particles. Flavour Fragr J. 2015;30(5):392–8.

62. Coutinho HDM, Costa JGM, Falcão-Silva VS, Siqueira-Júnior JP, Lima EO. Effect of Momordica charantia L. in the resistance to aminoglycosides in methicilin-resistant Staphylococcus aureus. Comp Immunol Microbiol Infect Dis. 2010;33(6):467–71.

63. Daferera DJ, Ziogas BN, Polissiou MG. The effectiveness of plant essential oils on the growth of Botrytis cinerea, Fusarium sp. and Clavibacter michiganensis subsp. michiganensis. Crop Prot. 2003;22(1):39–44.

64. Coutinho HDM, Costa JGM, Lima EO, Falcão-Silva VS, Siqueira JP. Herbal therapy associated with antibiotic therapy: Potentiation of the antibiotic activity against methicillin - Resistant Staphylococcus aureus by Turnera ulmifolia L. BMC Complement Altern Med. 2009;9:1–4.

65. Al Ashaal HA, Farghaly AA, Abd El Aziz MM, Ali MA. Phytochemical investigation and medicinal evaluation of fixed oil of Balanites aegyptiaca fruits (Balantiaceae). J Ethnopharmacol. 2010;127(2):495–501.

66. Chen C, Chen Y, Wu P, Chen B. Update on new medicinal applications of gentamicin: Evidence-based review. J Formos Med Assoc [Internet]. 2014;113(2):72–82. Available from: http://dx.doi.org/10.1016/j.jfma.2013.10.002

67. Garrod LP, Waterworth PM. Effect of medium composition on the apparent sensitivity of Pseudomonas aeruginosa to gentamicin. J Clin Pathol. 1969;22(5):534–8.

68. Medeiros AA, O'Brien TF, Wacker WEC, Yulug NF. Effect of salt concentration on the apparent in-vitro susceptibility of pseudomonas and other gram-negative bacilli to gentamicin. J Infect Dis. 1971;124(December):S59–64.

69. Chierentin L, Salgado HRN. Review of Properties and Analytical Methods for the Determination of Norfloxacin. Crit Rev Anal Chem. 2016;46(1):22–39.

70. Aoyama H, Sato K, Kato T, Hirai K, Mitsuhashi S. Norfloxacin resistance in a clinical isolate of Escherichia coli. Antimicrob Agents Chemother. 1987;31(10):1640–1.

71. King A, Warren C, Shannon K, Phillips I. In vitro antibacterial activity of norfloxacin (MK-0366). Antimicrob Agents Chemother. 1982;21(4):604–7.

72. Diniz-Silva HT, Magnani M, de Siqueira S, de Souza EL, de Siqueira-Júnior JP. Fruit flavonoids as modulators of norfloxacin resistance in Staphylococcus aureus that overexpresses norA. LWT - Food Sci Technol. 2017;85:324–6.

73. Faustino Pereira Y, do Socorro Costa M, Relison Tintino S, Esmeraldo Rocha J, Fernandes Galvão Rodrigues F, de Sá Barreto Feitosa M, et al. Modulation of the Antibiotic Activity by the Mauritia flexuosa (Buriti) Fixed Oil against Methicillin-Resistant Staphylococcus Aureus (MRSA) and Other Multidrug-Resistant (MDR) Bacterial Strains. Pathogens. 2018;7(4):98.

74. Coutinho HDM, Costa JGM, Siqueira-Júnior JP, Lima EO. In vitro anti-staphylococcal activity of Hyptis martiusii Benth against methicillin-resistant Staphylococcus aureus-MRSA strains. Rev Bras Farmacogn. 2008;18(SUPPL.):670–5.

75. Coutinho HDM, Costa JGM, Lima EO, Falcão-Silva VS, Siqueira JP. Enhancement of the antibiotic activity against a multiresistant Escherichia coli by Mentha arvensis L. and chlorpromazine. Chemotherapy. 2008;54(4):328–30.

76. Nicolson K, Evans G, O'Toole PW. Potentiation of methicillin activity against methicillin-resistant Staphylococcus aureus by diterpenes. FEMS Microbiol Lett. 1999;179(2):233–9.

77. Wendakoon CN, Morihiko S. Inhibition of amino acid decarboxylase activity of enterobacter aerogenes by active components in spices. Vol. 58, Journal of Food Protection. 1995. p. 280–3.

78. Leão KMM, Reis LVC, Speranza P, Rodrigues AP, Ribeiro APB, Macedo JA, et al. Physicochemical characterization and antimicrobial activity in novel systems containing buriti oil and structured lipids nanoemulsions. Biotechnol Reports. 2019;24.

79. Wagner H, Ulrich-Merzenich G. Synergy research: Approaching a new generation of phytopharmaceuticals.

Phytomedicine. 2009;16(2–3):97–110.

80. Oliveira RAG de, Lima EO, Vieira WL, Freire KRL, Trajano VN, Lima IO, et al. Estudo da interferência de óleos essenciais sobre a atividade de alguns antibióticos usados na clínica. Rev Bras Farmacogn. 2006;16(1):77–82.

81. Medeiros Barreto H, Cerqueira Fontinele F, Pereira De Oliveira A, Arcanjo DDR, Cavalcanti Dos Santos BH, De Abreu APL, et al. Phytochemical prospection and modulation of antibiotic activity in vitro by lippia origanoides H.B.K. in methicillin resistant staphylococcus aureus. Biomed Res Int. 2014;2014.

82. Sousa EO, Barreto FS. Atividade antibacteriana e interferência de Lantana camara L. e Lantana montevidensis (Spreng.) Briq. na resistência de aminoglicosídeos. Rev Bras Biociências. 2011;9(1):1–5.

83. Matias EEF, Santos KKA, Almeida TS, Costa JGM, Coutinho HDM. Enhancement of Antibiotic Activity by Cordia verbenacea DC. Lat Am J Pharm. 2010;29:1049–52.

Figure

Click here to access/download;Figure;Figura 1.tif

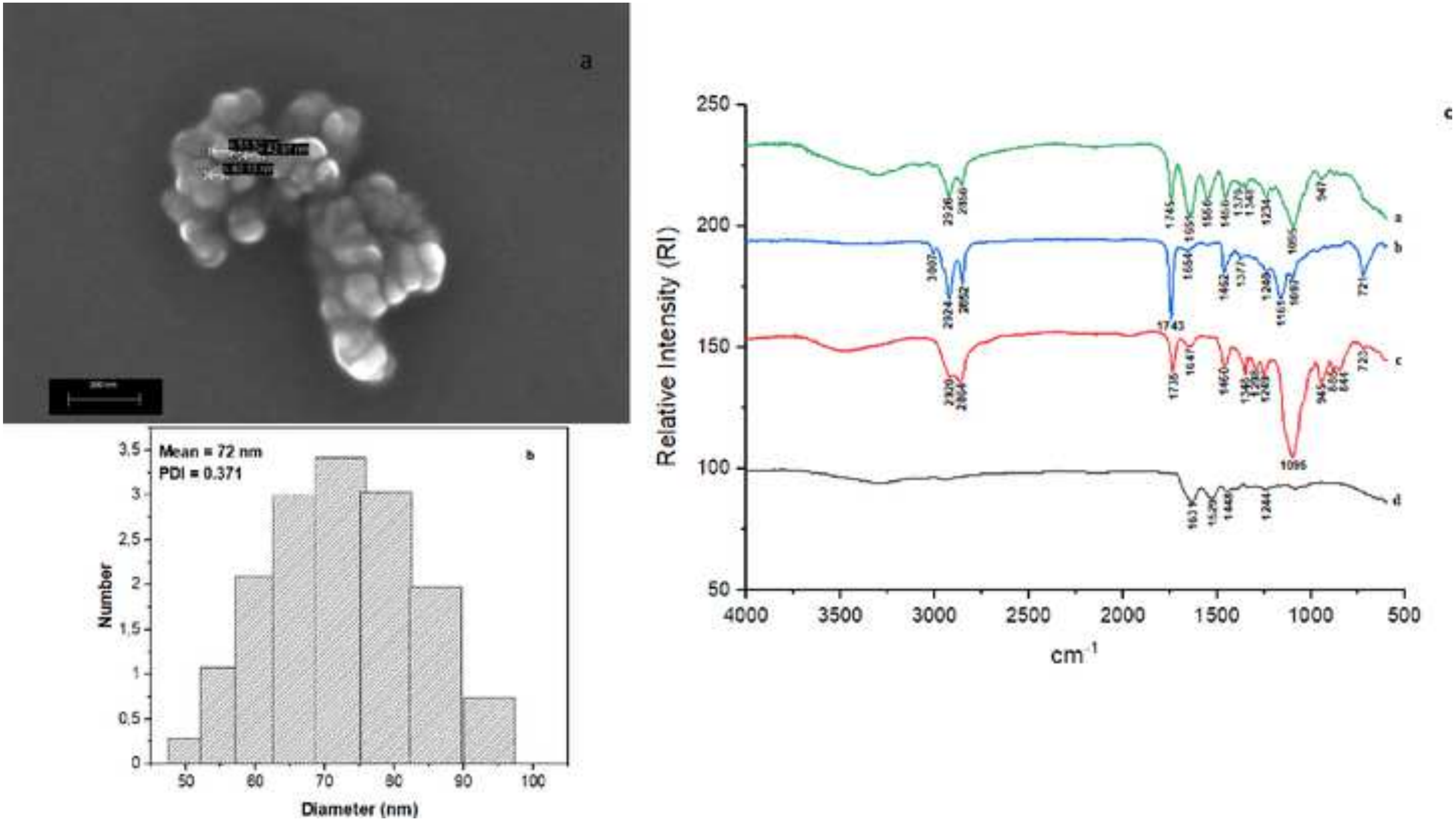

Figure

Click here to access/download;Figure;Figura 2.tif

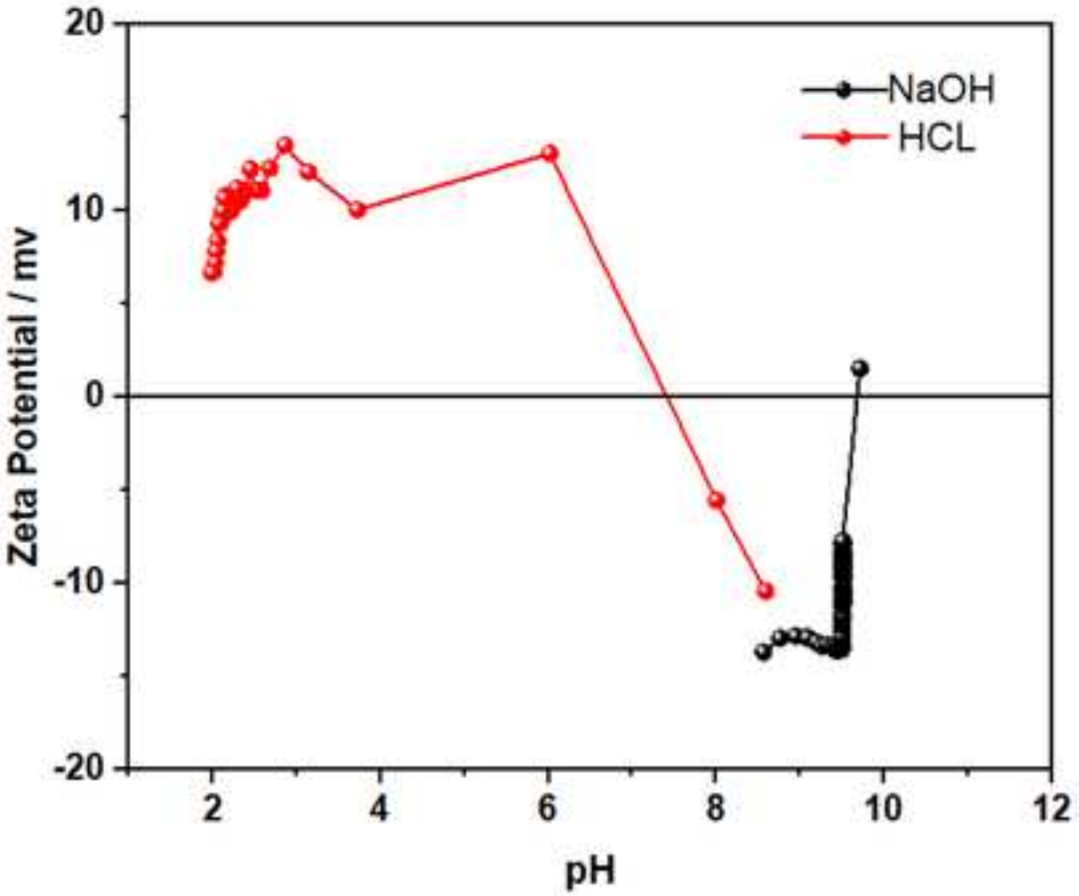

Figure Click here to access/download;Figure;Figure 3.tif

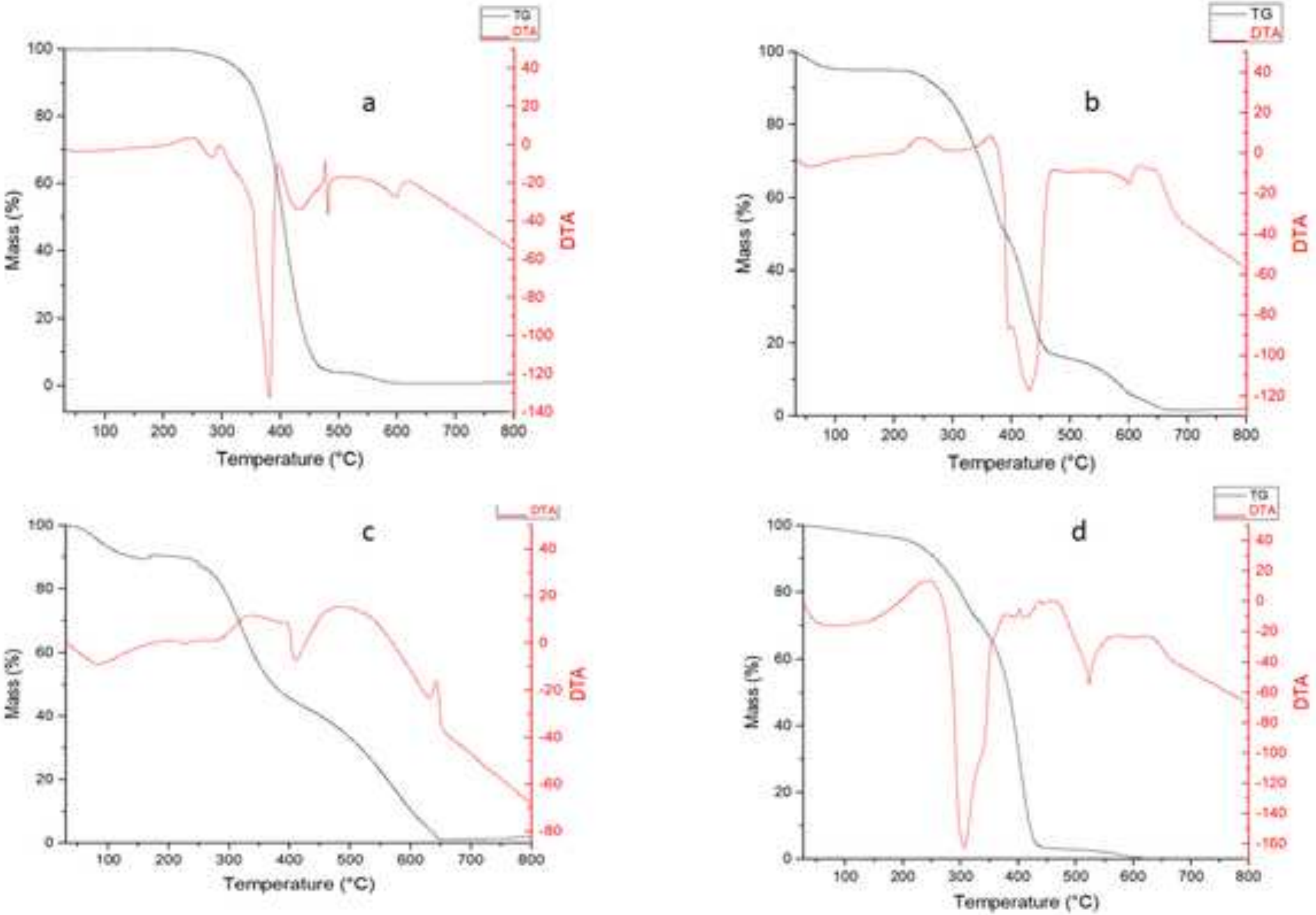

Click here to access/download;Figure;Figure 4.tif

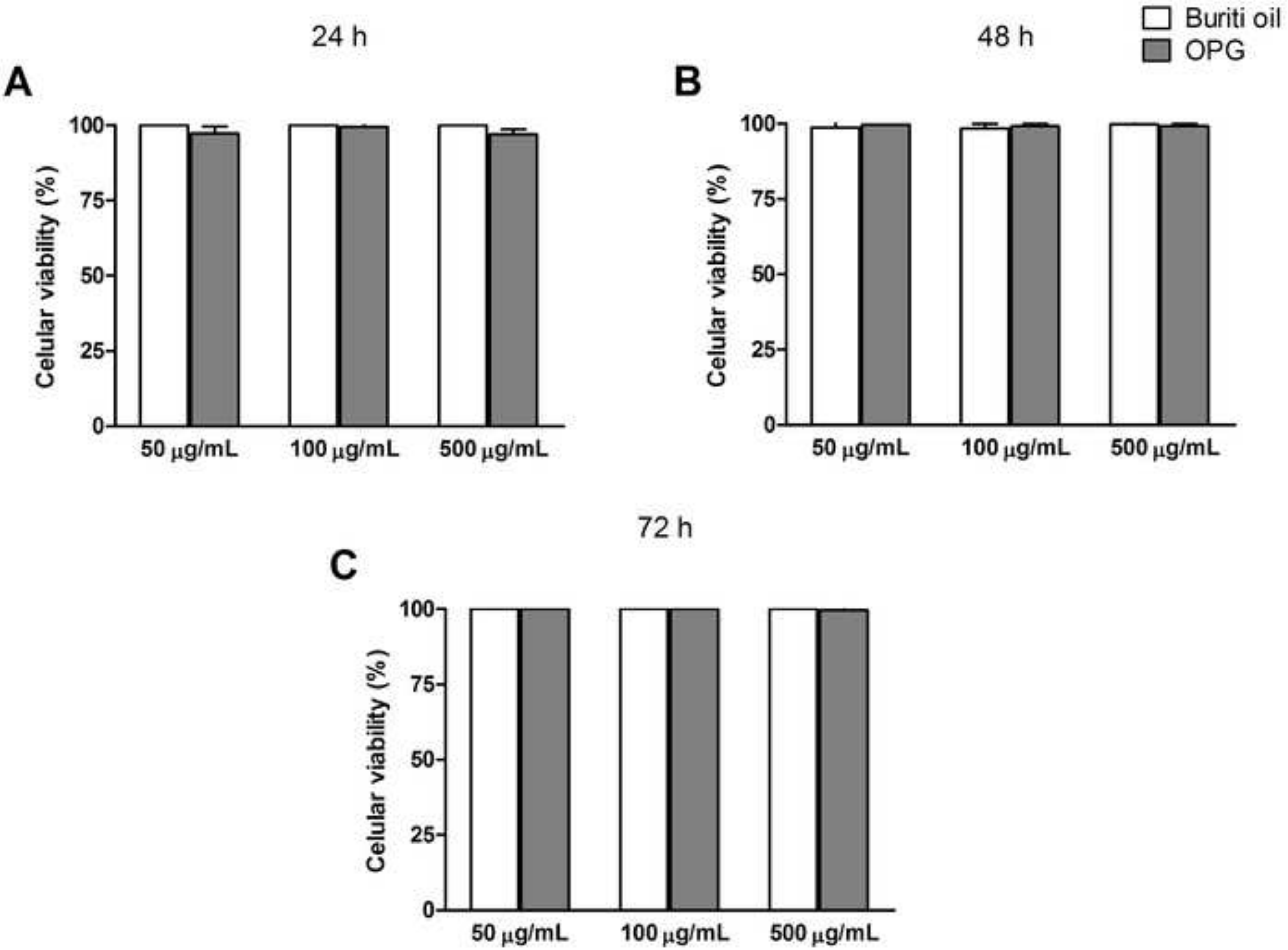

Figure

Click here to access/download;Figure;Figure 5.tif

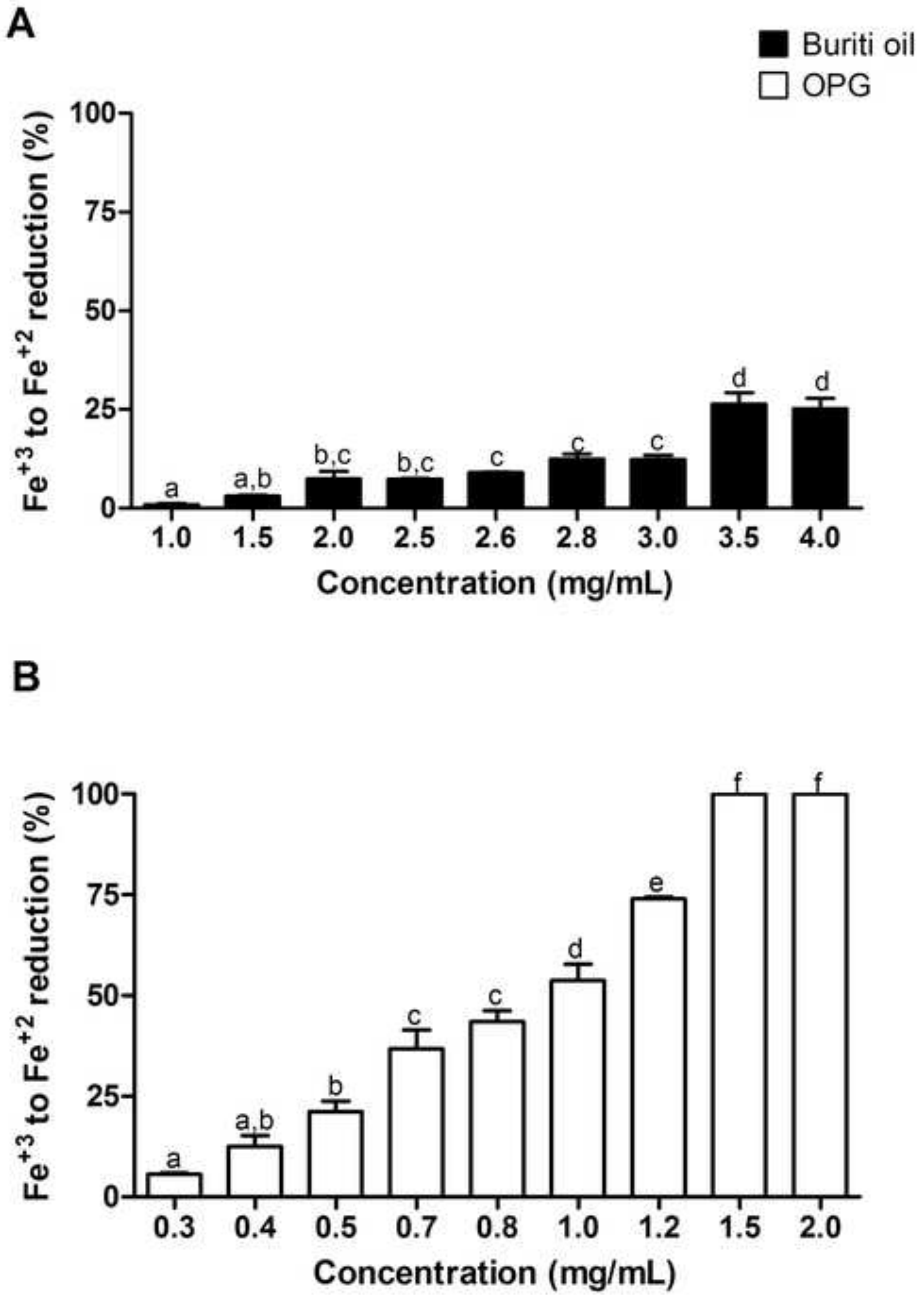

Click here to access/download;Figure;Figure 6.tif

A

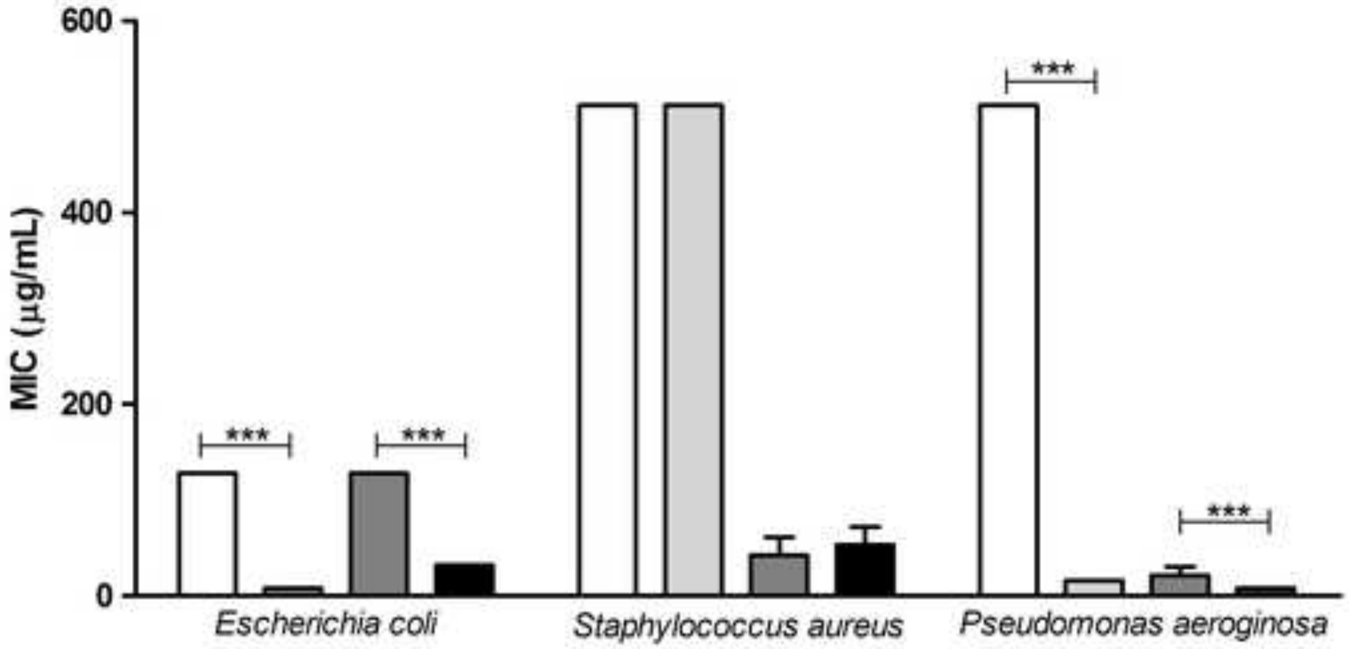

B

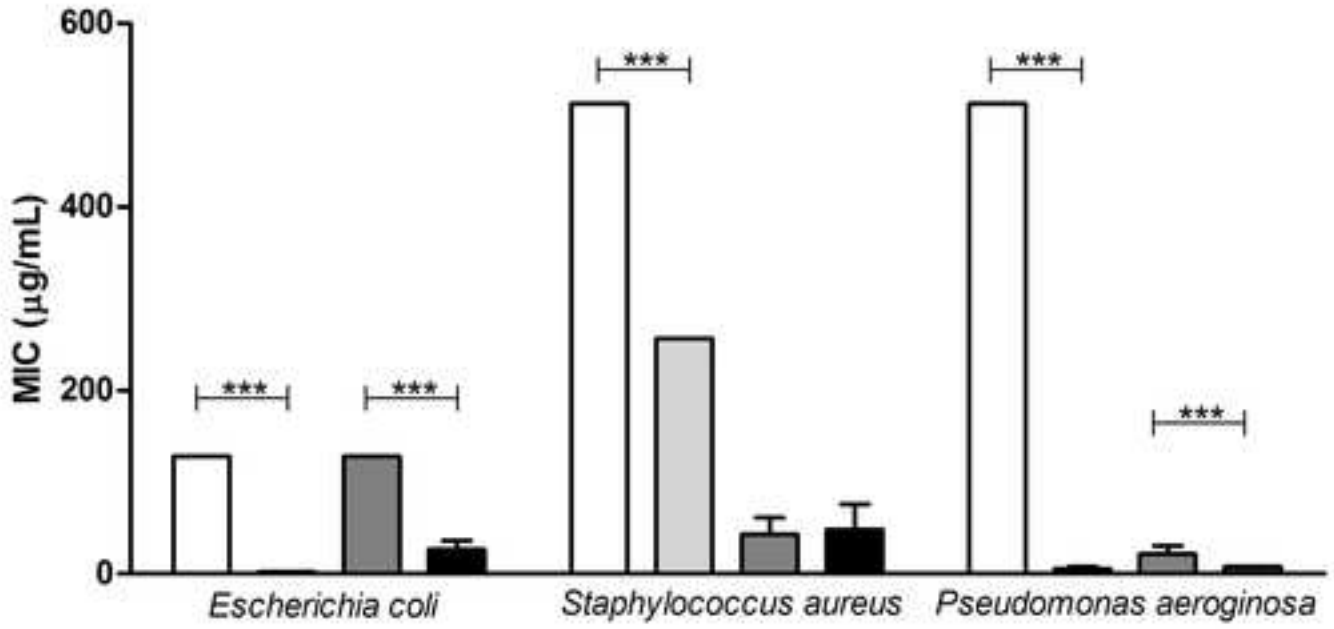

Click here to access/download
Supporting Information
Dataset.xlsx
