# Supplementary material for: Nanoencapsulation of buriti oil (Mauritia flexuosa L.f.) in porcine gelatin enhances the antioxidant potential and improves the effect on the antibiotic activity modulation
Source: PLoS One. 2022 Mar 18;17(3):e0265649. doi: 10.1371/journal.pone.0265649 (PMC8932573; doi:10.1371/journal.pone.0265649)
Supplement: Attachment — Submitted filename: PONE-D-21-38955_R1_Edited.pdf [file pone.0265649.s004.pdf]

PLOS ONE

Nanoencapsulation of buriti oil (Mauritia flexuosa L.f.) in porcine gelatin enhances the antioxidant potential and improves the effect on the antibiotic activity modulation

--Manuscript Draft--

**Manuscript Number:**	PONE-D-21-38955R1
**Article Type:**	Research Article
**Full Title:**	Nanoencapsulation of buriti oil (Mauritia flexuosa L.f.) in porcine gelatin enhances the antioxidant potential and improves the effect on the antibiotic activity modulation
**Short Title:**	Nanoencapsulation of buriti oil in porcine gelatin
**Corresponding Author:**	Cristiane Fernandes de Assis, PHD<br>Universidade Federal do Rio Grande<br>Natal, Rio Grande do Norte BRAZIL
**Keywords:**	cytotoxicity; Total Antioxidant Capacity, ABTS, Gentamicin; Norfloxacin
**Abstract:**	The present study evaluated the cytotoxicity, antioxidant potential, and antimicrobial effect on the antibiotic activity modulation of gelatin nanoparticles containing buriti oil (OPG). The cytotoxicity analysis was performed on Chinese Hamster Ovary Cells (CHO) using a MTT [3-(4,5-dimethylthiazol-2-yl)-2,5-diphenyltetrazolium bromide] test. The antioxidant potential of buriti oil and OPG was determined by total antioxidant capacity, reducing power, and the ABTS (2,2'-azinobis-3-ethylbenzothiazoline-6-sulfonic acid) test. The modulating antimicrobial activity was evaluated by determining the minimum inhibitory concentration (MIC) concentration against Escherichia coli, Pseudomonas aeruginosa, Staphylococcus aureus, gentamicin and norflaxacillin. The nanoformulation of OPG did not show a cytotoxic effect on CHO cells and had a higher antioxidant potential than free buriti oil ($p<0.05$). The combination of antibiotics with free buriti oil and OPG was more efficient in inhibiting E. coli and P. aeruginosa than isolated norfloxacillin and gentamicin ($p<0.05$). Regarding the inhibition of S. aureus , OPG in combination with norfloxacillin reduced MIC by 50%. Nanoencapsulation was a viable alternative to enhance functionality and adding commercial value to buriti oil.
**Order of Authors:**	Neyna de Santos Morais
	Thaís Souza Passos
	Gabriela Rocha Ramos
	Victória Azevedo Freire Ferreira
	Susana Margarida Gomes Moreira
	Gildácio Pereira Chaves Filho
	Ana Paula Gomes Barreto
	Pedro Ivo Palacio Leite
	Ray Silva de Almeida
	Cícera Laura Roque Paulo
	Rafael Fernandes
	Sara Sayonara da Cruz Nascimento
	Sebastião Ânderson Dantas da Silva
	Francisco Canindé Sousa Júnior
	Cristiane Fernandes de Assis, PHD
**Response to Reviewers:**	Dear Editor-in-Chief at the PLos One,<br><br>Thank you for considering our manuscript entitled "Nanoencapsulation of buriti oil in porcine gelatin enhances the antioxidant potential and improves the effect on the antibiotic activity modulation" for revision. We have carefully read the reviewers'

comments, answered all questions in this letter, and made the requested changes in the revised manuscript (highlighted in yellow). We hope the revised manuscript is now suitable for publication in PLos One.

Best regards,
Cristiane Fernandes de Assis
Department of Pharmacy
Federal University of Rio Grande do Norte
Phone: (55 84) 99152-7007
E-mail: cristianeassis@hotmail.com

Answers to reviewer #1:

The authors are carried out research for nanoformulation of buriti oil by using porcin gelatin in a scientific way, however, the need to describe the criteria was fixed in the selection of buriti fruits and formulation or production process is not well described. In the introduction part, the research gap is not clear?
Thank you for the comment. The suggestions were accepted. We made the proper changes in the results text, highlighted in yellow. The buriti oil used in the study was kindly donated by a company (Plantus® S/A) with an IBD certificate, which markets the product in Brazil and exports it to other countries. Regarding the nanoparticle synthesis process, the protocol followed was the same as that standardized by Castro et al. (1) (publication of our research group), aiming at obtaining a new batch of OPG with the same physical and chemical characteristics observed by Castro et al.(1) to continue the investigations related to the effect of nanoencapsulation on the functionality of buriti oil.

The results and discussion part have a good delivery to the information but lacking in scientific support studies. This study was already carried out by Castro et al. and Garcia et al. in terms of Characterization OPG and what is your innovation or what would you tell to the scientific community or food industrialist?

Thank you for the comment. Our research group has been developing studies with nanoencapsulated vegetable oils (1,2). The present study is a continuation of the study of Castro et al. (1) that investigated the nanoencapsulation of buriti oil using a different encapsulating agent [porcine gelatin (OPG) and the combination of sodium alginate and porcine gelatin (OAG)], to evaluate the effect on water dispersibility and antimicrobial activity. The investigation pointed to best results for OPG, the smallest particle size [51.0 (6.07) nm], chemical interaction between the materials present in the system, greater dispersibility in water [85.62% (7.82)]. It potentiated the antimicrobial activity of buriti oil in 59%, 62%, and 43% against the bacteria Pseudomonas aeruginosa, Klebsiella pneumonia, and Staphylococcus aureus, respectively. Therefore, it is of fundamental importance to deepen the knowledge regarding the effects of nanoencapsulation on the biological properties of buriti oil to assess the antioxidant potential and modulating action on antibiotics of nanoparticles based on porcine gelatin containing buriti oil (OPG).
Thus, it was necessary to ensure that the new batch of synthesized nanoformulation had the same physical and chemical characteristics observed in Castro. et al.(1) Therefore, it was necessary to carry out the characterization to confirm that the diameter, morphology, chemical interactions and incorporation efficiency were similar to the batch obtained by Castro et al. (1), ensuring that the material's functionality would be preserved and possible to continue the study. This step is of fundamental importance to guarantee the standardization of each batch of nanoformulation obtained.
The present study is innovative and unprecedented in the literature because it manages to evaluate the effect of the nanoformulation in the modulation of the antibiotic activity of known drugs, and not only of the non-encapsulated vegetable oil as presented in other studies. Interest in products developed through Nanotechnology has increased not only in the food industry but also in the pharmaceutical industry due to properties such as the high dissolution rate associated with the increased permeability of the active compound through the intestinal wall, increased physical and chemical stability of the core, the ease of handling, transport, and incorporation into the matrix of a product. In addition, to enabling water solubility, bioaccessibility and

bioavailability of lipophilic substances, preservation and enhancement of functionalities, and enabling the reduction of the concentration of the bioactive of interest necessary to achieve the desired biological effect.

Units are not well described as per author guidelines. Grammatical mistakes appeared in the manuscript and it was corrected the manuscript. Mandatory for reframing the conclusion part in the first 4 lines, the present form of conclusion is not opt for the manuscript. Figure quality is not good and mandatory need to improve the quality in all figures. Corrections were mentioned in the manuscript and need to carry out. Check the references and some of the references were missing in DOI?
Thank you for the comment. The suggestions were accepted. We made the proper changes in the results text, highlighted in yellow. We have carefully addressed the manuscript's grammar, usage, and overall readability. This English review was carried out by a native English speaker with experience in international research. We also used the software Grammarly (full version) to further check for gross mistakes. We are fully available to respond to any further questions, attending to other suggestions. Besides, the units and conclusions were adjusted based on the author's guidelines. The reference list was revised for the insertion of the DOI, and the resolution of the figures was improved.

Answers to reviewer #2:

The findings of the investigation are interesting, it uses suitable experiments to show the characteristic of the nanoencpasulated material and its action for antibiotic activity modulation. However there are some weakness in the manuscript that need to be observed. I suggest to accept the manuscript after major revision. My comments are given below.
Thank you for considering our manuscript “Nanoencapsulation of buriti oil in porcine gelatin enhances the antioxidant potential and improves the effect on the antibiotic activity modulation" for revision.

1. How encapsulation facilitates the delivery of buriti oil. Author should perform the release experiment for its practical application.
The present study is a continuation of a study previously published by our research group (Castro et al.,(1), which proposed to enable the nanoencapsulation of buriti oil, aiming to obtain a formulation with a nanoscale diameter, distribution of homogeneous size, capable of allowing the dispersibility of the oil in water, and potentiating the antimicrobial activity, that is, increasing the action using a smaller amount of buriti oil. Thus, Castro et al.(1) tested different materials (sodium alginate, porcine gelatin, the combination of both) and obtained a formulation (OPG – nanoencapsulated buriti oil in porcine gelatin) that achieved the expected results. Based on this, the main focus of the present study was to deepen the investigation of the effect of nanoencapsulation on the buriti oil functionalities, such as the antioxidant activity and modulation of the antimicrobial activity of known antibiotics and the impact on thermal stability. In addition, it was of fundamental importance to evaluate the toxicity of OPG in cells, aiming at future studies in animal models. Therefore, future studies by our research group will determine in vitro simulated digestion to obtain information on controlled release, antioxidant and anti-inflammatory effects, complementing the information with studies in eutrophic animal models and diet-induced obesity.

2. Why nanoencapsulation increased the antioxidant activity of OPG
Nanoscale diameter allows for increased contact surface for chemical interactions, increased water dispersibility, bioaccessibility, and bioavailability compared to micro or macro scale size (3). Therefore, nanoencapsulated oils are protected against oxidation of their bioactive constituents and have a higher solubility in water (1,2,4). These associated factors guarantee an increase in the antioxidant potential.

3. Why author selected porcine gelatin as wall polymer, state the prominent reason. There are other biocompatible polymers also.
Porcine gelatin is a low-cost material, widely used in the food, cosmetic and pharmaceutical industry, as a coating, binding, gelling agent in confectionery, capsules and food supplements, creams, lotions, among others, due to its structural stability, and physicochemical, functional and nutritional properties. It can act as an emulsifier in oil-in-water emulsions, promoting an increase in the physical-chemical stability of

Powered by Editorial Manager® and ProduXion Manager® from Aries Systems Corporation

polyunsaturated fats. In addition, the gelling property is of great interest for applications in the health area, as it helps in the adhesion of the particle to the mucosa to promote the controlled release.

4. Authors demonstrated herbal shotgun or synergistic multi-effect targeting in this investigation. This is very interesting finding. But, how author confirmed that the efficacy was due to synergism. In support of this statement statistical model showing the synergistic data is required.
Thank you for the comment. The suggestions were accepted. We made the proper changes in the results text, highlighted in yellow. We inserted in the article the methodology and the result (Table 5) in which we demonstrate the synergistic effect of buriti oil / OPG and antibiotics.

5. SEM micrograph is not very clear.
Thank you for the comment. The resolution of the micrographs was improved using a tool indicated by PLos One.

6. Author found the combination of antibiotics with crude buriti oil and OPG showed greater efficiency in inhibiting the action of the E. coli bacteria when compared to isolated antibiotics. Why this enhancement in efficacy was found. Give the prominent reason.

According to published studies, the antibacterial potential and modulating activity of antibiotics attributed to oils are, at least in part, associated with the fatty acids present in their composition since some fatty acids have already been shown to be able to improve antibiotic activity and inhibit the growth of bacteria (5,6). Studies suggest that oils' potential to act as antibacterial or antibiotic modulators may be associated with the detergent property of fatty acids against the amphipathic structure of bacterial cell membranes (7). This detergent property can act on the membrane (lipids and proteins), creating pores in the structure that will affect the metabolic processes of bacteria that are essential for their survival (8). Furthermore, the hydrophobic compounds in oils can increase the cell's permeability to the antibiotic, increasing the efficiency and decreasing the MIC of the antibiotic (5,9). It should be noted that these studies and possible mechanisms of action were included in the discussion of the present study, and the changes were highlighted in yellow.

7. Before adding commercial value to buriti oil, investigation about cost effectiveness is an important parameter to be considered. Author must incorporate the cost effectivity for encapsulation and how it can be commercially used?

Thank you for the comment. The suggestions were accepted. We made the proper changes in the results text, highlighted in yellow. The nanoformulation is synthesized using low-cost materials. In addition, obtaining it is scalable, and drying by lyophilization is already used in the food industry. Thus, the nanoformulation has the potential to be incorporated into foods to add nutritional value due to the presence of buriti oil and its bioactive compounds, and also potential use in food packaging to act in the inhibition of pathogenic bacteria.

8. Author presented this in line 142-145 as “After the encapsulation process, the resulting emulsion was subjected to drying by lyophilization (LioTop L101) at -57ºC and pressure of 43 μHg for further characterization, evaluation of cytotoxicity, antioxidant potential, and effect on the modulation of antibiotic activity”. Why author performed the experiments of cytotoxicity, antioxidant potential, and antibiotic modulatory activity by lyophilized sample? What is the difference, if the experiment was performed through prepared emulsion? I think, after lyophilization, there is a change of aggregation and agglomeration of particles, therefore, chances of experimental hindrance about the results.

Based on the literature (10,11) one of the advantages of the emulsification technique is to use the emulsions obtained in fluid or solid form. Therefore, powder forms can be obtained by drying the fluid emulsion by spray-drying or lyophilization. Thus, the drying of emulsions expands the range of applications in food, packaging, cosmetics, and pharmaceuticals, as it facilitates the dispersion of the material in an aqueous matrix.

Powered by Editorial Manager® and ProduXion Manager® from Aries Systems Corporation

Based on this, the emulsion obtained was dried to expand its use in several products and not just to carry out the investigations presented in this study.

9. Why Tween 20 was used as surfactant in this investigation? Why not Tween-80, based on HLB values it is more used for nanoencapsulation.

Based on published studies, such as the one by Yuan et al. (12) , from the group of polysorbates, Tween 20 stands out as the surfactant with the highest hydrophilic-lipophilic balance (HLB), approximately 16.7, being possible to promote the stabilization of the interface between oil and water, allowing to obtain particles of smaller size compared to other polysorbates.

REFERENCES

1. Castro GMMA, Passos TS, Nascimento SS da C, Medeiros I, Araújo NK, Maciel BLL, et al. Gelatin nanoparticles enable water dispersibility and potentialize the antimicrobial activity of Buriti (Mauritia flexuosa) oil. BMC Biotechnol. 2020;20(1):1–13.
2. Lira KHD da S, Passos TS, Ramalho HMM, Rodrigues D da SR, Vieira É de A, Cordeiro AMT de, et al. Whey protein isolate-gelatin nanoparticles enable the water-dispersibility and potentialize the antioxidant activity of quinoa oil (Chenopodium quinoa). PLoS One. 2020;30:1–17.
3. Tabibiazar M, Davaran S, Hashemi M, Homayonirad A, Rasoulzadeh F, Hamishehkar H, et al. Design and fabrication of a food-grade albumin-stabilized nanoemulsion. Food Hydrocoll [Internet]. 2015;44:220–8. Available from: http://dx.doi.org/10.1016/j.foodhyd.2014.09.005
4. de Campo C, dos Santos PP, Costa TMH, Paese K, Guterres SS, Rios A de O, et al. Nanoencapsulation of chia seed oil with chia mucilage (Salvia hispanica L.) as wall material: Characterization and stability evaluation. Food Chem [Internet]. 2017;234:1–9. Available from: http://dx.doi.org/10.1016/j.foodchem.2017.04.153
5. Saraiva RA, Matias EFF, Coutinho HDM, Costa JGM, Souza HHF, Fernandes CN, et al. Synergistic action between Caryocar coriaceum Wittm. fixed oil with aminoglycosides in vitro. Eur J Lipid Sci Technol. 2011;113(8):967–72.
6. Pereira YF, Costa MDS, Tintino SR, Rocha JE, Rodrigues FFG, Feitosa MK de SB, et al. Modulation of the antibiotic activity by the Mauritia flexuosa (Buriti) fixed oil against methicillin-resistant staphylococcus aureus (MRSA) and other multidrug-resistant (MDR) bacterial strains. Pathogens. 2018;7(4):1–8.
7. Chu-Kung AF, Bozzelli KN, Lockwood NA, Haseman JR, Mayo KH, Tirrell M V. Promotion of peptide antimicrobial activity by fatty acid conjugation. Bioconjug Chem. 2004;15(3):530–5.
8. Desbois AP, Smith VJ. Antibacterial free fatty acids: activities, mechanisms of action and biotechnological potential. Appl Microbiol Biotechnol [Internet]. 2010 Jan 15;85:1629+. Available from: https://link.gale.com/apps/doc/A229606459/AONE?u=capes&sid=bookmark-AONE&xid=82eb837a
9. Sousa EO de, Rodrigues FFG, Campos AR, Costa JGM da. <b>Phytochemical analysis and modulation in aminoglycosides antibiotics activity by Lantana camara L. Acta Sci Biol Sci [Internet]. 2015 Aug 6;37(2):213. Available from: http://periodicos.uem.br/ojs/index.php/ActaSciBiolSci/article/view/22877
10. Ferreira CD, Nunes IL. Oil nanoencapsulation: development, application, and incorporation into the food market. Nanoscale Res Lett. 2019;14.
11. Comunian TA, Silva MP, Moraes ICF, Favaro-Trindade CS. Reducing carotenoid loss during storage by co-encapsulation of pequi and buriti oils in oil-in-water emulsions followed by freeze-drying: Use of heated and unheated whey protein isolates as emulsifiers. Food Res Int [Internet]. 2020;130:108901. Available from: http://www.sciencedirect.com/science/article/pii/S0963996919307872
12. Yuan Y, Gao Y, Zhao J, Mao L. Characterization and stability evaluation of β-carotene nanoemulsions prepared by high pressure homogenization under various emulsifying conditions. Food Res Int [Internet]. 2008;41(1):61–8. Available from: http://www.sciencedirect.com/science/article/pii/S0963996907001627

Additional Information:	
**Question**	**Response**
**Financial Disclosure**	This study was partly financed by the

Powered by Editorial Manager® and ProduXion Manager® from Aries Systems Corporation

Enter a financial disclosure statement that describes the sources of funding for the work included in this submission. Review the submission guidelines for detailed requirements. View published research articles from *PLOS ONE* for specific examples.<br><br>This statement is required for submission and **will appear in the published article** if the submission is accepted. Please make sure it is accurate.<br><br>**Unfunded studies**<br>Enter: *The author(s) received no specific funding for this work.*<br><br>**Funded studies**<br>Enter a statement with the following details:<br>• Initials of the authors who received each award<br>• Grant numbers awarded to each author<br>• The full name of each funder<br>• URL of each funder website<br>• Did the sponsors or funders play any role in the study design, data collection and analysis, decision to publish, or preparation of the manuscript?<br>• **NO** - Include this sentence at the end of your statement: *The funders had no role in study design, data collection and analysis, decision to publish, or preparation of the manuscript.*<br>• **YES** - Specify the role(s) played.<br><br>* typeset	Coordenação de Aperfeiçoamento de Pessoal de Nível Superior - Brasil (CAPES) - Finance Code 001.
**Competing Interests**<br><br>Use the instructions below to enter a competing interest statement for this submission. On behalf of all authors, disclose any competing interests that could be perceived to bias this work—acknowledging all financial support and any other relevant financial or non-financial competing interests.<br><br>This statement is required for submission and **will appear in the published article** if	The authors have declared that no competing interests exist.

Powered by Editorial Manager® and ProduXion Manager® from Aries Systems Corporation

the submission is accepted. Please make sure it is accurate and that any funding sources listed in your Funding Information later in the submission form are also declared in your Financial Disclosure statement.<br><br>View published research articles from *PLOS ONE* for specific examples.<br><br>**NO authors have competing interests**<br><br>Enter: *The authors have declared that no competing interests exist.*<br><br>**Authors with competing interests**<br><br>Enter competing interest details beginning with this statement:<br><br>*I have read the journal's policy and the authors of this manuscript have the following competing interests: [insert competing interests here]*<br><br>* typeset	
**Ethics Statement**<br><br>Enter an ethics statement for this submission. This statement is required if the study involved:<br><br>• Human participants<br>• Human specimens or tissue<br>• Vertebrate animals or cephalopods<br>• Vertebrate embryos or tissues<br>• Field research<br><br>Write "N/A" if the submission does not require an ethics statement.<br><br>General guidance is provided below. Consult the submission guidelines for detailed instructions. **Make sure that all information entered here is included in the Methods section of the manuscript.**	N/A

**Format for specific study types**<br><br>**Human Subject Research (involving human participants and/or tissue)**<br>• Give the name of the institutional review board or ethics committee that approved the study<br>• Include the approval number and/or a statement indicating approval of this research<br>• Indicate the form of consent obtained (written/oral) or the reason that consent was not obtained (e.g. the data were analyzed anonymously)<br><br>**Animal Research (involving vertebrate animals, embryos or tissues)**<br>• Provide the name of the Institutional Animal Care and Use Committee (IACUC) or other relevant ethics board that reviewed the study protocol, and indicate whether they approved this research or granted a formal waiver of ethical approval<br>• Include an approval number if one was obtained<br>• If the study involved *non-human primates*, add *additional details* about animal welfare and steps taken to ameliorate suffering<br>• If anesthesia, euthanasia, or any kind of animal sacrifice is part of the study, include briefly which substances and/or methods were applied<br><br>**Field Research**<br><br>Include the following details if this study involves the collection of plant, animal, or other materials from a natural setting:<br>• Field permit number<br>• Name of the institution or relevant body that granted permission	
**Data Availability**<br><br>Authors are required to make all data underlying the findings described fully available, without restriction, and from the time of publication. PLOS allows rare exceptions to address legal and ethical concerns. See the PLOS Data Policy and FAQ for detailed information.	Yes - all data are fully available without restriction

A Data Availability Statement describing where the data can be found is required at submission. Your answers to this question constitute the Data Availability Statement and **will be published in the article**, if accepted.<br><br>**Important:** Stating 'data available on request from the author' is not sufficient. If your data are only available upon request, select 'No' for the first question and explain your exceptional situation in the text box.<br><br>Do the authors confirm that all data underlying the findings described in their manuscript are fully available without restriction?	
**Describe where the data may be found in full sentences. If you are copying our sample text, replace any instances of XXX with the appropriate details.**<br><br>• If the data are **held or will be held in a public repository**, include URLs, accession numbers or DOIs. If this information will only be available after acceptance, indicate this by ticking the box below. For example: *All XXX files are available from the XXX database (accession number(s) XXX, XXX.*).<br>• If the data are all contained **within the manuscript and/or Supporting Information files**, enter the following: *All relevant data are within the manuscript and its Supporting Information files.*<br>• If neither of these applies but you are able to provide **details of access elsewhere**, with or without limitations, please do so. For example:<br><br>*Data cannot be shared publicly because of [XXX]. Data are available from the XXX Institutional Data Access / Ethics Committee (contact via XXX) for researchers who meet the criteria for access to confidential data.*<br><br>*The data underlying the results presented in the study are available from (include the name of the third party*	All relevant data are within the manuscript and its Supporting Information files.

and contact information or URL).<br>• This text is appropriate if the data are owned by a third party and authors do not have permission to share the data.<br><br>* typeset	
Additional data availability information:	Tick here if the URLs/accession numbers/DOIs will be available only after acceptance of the manuscript for publication so that we can ensure their inclusion before publication.

Powered by Editorial Manager® and ProduXion Manager® from Aries Systems Corporation

Manuscript
Click here to access/download;Manuscript;Revised manuscript.docx

Nanoencapsulation of buriti oil (*Mauritia flexuosa L.f.*) in porcine gelatin enhances the antioxidant potential and improves the effect on the antibiotic activity modulation

[1]Neyna de Santos Morais, [2]Thaís de Souza Passos, [3]Gabriela Rocha Ramos, [3]Victoria Azevedo Freire Ferreira, [4]Susana Margarida Gomes Moreira, [4]Gildácio Pereira Chaves Filho, [3]Ana Paula Gomes Barreto, [5]Pedro Ivo Palacio Leite, [5]Ray Silva de Almeida, [5]Cícera Laura Roque Paula, [6]Rafael Fernandes, [1]Sebastião Ânderson Dantas da Silva, [7]Sara Sayonara da Cruz Nascimento, [1,3]Francisco Canindé de Sousa Júnioar,[1,3]*Cristiane Fernandes de Assis

[1]Nutrition Postgraduate Program, Center for Health Sciences, Federal University of Rio Grande do Norte, Natal, RN, Brazil.

[2]Department of Nutrition, Center for Health Sciences, Federal University of Rio Grande do Norte, Natal, RN, Brazil.

[3]Department of Pharmacy, Center for Health Sciences, Federal University of Rio Grande do Norte, Natal, RN, Brazil.

[4]Department of Cellular and Molecular Biology, Biosciences Center, Federal University of Rio Grande do Norte, Natal, RN, Brazil.

[5]Department of Chemical Biology, Regional University of Cariri, Crato, CE, Brasil.

[6]Chemical Intitute, Federal University of Rio Grande do Norte, Natal, RN, Brazil.

[7]Postgraduate Program in Biotechnology - RENORBIO, Biosciences Center, Federal University of Rio Grande do Norte, Natal, RN, Brazil.

*Corresponding author: cristianeassis@hotmail.com

Abstract

The present study evaluated the cytotoxicity, antioxidant potential, and antimicrobial effect on the antibiotic activity modulation of gelatin nanoparticles containing buriti oil (OPG). The cytotoxicity analysis was performed on Chinese Hamster Ovary Cells (CHO) using a MTT [3-(4,5-dimethylthiazol-2-yl)-2,5-diphenyltetrazolium bromide] test. The antioxidant potential of buriti oil and OPG was determined by total antioxidant capacity, reducing power, and the ABTS (*2,2'-azinobis-3-ethylbenzothiazoline-6-sulfonic acid)* test. The modulating antimicrobial activity was evaluated by determining the minimum inhibitory concentration (MIC) concentration against *Escherichia coli, Pseudomonas aeruginosa, Staphylococcus aureus,* gentamicin and norflaxacillin. The nanoformulation of OPG did not show a cytotoxic effect on CHO cells and had a higher antioxidant potential than free buriti oil ($p<0.05$). The combination of antibiotics with free buriti oil and OPG was more efficient in inhibiting *E. coli* and *P. aeruginosa* than isolated norfloxacillin and gentamicin ($p<0.05$). Regarding the inhibition of *S. aureus*, OPG in combination with norfloxacillin reduced MIC by 50%. Nanoencapsulation was a viable alternative to enhance functionality and adding commercial value to buriti oil.

Introduction

Mauritia palm or buriti (*Mauritia flexuosa L.f.*) is an abundant palm tree in South America, predominant in extensive areas of Brazil and native to areas of the Amazon Forest and Cerrado biome(1). Buriti fruit is considered as a functional food due to its high levels of carotenoids. The pulp is regarded as one of the primary sources of carotenoids found in Brazilian flora diversity, with emphasis on β carotene, which has provitamin activity A(2). In addition to containing minerals (calcium, zinc, sodium), fibers, proteins, and unsaturated fatty acids(3).

Buriti oil can be obtained from the fruit by extracting the pulp and/or pulp and peel by conventional methods (mechanical and chemical extraction)(4–6), capable of influencing the product characteristics. It is of great interest in the cosmetic and food product industries because it has a wide and varied application due to its chemical composition(7).

The oil contains vitamin E considered an important antioxidant capable of interrupting the action of free radicals and protecting the cytoplasmic membranes from oxidation, reducing pre-cancerous lesions(8–10). In addition, it also has unsaturated fatty acids, which are associated with reduced levels of triglycerides and total cholesterol, consequently constituting a protective factor for cardiovascular diseases(11) and a protective effect on platelet activation and thrombosis(12). It also has ascorbic acid (vitamin C), which helps form bone and teeth and acts on the immune system, preventing flu, diabetes, and scurvy(13).

Furthermore, vegetable oils have a potential effect against microorganisms such as bacteria(5). Thus, the search for new compounds with antibacterial activity has been the objective of some research to solve the problem associated with bacterial resistance, which has become common in recent decades, increasing the concern of health authorities(14,15). In this context, natural products have been an essential tool for being a source of new compounds with antibacterial properties, which can modulate and increase the activity and efficiency of conventional antibiotics or be resistance modifying agents(15).

Thus, it is well-known that buriti oil has numerous biological properties(16). However, bioactive compounds are susceptible to degradation due to sensitivity to processing and storage factors such as temperature, pH, presence of light, and oxygen (17,18). The oil's oxidation process causes unpleasant tastes, loses nutrients, and produces many toxic compounds

(hydroperoxides and aldehydes) that cause mutation, aging, stroke, emphysema, heart disease, and cancer(19). Another technological obstacle associated with the use of buriti oil in foods is associated with its lipophilic nature, which makes it challenging to apply in products with aqueous matrices, limiting the development of new products(20).

[illegible] this context, nanoencapsulation is a strategy that can be used to protect substances against environmental factors (heat, light, oxygen, humidity), improve stability, acceptability, and handling conditions, restrict contact with other components and control the release and delivery of bioactives to the target site. In addition, reducing the size to the nanometric scale (< 100 nm) facilitates dispersion of lipophilic substances in an aqueous matrix due to the increase in the contact surface. It can also preserve or enhance the biological properties of bioactive compounds(19–22). Thus, nanoencapsulation can increase the antioxidant and antimicrobial potential, among other functionalities, reducing the concentration of the bioactive of interest necessary to achieve the desired effect (23).

Considering the properties present in vegetable oils, especially buriti oil, and the current needs of the food and pharmaceutical industry, our research group has been developing studies with nanoencapsulated vegetable oils (20,22). Castro et al. (20) investigated the nanoencapsulation of buriti oil using different encapsulating agents, namely porcine gelatin (OPG) and the combination of sodium alginate and porcine gelatin (OAG), to evaluate the effect on water dispersibility and antimicrobial activity. The results showed that OPG presented the smallest particle size [51.0 (6.07) nm] and the best chemical interaction between the materials present in the system. As a result, OPG showed greater dispersibility in water [85.62% (7.82)] and potentiated the antimicrobial activity of buriti oil in 59%, 62%, and 43% against the bacteria *Pseudomonas aeruginosa*, *Klebsiella pneumonia,* and *Staphylococcus aureus*, respectively.

Therefore, due to the potential of buriti oil both from an economic and biotechnological point of view and the results obtained by Castro et al. (20), it is of fundamental importance to deepen the knowledge regarding the effects of nanoencapsulation on the biological properties of buriti oil. The resistance of microorganisms to antibiotics has increased interest in research that seeks new drugs with antimicrobial potential or that act by modulating the activity of existing antibiotics. This study aimed to assess the cytotoxicity, antioxidant potential, and modulating action on antibiotics of nanoparticles based on porcine gelatin containing buriti oil (OPG).

Materials and Methods

Materials

Buriti oil from the *Mauritia flexuosa* species was supplied by the Plantus® S.A. cosmetics company (Nísia Floresta, in the Rio Grande do Norte, Brazil) with an IBD certificate (Agricultural and Food Inspections and Certifications). The samples were transported under protection from light and refrigerated to the Bromatology Laboratory of the Department of Pharmacy at the Federal University of Rio Grande do Norte (UFRN), where they were stored at 4 ºC.

Porcine gelatin (Type A), the surfactant Tween 20, 2,2’-azinobis (3-ethylbenzothiazoline-6-sulfonic acid) (ABTS), and resazurin obtained from Sigma-Aldrich®.

Microorganisms

Multiresistant strains of *Staphylococcus aureus* 10, *Pseudomonas aeruginosa* 24, and *Escherichia coli* 06 were supplied by the Laboratory of Microbiology and Molecular Biology of the Regional University of Cariri - URCA (Crato - CE, Brazil). The Chinese hamster ovary cell line (CHO-K1 cells) was purchased from ATCC® CCL-61™ (American Type Culture Collection), and Dulbecco’s Modified Eagle Culture Medium (DMEM; Gibco, Gaithersbug, MD, USA) was used, while 3-4,5-Dimethyl-thiazol-2-yl-2,5-diphenyltetrazolium bromide (MTT) was purchased from Invitrogen (MTT; Invitrogen, Oregon, USA).

Methods

Obtaining the nanoformulation containing buriti oil

The nanoformulation based on porcine gelatine containing buriti oil (OPG) was obtained through the technique of O/W emulsification followed by dispersion of a solution containing encapsulating agent in the obtained emulsion based on Medeiros et al.(24) with modifications proposed by Castro et al.(20). Porcine gelatin (Sigma®) and Tween 20 (Sigma®) were used as encapsulating agent and surfactant, respectively. After the encapsulation process, the resulting emulsion was subjected to drying by lyophilization (LioTop L101) at -57 ºC and pressure of 43 μHg.

Characterization of the obtained nanoparticles

The new batch of OPG obtained for this study was characterized to determine its morphology, particle diameter, and chemical interactions by Scanning Electron Microscopy, Dynamic Light Scattering, and Fourier Transform Infrared Spectroscopy, respectively, according to Castro et al.(20). Furthermore, it was also evaluated concerning the distribution of charges on the surface of the particles and in terms of thermal resistance by Zeta Potential, Thermogravimetry, and Differential Thermal Analysis, respectively.

Scanning electron microscopy (SE[illegible]

OPG was suspended in acetone, and the dispersion was dropped onto silicon plates attached to stubs using carbon tape. It was subsequently analyzed at different magnifications in high vacuum at 2-3 kV and without metallization using a FEG-SEM ZEISS microscope (AURIG[illegible]

Dynamic light scattering (DLS[illegible]

The nanoformulation was subjected to crosslinking with glutaraldehyde to measure the diameter of the particles, according to Castro et al.(20), to promote particle deagglomeration. It was subsequently dispersed using an ultrasonic bath in DMSO (dimethylsulfoxide [illegible]mg.mL^{-1}). Measurements were performed in triplicate (60 s each) using only 2 mL of suspension. Data were analyzed using the NANO-flex Control 0.9.7 software program. The entire experiment was carried out in triplicate. The data obtained referring to the mean and standard deviation were plotted in the Origin® 8 program to obtain the histogram with the particle size distribution.

Fourier transform infrared spectroscopy (FTIR)

Buriti oil, porcine gelatin, Tween 20, and OPG were homogenized separately with potassium bromide (KBr). Then they were macerated and pressed to obtain pellets. The spectra were recorded in transmittance with the mid-infrared region (400 to 4000 cm^{-1}). A Shimadzu spectrometer (FTIR-8400S, IRAFFINITY-1 series, IR SOLUTION version 1.60 software) was used with a scan number of 32 and resolution of 4 cm^{-1}.

Zeta Potential

The Zeta Potential measurements of the OPG nanoformulation were determined using a STABINO II Particle charge Titration device (Colloid Metrix). First, 10 mg of the sample was

diluted in 10 mL of ultrapure water (≥ 18 MΩ cm^{-1}) and then transferred to the cylindrical Teflon cell. The zeta potential measurement with pH variation (similar to a titration) consisted of individually adding aliquots (10 μL) of a strong acid (HCl - 0.1 M) or a strong base (NaOH – 0.025 M).

Thermogravimetry (tg) and differential thermal analysis (DTA)

We employed thermoanalytical analyzes to evaluate the buriti oil, Tween 20, porcine gelatin, and OPG nanoformulation. Thus, from 6 to 7 mg of each material were evaluated in a Shimadzu® DTG-60 thermal analyzer with a heating rate of 10 °C.min^{-1} in the temperature range of 30 °C to 800 °C in a nitrogen atmosphere with a flow of 50 mL.min^{-1}.

It is noteworthy that the variations in the residual mass of the materials concerning the variation in time and temperature were provided by the equipment. The Origin Pro Graphing & Analysis version 9.8.0.200 software program from OriginLab® was used to build the TG and DTA graphs.

Incorporation efficiency (%)

The incorporation efficiency was determined based on El-Messery et al.(25) with modifications. 15 mL of hexane in 1.5 g of the nanoformulation were added under agitation in a TE-421 rotary incubator (Tecnal®) for 2 min, and then the mixture was filtered on Whatman No.1 filter paper.

Next, the content retained on the filter paper was washed twice with 20 mL of hexane. The liquid phase was collected, and the hexane was removed in an incubator at 60 °C for 48 h. The remaining extract represents the unencapsulated oil present on the surface of the encapsulates. The amount of nanoencapsulated oil was obtained through the difference between the initial amount of oil used to promote the nanoencapsulation process and the free oil present on the surface of the nanoparticles after the hexane evaporates, as described below (Equation 1). The average amount of OPG obtained from the triplicate produced and measured with the aid of an analytical balance was considered (Edutec, EEQ9003F-B).

$$EO\ (g) = OUE - RO\ Equation\ (1)$$

EO: Quantity of encapsulated oil; OUE: Weight of oil used in encapsulation; RO: Weight of remaining oil.

The encapsulation incorporation was calculated using Equation 2, described below according to El-Messery et al.(25), considering the average amount of OPG obtained from the triplicate produced and measured using an analytical scale (Edutec, EEQ9003F-B).

$$EI\ (\%) = \left[\frac{EO}{IO}\right] x100\ Equation\ (2)$$

EI: Encapsulation incorporation; EO: Amount of encapsulated oil; IO: Initial oil quantity

Cytotoxicity evaluation

MTT assay

Chinese hamster ovary (CHO) cells were seeded in 96-well plates at a density of $2x10^3$ cells.mL^{-1} and cultured in α-MEM medium supplemented with 10% FCS (fetal bovine serum), 1% antibiotic and antimycotic solution, and 1% glutamine (basal medium).

At the end of 24 hours, the medium was changed with the medium containing the buriti oil and OPG samples at concentrations 50, 100, and 500 µg.mL^{-1}, and cytotoxicity was evaluated after 24, 48, and 72 hours. Cells maintained in the basal medium were used as a negative control. At the end of each time, 1 mg.mL^{-1} of MTT solubilized in PBS buffer was added, the supernatant was aspirated after incubation at 37 °C for 4 hours, and the formazan crystals solubilized with 200 µl of DMSO. Absorbance was measured at 570 nm in a plate reader (BioTek, µQuant model), and cell viability was calculated according to Equation 3 described below.

$$MTT\ reduction(\%) = \frac{Abs\ treatment}{Abs\ negative\ control}\ x\ 100\ Equation\ (3)$$

Antioxidant activity

Total antioxidant capacity (TAC)

The method proposed by Prieto et al(26). was used to determine TAC (Total antioxidant capacity). In each test tube, 100 µL of 40 mM sulfuric acid-ammonium molybdate, 280 mM sodium phosphate, and 100 µL of the sample solutions were added at a concentration of 2 mg.mL^{-1} and then 700 µL of distilled water in the test tubes.

Then, the tubes were shaken and incubated in a water bath (QUIMIS, Mod. Q334M-28) at 90 ºC for 90 minutes to subsequently take the absorbance reading by spectrophotometry (Biospectrum, Mod. SP-220) at 695 nm. The experiment was carried out in triplicate using solutions containing the encapsulating agents as a control. Antioxidant activity was expressed in milligrams of ascorbic acid per gram of sample (mg AA.g of sample^{-1}). The standard curve was constructed using different ascorbic acid concentrations (25 -250 mg.g^{-1}).

Reducing power test

The reducing power of the samples was quantified as described by Wang et al.(27). First, 4 mL of a reaction mixture containing different buriti oil sample (1-50 mg.mL^{-1}) and OPG (3-20 mg.mL^{-1}) concentrations in phosphate buffer (0.2 M, pH 6.6) were incubated with potassium ferrocyanide (1% m/v) at 50 ºC for 20 minutes. The reaction was terminated by a TCA solution (10% m/v). The solution was then mixed with distilled water and iron chloride (0.1% m/v), and the absorbance was measured at 700 nm. The control was carried out with the encapsulating agent. The result was expressed in the reduction of Fe^{+3} to Fe^{+2} (%).

Antioxidant activity by 2,2'-azinobis-3-ethylbenzothiazoline-6-sulfonic acid (ABTS)

The ability to scavenge the 2,2'-azinobis-3-ethylbenzothiazoline-6-sulfonic acid (ABTS•) radical cation was determined by adapting the methodology described by Rufino et al.(28). The ABTS• solution was prepared by adding ABTS• (7 mM) and potassium persulfate (2.45 mM) and incubated at room temperature in the dark for 16 h. Next, the ABTS solution (1 mL) was diluted in DMSO to obtain an absorbance of 0.8, at a wavelength of 734 nm (29,30). Finally, 200 µL of this ABTS• radical solution was carefully added to the microplate well together with 40 µL of the evaluated samples.

The control was prepared to contain 200 µL of ABTS and 40 µL of DMSO. The absorbance reading at 734 nm was performed in a microplate reader (Loccus – Model LMR FLEX). After reading the absorbances, the antioxidant activity (AA) percentage was estimated using Equation 4 described below.

$$ABTS\ radical\ inhibition = 100\ X\ \frac{Abs\ control - Abs\ sample}{Abs\ control}\quad Equação\ (4)$$

The linear regression curve was determined to calculate the concentration required for the samples (crude oil and OPG) to inhibit 50% of the ABTS radical (IC 50).

Determination of the modulating antimicrobial activity of antibiotics

The antimicrobial activity test was performed by determining the Minimum Inhibitory Concentration (MIC) of antibiotics (norfloxacin and gentamycin) and modulating activity using buriti oil and OPG (dilution of in DMSO, in the concentration of 1024 µg.mL^{-1}).

The bacteria used were multiresistant *Pseudomonas auriginosa* 24, *Staphylococcus aureus* 10, and *Escherichia coli* 06 strains collected in exams provided by the Regional University of Cariri (Juazeiro do Norte/CE). The origin and resistance profile of these strains were described by Bezerra et al.(31).

Bacteria cultures of 10^{7} CFU.mL^{-1} kept in agar were subcultured in brain-heart broth (Brain Heart Infusion - BHI) at 37 ºC for 24 h. Then, the distribution medium was serially prepared at 1:10, and 100 µL were added into Eppendorfs of BHI medium in 900 µL of inoculum. This distribution medium was transferred to the microplate, 100 µL in each well. Norfloxacin and gentamycin antibiotics were added to the test, and their inhibitory effects were investigated in their combinations with buriti oil and OPG. Both concentrations of antibiotics, crude buriti oil and OPG, were fixed at 1024 µg.mL^{-1} with a dilution of 100 µL in octuplicate. Controls were included in the trials, namely the medium and the inoculum, the porcine gelatin, and the Triton X-100. The filled plates were incubated at 35 (±2)ºC for 24 hours(32).

Next, an indicator solution of sodium resazurin was prepared in sterile distilled water at a concentration of 0.01% (m/v) to evidence the MIC of the samples. After incubation, 20 µL of the indicator solution was added to each well and the plates underwent an incubation period of 1 hour at room temperature. A change from blue to pink color due to the reduction of resazurin indicated bacterial growth at 37 ºC (32). MIC was defined as the lowest concentration capable of inhibiting microbial growth, as evidenced by the unaltered blue color.

According to the equations 4,5,6 below, the fractional inhibitory concentration index (FICi) measurement was determined to demonstrate the synergistic effect. The FICi index was interpreted according to the European Committee for Antimicrobial Susceptibility Testing (30), considering the synergistic effect FICi ≤ 0.5 (33).

$$FIC\ buriti\ oil\ or\ OPG = \frac{MIC\ of\ buriti\ oil\ \ or\ OPG\ in\ combination}{MIC\ \ of\ buriti\ oil}\ Equation\ (4)$$

$$FIC\ antibiotic = \frac{MIC\ of\ antibiotic\ in\ combination}{MIC\ of\ antibiotic}\ Equation\ (5)$$

$$FIC\ index\ (FICi) = (FIC\ oil\ buriti\ or\ OPG + FIC\ antibiotic)\ Equation\ (6)$$

Statistical analysis

Statistical analysis was performed using Graph Pad Prism version 5.0 software. Results were expressed as mean and standard deviation. First, the data obtained for the analyzes related to antioxidant potential and antimicrobial activity and modulation of antibiotic action were evaluated for normality using the Shapiro-Wilk test. Data were evaluated using the Student's t-test to determine the antioxidant potential through CAT and ABTS tests and assess antimicrobial activity and cytotoxicity. An analysis of variance and Tukey's post-test were used for the reducing power. A value of $p<0.05$ was considered statistically significant in all cases.

Results and Discussion

Characterization of porcine gelatin-based nanoparticles containing buriti oil (OPG)

The micrographs obtained through Scanning Electron Microscopy (SEM) analysis (Fig 1A) showed particles with a spherical shape, smooth surface without cracks and physical size at the nanometer scale (<100 nm), demonstrating good protection of the core through the encapsulation. The results obtained by Castro et al.(20) for buriti oil nanoencapsulated in porcine gelatin (OPG) were similar. Lira et al.(22) showed the same characteristics observed for shape and surface in all formulations containing quinoa oil.

Figure 1. Characterization of buriti oil nanoencapsulated in porcine gelatin. A. Micrograph obtained by Scanning Electron Microscopy. B. Dynamic light scattering. C. Fourier Transform Infrared Spectroscopy of nanoencapsulated buriti oil (a), buriti oil (b), Tween 20 (c), porcine gelatin (d).

The data obtained for particle diameter using DLS (Fig 1B) showed mean diameter and polydispersion index for OPG equal 72.0(0.68) and 0.371(0.0251), respectively. Thus, it is possible to state that OPG presented unimodal size distribution and reinforced the physical

diameter observed by SEM, confirming that it is a nanoparticle. The nanometric particle size enables the food industry to improve the bioavailability of poorly soluble substances, such as lipids and natural antioxidants(34).

Castro et al.(20) found values of 51.00 (6.07) nm and 0.40 (0.05) for particle size and polydispersion index for OPG, respectively, constituting values close to those found in this work. Particles with a large diameter and size distribution can affect the texture and compromise the incorporated bioactive compounds, which did not occur in this study. The polydispersion index of less than 1 indicates homogeneity in the analyzed particle(20,35). Thus, a particle that presents itself more homogeneously is inserted in a product more efficiently, not affecting the texture, therefore being positive for consumer use(35,36).

Fig 1C shows the FTIR spectra obtained for nanoencapsulated buriti oil, buriti oil, Tween 20, porcine gelatin. Hydrocarbon groups were detected in the buriti oil spectrum (Fig 1C, line b) by vibrational bands in the range of 2924 - 2852 cm^{-1} (C-H), in addition to bands in the region of 3007 cm^{-1} and 1462 cm^{-1}, which respectively characterize the –OH and $–CH_3$ groups, meaning that they reflect the strong constitution of this oil described in the literature as a source ingredient of carotenoids [16]. The stretching of the vibrational band at 1743 cm^{-1} indicates the presence of double bonds in the oil chain (C = C; C = O), which shows the content of unsaturated fatty acids(37).

The vibrational band at 1651 cm^{-1} in the porcine gelatin spectrum (Fig 1C, line d) reflects the presence of the C=O bond (amide I), according to Silverstein & Webster(37). Another band is observed at 1529 cm-1, indicating amide (N-H)(38).

The spectrum of the Tween 20 surfactant (Fig 1C, line c) presented vibrations at 2920 cm^{-1} and 2864 cm^{-1} referring to stretching of the asymmetric and symmetrical methylene vibrations; at 1735 cm^{-1}, referring to the C=O connection; and at 1095 cm^{-1}, referring to the vibration stretching of $–CH_2-O-CH_2-$(37).

When observing the spectrum obtained for OPG (Fig 1C, line a), it can be seen that there was an interaction between porcine gelatin, buriti oil, and Tween 20 surfactant due to the attenuation and/or displacement of the vibrational bands that characterize the presence of the buriti oil (3007, 2924, 2852 and 1743 cm^{-1}), indicating protection of the oil in the obtained particles. The formation of new bands was also observed, which were not observed in the spectra of the

raw materials (1556, 1348, and 1097 cm^{-1}), which may indicate hydrophobic interactions between buriti oil and the non-polar amino acids of porcine gelatin. Castro et al.(20) also observed chemical interactions between OPG constituents, meaning buriti oil with porcine gelatin.

The encapsulation efficiency determines the oil content (%) that was successfully encapsulated inside the particles, also being an indicator of free oil present on the surface of the particles(39). The nanoformulation yield was 13.65g (1.14), and the result of the encapsulation efficiency was 89.56% (1.14). According to the literature is considered high efficiency, or when ≥ 80%(40,41).

Thus, this analysis confirmed the results obtained through the FTIR, which indicated a chemical interaction between gelatin, buriti oil, and Tween 20, which managed to provide greater oil retention. This result can be explained by the fact that proteins have physicochemical properties which favor forming and stabilizing emulsions than carbohydrates, thus allowing greater oil retention in the particles(20,42).

It was necessary to ensure that the new batch of synthesized nanoformulation had the same physical and chemical characteristics observed in Castro. et al. (2020). Therefore, it was necessary to carry out the characterization to confirm that the diameter, morphology, chemical interactions, and incorporation efficiency were similar to the batch obtained by Castro et al. (2020), ensuring that the material's functionality would be preserved and possible to continue the study. This step is of fundamental importance to guarantee the standardization of each batch of nanoformulation obtained.

Because of these characterization results, it can be stated that the process for obtaining nanoparticles used in this study is standardized and reproducible, which is essential to maintain the OPG nanoformulation properties even when obtaining new batches. Thus, it is possible to affirm that the nanoparticles developed have the same physical and chemical characteristics as those obtained by Castro et al. (20) as desired, being possible to proceed with the study for further investigation of physical and chemical characterization, safety, antioxidant potential, and antimicrobial activity.

Zeta potential

Fig 2 shows OPG Zeta Potential values with the pH variation that resulted in a change in charge density around the nanoparticle. Low zeta potential values show a tendency to settle in

an aqueous medium. Still, this low charge indicates steric stability of OPG as they are values with charges close to zero, so they can aggregate more quickly, but they also have eased to disperse(43).

Figure 2. Zeta Potential of buriti oil nanoencapsulated in porcine gelatin under different pH conditions.

As the nanoparticle is composed of porcine gelatin and has an amphoteric character, changes in pH alter the OPG surface charge between positive and negative values(21). It is noteworthy that this change in surface charge influenced by the pH of the medium is an interesting feature when working with the application of nanoparticles in biofilms(44,45).

Thermogravimetry (TG) and differential thermal analysis (DTA)

The TG/DTA curves of crude buriti oil, OPG, porcine gelatin, and Tween 20 are shown in Fig 3.

Figure 3. Thermogravimetry graphs and differential thermal analysis. a) Porcine gelatin b) Tween 20 c) Buriti oil d) OPG.

Buriti oil presents TG mass loss onset at approximately 365 °C associated with three endothermic events in the DTA between 239 and 446ºC, which may be related to the complete breakdown of fatty acids(46). The total degradation of buriti oil ends at 596 ºC and thus presents two stages of mass loss, with a total loss of 98.87%. The thermal stability of buriti oil is high when compared to other vegetable oils such as coconut oil (257 ºC) and sesame oil (282 ºC)(46,47).

The OPG TG curve in Fig 3B shows an initial and progressive mass loss up to approximately 100 °C, corresponding to the endothermic peak in the DTA, which may indicate water evaporation in the sample(48). Then, the decomposition of the nanoparticle starts before the buriti oil at a temperature onset of 248 °C. After this event, mass loss occurs during the endothermic decomposition peak in both free buriti oil and nanoformulation. The mass loss is 95% and 83%, respectively, which may be related to the decomposition of the material wall(49). The OPG DTA curve presents an exothermic peak characteristic of the sample crystallization, as the XRD indicates that the sample is partially amorphous, confirming this event.

The TG curve of porcine gelatin in Fig 3C had an initial thermal degradation between 35 and 160 °C, which may be related to the loss of adsorbed and bound water commonly seen with

air-dried gelatin(45), confirmed by the endothermic peak in the DTA in the same temperature range. It still has the beginning of its degradation between 250 °C and 656 °C, with two stages of very evident mass loss and with a total loss of 98%. In the DTA curve, it is possible to confirm the degradation with two endothermic peaks and one exothermic peak in this same interval.

The Tween 20 TG curve in Fig 3D showed four decomposition stages. The first stage corresponds to water loss (32-196ºC) and the DTA curve presenting an endothermic peak in this temperature range, which corresponds to dehydration or desolvation. The beginning of Tween 20 degradation can be seen in the TG curve at 198 °C with the end of degradation at 654 °C, with total mass loss in the TG curve of 100%.

In evaluating the TG curves, it was possible to note that buriti oil is more stable than the other samples. However, although OPG has a lower initial degradation temperature than pure buriti oil, it is still a very high temperature for a product with a greater possibility of handling and, consequently, application than pure oil. Tween 20 and porcine gelatin may have contributed to maintaining buriti oil's stability in the form of a nanoparticle and the same amount of degradation steps. Escobar-García et al.(50) performed a thermal analysis of oil rich in free eicosapentaenoic acid (EPA) and microencapsulated in concentrated whey protein and observed that the encapsulated sample had a delay in the degradation curve than the unencapsulated sample, with this result being attributed to the protection of the encapsulating agent(51).

Table 1 shows the steps of oil decomposition, encapsulating agent, Tween 20, and nanoencapsulated. Buriti oil has two degradation stages, with the first stage between 239.09 °C - 446.21 °C with a significant mass loss of 87.45%. The initial degradation temperature is similar to the data found for buriti oil in the study of Lima et al. (231.9 °C), but it differs concerning the mass loss, which was 59.6%. The results of the studies are often contradictory, as vegetable oils are very complex because they have a complex chemical composition, including lipid oxidation products, free fatty acids, phenolic compounds, glycerides, and non-glycerides that are present in oils in different proportions(52).

Table 1: The stages (TG) and mass loss of buriti oil, porcine gelatin, Tween 20 and OPG

Sample	Number of decomposition steps	Start decomposition temperature/ °C	End decomposition temperature /°C	Mass loss (%)
Buriti oil	1	239.09	446.21	87.45
	2	446.00	596.00	11.42
Porcine gelatin	1	38.10	166.71	10.07
	2	252.69	380.57	38.66
	3	380.57	648.62	47.20
Tween 20	1	32.84	196.17	3.80
	2	198.34	313.14	20.35
	3	313.14	432.29	71.42
	4	432.29	654.29	4.70
OPG	1	36.62	107.08	4.35
	2	218.00	388.47	44.32
	3	388.47	463.93	32.93
	4	463.93	672.59	15.93

The physicochemical characteristics of buriti oils are different due to their location and other factors(53). There are disagreements among authors regarding these degradation steps. Garcia et al.(51) found only two steps for the degradation of buriti oil since the mass loss was initially high. There are some hypotheses for these differences in vegetable oils, mainly regarding their fatty acid composition. Lima et al.(54) associated the mass losses in two stages: the triglycerides' evaporation and/or pyrolysis. Garcia et al.(51) reported the first and second weight loss as the oxidation of unsaturated and saturated fatty acids, respectively. The third step was reported as the decomposition of the polymer formed during the oxidation process.

OPG showed four degradation stages, and in the first stage, there was a mass loss of 4.35% at an initial temperature of 36.62 °C to 107.08 °C. Porcine gelatin and Tween 20 present an initial degradation temperature between 38.10 °C and 32.84 °C, respectively. This data suggests that the initial degradation observed in OPG may come from the degradation of gelatin and Tween 20. The second stage of nanoformulation degradation occurs at 218 to 388.47°C, with a mass loss of less than 50%, partially corresponding to buriti oil. This data shows the OPG mass loss anticipation compared to free buriti oil, and however, the oil has a loss greater than 87%.

According to the data, it is observed that the stability of the free oil was higher in terms of initial decomposition temperature. Still, its degradation is very drastic, while the nanoformulation loses mass more slowly. It is important to emphasize that OPG is more stable than porcine gelatin and Tween 2 0 and that it has stability approaching that of pure buriti oil. In a way, OPG has significant advantages as it is a product with better handling, is more innovative, and maintains high oil stability. These data are important for the food area since nanoencapsulation exerts the possibility of using the product at different temperatures.

Table 2 presents the data of the DTA curves for buriti oil, encapsulating agent, Tween 20, and nanoencapsulated. It is possible to observe that all samples showed four endothermic or exothermic events characteristic of the analyzed samples. DTA can accurately measure the enthalpic transition temperatures of energy absorption and release. In this case, it is observed that the buriti oil and the nanoencapsulated present four characteristic endothermic events of their decomposition. The first endothermic decomposition event in buriti oil occurs at 259.03 °C with a maximum peak of 282.98 °C (ΔH = 115.86). In comparison, this event seems to happen at a lower temperature of 214.05 °C in OPG, with a maximum peak at 245.47 ºC (ΔH=286.40), corroborating the data obtained in the TG. It is also possible to visualize the endothermic peak in the nanoformulation characteristic of the dehydration of the encapsulating agent and Tween 20 around 31 and 78 ºC.

Table 2: The stages DTA of buriti oil, porcine gelatin, Tween 20, and OPG.

Sample	Stage	T_{onset}/ °C	T_{peak}/ °C	**Δ**H (J/g)
Buriti oil	1	259.03 ↓	282.98	115.86
	2	350.09 ↓	381.57	$2.85x10^{6*}$
	3	400.41 ↓	432.56	964.53
	4	476.98 ↓	480.94	98.64
Porcine Gelatin	1	37.69 ↓	84.81	509.39
	2	215.42 ↓	227.48	12.87
	3	399.46 ↓	409.59	370.38
	4	633.63 ↑	641.97	114.80
Tween 20	1	31.77 ↓	78.14	592.78
	2	281.13 ↓	305.44	$1.90x10^{6*}$
	3	397.64 ↑	402.05	6.03

	4	500.21↓	523.07	354.57
OPG	1	31.78 ↓	58.95	219.36
	2	214.05 ↓	245.47	286.40
	3	387.36 ↓	431.87	$6.18x10^{6}$*
	4	583.34 ↓	601.43	154.45

*J/Kg; ↓ Endothermic ↑ Exothermic

Cytotoxicity - MTT assay

The toxicity investigation of nanoparticles from essential oils, vegetables, and algae is necessary due to the unpredictable behavior that nanoscale materials have demonstrated(55). The International Organization for Standardization (ISO 10993) compiles several tests on biomaterial/biological tissue interaction and determines that biocompatibility starts with evaluating material from cytotoxicity(56). Part 5 of ISO 10993 is responsible for suggesting the tests and conditions necessary for evaluating the cytotoxicity test(56). According to the standard, a reduction greater than 30% in cell viability in the tests is indicative of the material’s cytotoxicity.

Fig 4 shows the MTT assay result. According to the data at the tested concentrations (0.5, 2.5, and 5 µg.mL^{-1}), crude buriti oil and OPG were not cytotoxic to CHO cells. There is no significant difference between buriti oil and OPG. Zanatta et al.(57) evaluated the cytotoxic effect of an emulsion containing buriti oil on 3T3 cells (1000 mg.mL^{-1}) and found no toxic effect, corroborating the results found in this work.

Figure 4. Cell viability (%) through the MTT assay in CHO-KI cells evaluated at different times (A – 24h, B – 48h, and C – 72h) and concentrations of buriti oil and OPG.

When reduced to < 70%, the viability has cytotoxic potential, which was not seen in this study(56). Therefore, the buriti oil and the nanoformulation results are favorable because it does not present toxicity to the cells, assuming that their use is safe.

Total antioxidant capacity (TAC)

TAC is determined using the phosphomolybdenum blue complex to reduce Mo^{+6} to Mo^{+5} by antioxidant compounds and the formation of green Mo^{+5} complexes(58). The results obtained (Table 3) showed that the nanoencapsulation process increased the antioxidant capacity of buriti

oil threefold. This result can be explained by the greater dispersibility in water observed for the OPG due to the obtained particle size, suggesting an increase in the contact surface for chemical interaction with water. Besides, chemical interactions between the materials of the system, as evidenced in the FTIR, consequently increased the antioxidant activity of the encapsulate (20,22).

.

Table 3 – Total antioxidant capacity of buriti oil and OPG

Samples	mg AA. g^{-1}
Buriti oil	14.60 (1.63)[a]
OPG	48.34 (3.71)[b]

OPG: buriti oil nanoencapsulated in a porcine gelatin
AA: ascorbic acid
Mean and standard deviation (SD), n = 3. The different lowercase letters indicate a statistical difference.

TAC analyses for crude and encapsulated buriti oil were not found in the literature. Ribeiro et al.(59) evaluated the total antioxidant capacity of oil from faveleira seeds. The value found was 0.04 (0.00) mg AA. g^{-1}, constituting a lower result than its methanolic fraction [0.08 (0.00) mg AA. g^{-1}] and higher than its non-polar fraction [0.02 (0.00) mg AA. g^{-1}].

Silva et al.(60) extracted astaxanthin from shrimp of the *Litopenaeus vannamei* species using soybean oil and evaluated the antioxidant potential. The TAC results showed that the astaxanthin-pigmented soybean oil had higher antioxidant activity than soybean oil (control).

Zhong and Shahidi(61) compared the antioxidant activities of polar and non-polar compounds. They observed that a higher concentration of non-polar samples is needed to achieve the optimal antioxidant activity. These data corroborate this study since the buriti oil concentration required to present antioxidant activity was higher than that of OPG, which has a solubility in water.

Reducing power

The reducing power assesses the ability of a sample to donate electrons in the presence of ferric chloride under acidic conditions and thus reduce Fe^{+3} to Fe^{+2}(62). The results (Fig 5) showed that a lower OPG concentration than crude buriti oil was necessary to present similar

antioxidant activity. Therefore, it is noteworthy that there is 0.98 mg of buriti oil in 2 mg.mL^{-1} of OPG according to the encapsulation efficiency and considering the amount and proportion of the encapsulating agents. It is possible to note that this concentration was sufficient to promote a reduction from Fe^{+3} to Fe^{+2} in 100%.

Figure 5. Reducing power test of crude buriti oil (A) and OPG nanoformulation. The data obtained presented parametric distribution. Therefore, the ANOVA test with Tukey's post-test was used to determine the significant differences. *Equal letters indicate that the values do not differ statistically ($p > 0.05$).

On the other hand, 1 mg. mL^{-1} of crude buriti oil promoted a reduction from Fe^{+3} to Fe^{+2} by only 0.74%. The encapsulate concentration must take into account that there are also encapsulating agents in addition to the oil (swine gelatin and Tween 20 in a ratio of 1:2.15). In addition, encapsulation efficiency must also be considered, as not all of the oil placed in the system was encapsulated.

Based on this, the result obtained demonstrates that the nanoencapsulation process enhanced the reducing power of buriti oil, with the reduction being greater as the concentration used in the test increases. No reduction power studies were found for crude or encapsulated buriti oil. In analyzing turmeric essential oil, Mau et al.(63) observed that the reducing power increases as the concentration of oil increases, corroborating the data found in this work.

Antioxidant activity by 2,2'-azinobis-3-ethylbenzothiazoline-6-sulfonic acid (ABTS•)

Nanoencapsulation is known to increase the antioxidant potential of natural molecules(64–66). ABTS radical scavenging was evaluated using buriti oil and OPG (Table 4).

Table 4 – Antioxidant activity (IC 50) buriti oil and OPG.

**Samples**	**IC50 (mg. mL^{-1})**
Buriti oil	4.18 (0.05)^{a}
OPG	0.94 (0.02)^{b}

OPG: buriti oil nanoencapsulated in a porcine gelatin
Mean and standard deviation (SD), n = 3. The different lowercase letters indicate a statistical difference.

The results obtained demonstrate that the technological process of nanoencapsulation favored the antioxidant activity since the amount of OPG used was about four times smaller than that of buriti oil to reach the IC50.

Lira et al.(22) performed an analysis for crude quinoa oil and its three nanoencapsulated formulations (encapsulated with porcine gelatin, encapsulated with whey protein, and porcine gelatin in aqueous phase 1, and encapsulated with whey protein in aqueous phase 1 and gelatin in aqueous phase 2). The IC 50 of the formulations was lower than that of the oil, corroborating the data found in this study.

Eugenol essential oil (EO) was nanoencapsulated by the O/W emulsification method with sodium caseinate, maltodextrin and Tween 80(67). The results showed that the nanoencapsulated eugenol EO had a lower IC50 for the ABTS radical assay than crude eugenol(67).

This analysis showed that the OPG nanoparticle has greater antioxidant activity than its unencapsulated form. Thus, nanoscale diameter allows for increased contact surface for chemical interactions, increased water dispersibility, bioaccessibility, and bioavailability compared to micro or macro scale size (12). Therefore, nanoencapsulated oils are protected against oxidation of their bioactive constituents and have a higher solubility in water (20,22,68). This difference is relevant for its applications in food matrices to prolong the biological action and provide health benefits (65,69).

Determination of the modulating activity of antibiotics

Due to the emergence of multidrug-resistant strains to antibiotics, combined drugs are used against these resistance mechanisms(70). Antimicrobial resistance, in general, alters antibiotic action through the following mechanisms: modification of the antimicrobial target (decreased drug affinity), decreased drug absorption, activation of efflux mechanisms to expel the harmful compound (overexpression efflux pumps), or global changes in critical metabolic pathways through the modulation of regulatory networks (71). One example is plant extracts that exhibit synergistic activity against microorganisms, as they present a complex mixture of natural

products and present a low risk in the development of bacterial resistance(72). Some natural products such as plant extracts, essential oils(73), and fixed oils(74) have modified the activity of antibiotics. Fig 6 shows the result of the MIC of buriti oil and OPG combined with norfloxacillin and gentamycin antibiotics.

Figure 6. MIC values (µg.mL^{-1}) of antibiotics in a bacterial growth assay in the presence of buriti oil (A) and buriti oil nanoencapsulated in porcine gelatin (OPG) (B). The tests were performed under the following conditions: norfloxacillin (white bar), norfloxacillin + crude buriti oil/OPG (light gray bar), gentamicin (dark gray bar), and gentamicin + crude buriti oil/OPG (black bar). ***p<0.0001.

Gentamycin is a drug used to treat infections, especially by Gram-negative bacteria, such as *E. coli* and *P. aeruginosa*, and it can also be used in combination(75). Its MIC for *E. coli* and *P. aeruginosa* is 1 µg.mL^{-1} and 2 µg.mL^{-1}, respectively(76,77). Norfloxacin is a broad-spectrum antibiotic used for Gram-positive and Gram-negative bacteria(78); it has a MIC of 25 µg.mL^{-1} for *E. coli*, 2 µg.mL^{-1} for *P. aeruginosa,* and 128 µg.mL^{-1} for *S. aureus*(79–81). Fixed buriti oil has a MIC >1024   for several strains, including *E. coli*, *P. aeruginosa,* and *S. aureus*(82).

Modulation with vegetable oils promotes better action on bacteria when there is a synergism of this combination, reducing the amount or increasing the medication used(82). The mechanisms of action between antibiotic and oil(83,84) may be related to the interaction with the lipid bilayer and cell membrane, affecting the respiratory chain and energy production by the bacteria(85), or even interference with bacterial enzymatic systems that may enhance the mechanism of action of antibiotics in combination with oils(86).

The combination of antibiotics with crude buriti oil (Fig 5A) and OPG (Fig 6B) showed greater efficiency in inhibiting the action of the *E. coli* bacteria when compared to isolated antibiotics. The combination of norfloxacillin and gentamycin antibiotics with the crude buriti oil reduced the MIC by 93.75% and 75%, respectively. Combining the same antibiotics with OPG reduced the MIC of norfloxacillin by 98% and gentamycin by 75% in *E. coli*. For gentamycin, the nanoencapsulated oil maintained the modulating activity of the crude oil in 75%.

Leão et al.(87) observed that a nanoemulsion containing buriti oil inhibited the microbial growth of *E. coli* by 61% at a concentration of 3.14 µg.mL^{-1}, demonstrating a bacteriostatic effect. With this, the researchers observed that the physicochemical characteristics of the nanoemulsion

contribute to its biological activity. The particle size can directly affect the antimicrobial activity of the emulsion based on essential oils, meaning a smaller size (nanometric scale) induces a greater inactivation of *E. coli* when compared to conventional emulsions(87).

Norfloxacillin alone, when compared with norfloxacillin combined with crude buriti oil (Fig 6A), did not present modulating activity for *S. aureus* with a statistically significant difference ($p>0.05$). However, the antibiotic activity combined with OPG decreased the MIC by 50% (Fig 6B). For gentamycin combined with crude oil such as OPG, there was an antagonism characterized by an increase in MIC by 100% in both demonstrated cases. Gentamycin does not act on Gram-positive bacteria, as *S. aureus*(75), showing that the nanoformulation maintained the crude buriti oil activity. Two main mechanisms are involved in antagonistic effects associated with natural products and antibiotics: chelation of antibiotic constituents by the natural product and competition of substances for the same binding site(88).

Norfloxacillin combined with buriti oil and OPG had greater inhibition for *P. aeruginosa* bacteria than norfloxacillin alone, with a statistical difference between them ($p<0.05$) and a 99% reduction in MIC with OPG and 97% with crude buriti oil. Gentamycin showed a significant difference in the two combinations (with free oil and OPG), in which there was a 50% reduction in MIC. In this case, both the free oil and OPG had a synergistic effect with the antibiotics.

Oils modify the antimicrobial effect depending on the antibiotic, oil tested and the bacterial species analyzed(14), which can be attributed to an interaction of the oil with the plasma membrane of the bacteria, increasing the permeability of the plasma membrane to the antibiotic(14,89,90).

Pereira et al.(82) used two antibiotics (gentamycin and amikacin) and two bacterial strains (*S. aureus* and *E. coli*) to verify the modulating activity of fixed buriti oil. The synergistic effect was observed in both antibiotics for *S. aureus*, with no MIC changes in *E. coli*.

The combination of buriti oil and OPG associated with norfloxacillin presenting FICi values ≤ 0.5 shows the synergistic effect for *E. coli* e *P. aeruginosa (Table 5).*

Table 5: Fractional inhibitory concentration (FIC) and fractional inhibitory concentration indices (FICi) of Buriti oil, OPG, norfloxacillin, and gentamycin.

**Microorganism**	**MIC** $_{buriti\ oil}$	**MIC** $_{Norfloxacillin}$	**MIC** $_{Norfloxacillin+Buriti\ oil}$	**FICi**

	**(μg.mL^{-1})**	**(μg.mL^{-1})**	**(μg.mL^{-1})**	
E. coli	64	128	8	0.2
P. aeruginosa	128	512	16	0.1
	**MIC $_{OPG}$ (μg.mL^{-1})**	**MIC $_{Norfloxacillin}$ (μg.mL^{-1})**	**MIC $_{Norfloxacillin+OPG}$ (μg.mL^{-1})**	**FICi**
E. coli	16	128	2	0.1
P. aeruginosa	32	512	4	0.1

MIC: minimum inhibitory concentration, FIC: Fractional inhibitory concentration, FICi: fractional inhibitory concentration index, OPG: buriti oil nanoencapsulated in porcine gelatin.

Combinations of natural products and antibiotics(91) can affect more than one target for bacterial growth inhibition. This strategy is known as “herbal shotgun” or “synergistic multi-effect targeting,” in which different therapeutic components collaborate in a synergistic-agonistic way(88).

It is believed that both the antibacterial potential and the modulating activity of antibiotics attributed to vegetable oils are, at least in part, associated with the fatty acids present in the composition of the product since some fatty acids have already been shown to be able to improve the antimicrobial activity. And inhibit bacterial growth(15,92). It is reported that the potential of vegetable oils to act as antibacterial modulators is in part associated with the detergent property of fatty acids against the amphipathic structure of bacterial cell membranes (15). In this context, the synergistic effect observed against *E.coli* and *P. aeroginosa* may be associated with the detergent properties of fatty acids.

The literature points out that long-chain unsaturated fatty acids, such as oleic and palmitic, demonstrate antibacterial activity. In addition, the conjugated use of fatty acids and peptides or antibiotics can potentiate antimicrobial activity due to increased membrane permeability (93,94). This ability to solubilize membrane components (lipids and proteins) creates gaps in this structure that will affect metabolic processes essential for acquiring energy for the bacterial cell, such as the electron transport chain and oxidative phosphorylation. Membrane damage can also inhibit enzyme activity and toxic peroxidation (95). In addition, the presence of hydrophobic compounds in vegetable oils can increase the cell's permeability to antibiotics,

resulting in greater efficiency and reducing the minimum concentration necessary for the antibiotic to act against the bacteria (15,96).

The present study is innovative and unprecedented in the literature because it manages to evaluate the effect of the nanoformulation in the modulation of the antibiotic activity of known drugs, and not only of the non-encapsulated vegetable oil as presented in other studies. Thus, this study showed promising results for using free buriti oil and OPG in association with modifying antibiotic resistance against multiresistant strains such as *E. coli* and *P. aeroginosa*.

OPG is synthesized using low-cost materials. In addition, obtaining it is scalable, and drying by lyophilization is already used in the food industry. Thus, the nanoformulation has the potential to be incorporated into foods to add nutritional value due to the presence of buriti oil and its bioactive compounds, and also potential use in food packaging to act in the inhibition of pathogenic bacteria. Thus, OPG presents itself as a viable alternative to promote the preservation and/or enhancement of the oil's bioactive properties, which favors its use by the food and pharmaceutical industry, in addition to generating benefits to consumers' health. The application of nanotechnology in materials of plant origin promotes the valorization of this resource. It can contribute to the local development of the buriti-producing region within the perspective of circular economy and sustainable production.

References

1. Cattani IM, Baruque-Ramos J. Brazilian buriti palm fiber (Mauritia flexuosa Mart.). In Natural fibres: Advances in science and technology towards industrial applications. Springer, Dordrecht. 2016;89–98. Available from: https://doi.org/10.1007/978-94-017-7515-1

2. Cândido TLN, Silva MR, Agostini-Costa TS. Bioactive compounds and antioxidant capacity of buriti (Mauritia flexuosa L.f.) from the Cerrado and Amazon biomes. Food Chem [Internet]. 2015;177:313–9. Available from: http://dx.doi.org/10.1016/j.foodchem.2015.01.041

3. Cândido TLN, Silva MR. Comparison of the physicochemical profiles of buriti from the Brazilian Cerrado and the Amazon region. Food Sci Technol. 2017;37:78–82. Available from: https://doi.org/10.1590/1678-457X.32516

4. Silva SM, Sampaio KA, Taham T, Rocco SA, Ceriani R, Meirelles AJA. Characterization of oil extracted from buriti fruit (Mauritia flexuosa) grown in the Brazilian Amazon Region. JAOCS, J Am Oil Chem Soc. 2009;86(7):611–6. Available from:

https://doi.org/10.1007/s11746-009-1400-9

5. Ferreira BS, De Almeida CG, Faza LP, De Almeida A, Diniz CG, Da Silva VL, et al. Comparative properties of amazonian oils obtained by different extraction methods. Molecules. 2011;16(7):5874–85. Available from: https://doi.org/10.3390/molecules16075875

6. de Souza FG, de Araújo FF, de Paulo Farias D, Zanotto AW, Neri-Numa IA, Pastore GM. Brazilian fruits of Arecaceae family: An overview of some representatives with promising food, therapeutic and industrial applications. Food Res Int. 2020;138(September). Available from: https://doi.org/10.1016/j.foodres.2020.109690

7. Zanatta CF, Mitjans M, Urgatondo V, Rocha-Filho PA, Vinardell MP. Photoprotective potential of emulsions formulated with Buriti oil (Mauritia flexuosa) against UV irradiation on keratinocytes and fibroblasts cell lines. Food Chem Toxicol. 2010;48(1):70–5. Available from: https://doi.org/10.1016/j.fct.2009.09.017

8. Aquino JDS, Vasconcelos MHDA, Pessoa DCNDP, Soares JKB, Prado JPDS, Mascarenhas RDJ, et al. Intake of cookies made with buriti oil (: Mauritia flexuosa) improves vitamin A status and lipid profiles in young rats. Food Funct. 2016;7(10):4442–50. Available from: https://doi.org/10.1039/c6fo00770h

9. Hamacek FR, Della Lucia CM, Silva BP da, Moreira AVB, Pinheiro-Sant'ana HM. Buriti of the cerrado of Minas Gerais, Brazil: physical and chemical characterization and content of carotenoids and vitamins. Food Sci Technol. 2018;38(suppl 1):263–9. Available from: https://doi.org/10.1590/fst.15417

10. Galli F, Azzi A, Birringer M, Cook-Mills JM, Eggersdorfer M, Frank J, et al. Vitamin E: Emerging aspects and new directions. Free Radic Biol Med. 2017;102(June 2016):16–36. Available from: https://doi.org/10.1016/j.freeradbiomed.2016.09.017

11. Leslie MA, Cohen DJA, Liddle DM, Robinson LE, Ma DWL. A review of the effect of omega-3 polyunsaturated fatty acids on blood triacylglycerol levels in normolipidemic and borderline hyperlipidemic individuals. Lipids Health Dis. 2015;14(1). Available from: https://doi.org/10.1186/s12944-015-0049-7

12. Fuentes E, Rodríguez-Pérez W, Guzmán L, Alarcón M, Navarrete S, Forero-Doria O, et al. Mauritia flexuosa presents in vitro and in vivo antiplatelet and antithrombotic activities. Evidence-based Complement Altern Med. 2013;2013. Available from: https://doi.org/10.1155/2013/653257

13. Almeida MMB, de Sousa PHM, Arriaga ÂMC, do Prado GM, Magalhães CE de C, Maia GA, et al. Bioactive compounds and antioxidant activity of fresh exotic fruits from northeastern Brazil. Food Res Int. 2011 Aug 1;44(7):2155–9. Available from: https://doi.org/10.1016/J.FOODRES.2011.03.051

14. Oliveira RAG de, Lima EO, Vieira WL, Freire KRL, Trajano VN, Lima IO, et al. Estudo da interferência de óleos essenciais sobre a atividade de alguns antibióticos usados na clínica. Rev Bras Farmacogn. 2006;16(1):77–82. Available from: https://doi.org/10.1590/s0102-695x2006000100014

15. Saraiva RA, Matias EFF, Coutinho HDM, Costa JGM, Souza HHF, Fernandes CN, et al. Synergistic action between Caryocar coriaceum Wittm. fixed oil with aminoglycosides in vitro. Eur J Lipid Sci Technol. 2011;113(8):967–72. Available from: https://doi.org/10.1002/ejlt.201000555

16. Moser P, Ferreira S, Nicoletti VR. Buriti oil microencapsulation in chickpea protein-pectin matrix as affected by spray drying parameters. Food Bioprod Process [Internet]. 2019;117:183–93. Available from: https://doi.org/10.1016/j.fbp.2019.07.009

17. Moser P, Ferreira S, Nicoletti VR. Buriti oil microencapsulation in chickpea protein/pectin matrix as affected by spray drying parameters. Food Bioprod Process. 2019;34. Available from: https://doi.org/10.1016/j.fbp.2019.07.009

18. Santana AA, Kurozawa LE, de Oliveira RA, Park KJ. Influence of Process Conditions on the Physicochemical Properties of Pequi Powder Produced by Spray Drying. Dry Technol. 2013;31(7):825–36. Available from: https://doi.org/ 10.1080/07373937.2013.766619

19. Sharma S, Cheng S-F, Bhattacharya B, Chakkaravarthi S. Efficacy of free and encapsulated natural antioxidants in oxidative stability of edible oil: Special emphasis on nanoemulsion-based encapsulation. Trends Food Sci Technol [Internet]. 2019 Sep;91(July):305–18. Available from: https://doi.org/10.1016/j.tifs.2019.07.030

20. Castro GMMA, Passos TS, Nascimento SS da C, Medeiros I, Araújo NK, Maciel BLL, et al. Gelatin nanoparticles enable water dispersibility and potentialize the antimicrobial activity of Buriti (Mauritia flexuosa) oil. BMC Biotechnol. 2020;20(1):1–13. Available from: https://doi.org/10.1186/s12896-020-00649-4

21. Kaushik P, Dowling K, Barrow CJ, Adhikari B. Microencapsulation of omega-3 fatty acids: A review of microencapsulation and characterization methods. J Funct Foods. 2015;19:868–81. Available from: https://doi.org/10.1016/j.jff.2014.06.029

22. Lira KHD da S, Passos TS, Ramalho HMM, Rodrigues D da SR, Vieira É de A, Cordeiro AMT de, et al. Whey protein isolate-gelatin nanoparticles enable the water-dispersibility and potentialize the antioxidant activity of quinoa oil (Chenopodium quinoa). PLoS One. 2020;30:1–17. Available from: https://doi.org/10.1371/journal.pone.0240889

23. Faridi Esfanjani A, Jafari SM. Biopolymer nano-particles and natural nano-carriers for nano-encapsulation of phenolic compounds. Colloids Surfaces B Biointerfaces. 2016 Oct 1;146:532–43. Available from: https://doi.org/10.1016/J.COLSURFB.2016.06.053

24. Medeiros AK de OC, Gomes C de C, Amaral MLQ de A, Medeiros LDG de, Medeiros I, Porto DL, et al. Nanoencapsulation improved water solubility and color stability of carotenoids extracted from Cantaloupe melon (Cucumis melo L.) [Internet]. Vol. 270, Food Chemistry. Elsevier; 2019 [cited 2019 Oct 1]. 562–572 p. Available from: https://doi.org/10.1016/J.FOODCHEM.2018.07.099

25. El-Messery TM, Altuntas U, Altin G, Özçelik B. The effect of spray-drying and freeze-drying on encapsulation efficiency, in vitro bioaccessibility and oxidative stability of krill oil nanoemulsion system. Food Hydrocoll. 2020;106(March). Available from: https://doi.org/10.1016/j.foodhyd.2020.105890

26. Prieto P, Pineda M, Aguilar M. Spectrophotometric quantitation of antioxidant capacity through the formation of a …. Anal Biochem [Internet]. 1999;269:337–41. Available from: Available from: https://doi.org/ 10.1037/a0037168

27. Wang J, Zhang Q, Zhang Z, Li Z. Antioxidant activity of sulfated polysaccharide fractions extracted from Laminaria japonica. Int J Biol Macromol [Internet]. 2008 Mar 1 [cited 2019 Feb 27];42(2):127–32. Available from: https://doi.org/10.1016/J.IJBIOMAC.2007.10.003

28. Rufino M do S, Alves RE, Brito ES De, Morais SM De, Sampaio CDG, Saura-calixto FD. Metodologia Científica: Determinação da atividade antioxidante total em frutas pela captura do radical livre ABTS. Comunicado Técnico 128. 2007. p. 0–3. Available from: ISSN 1679-6535

29. Harb TB, Torres PB, Pires JS, Santos DYAC, Chow F. Ensaio em microplaca do potencial antioxidante através do sistema quelante de metais para extratos de algas. Inst Biociências da Univ São Paulo. 2016;(April 2018):2–6. Available from: https://doi.org/10.13140/RG.2.2.35838.69446

30. de Oliveira GLR, Medeiros I, Nascimento SS da C, Viana RLS, Porto DL, Rocha HAO, et al. Antioxidant stability enhancement of carotenoid rich-extract from Cantaloupe melon (Cucumis melo L.) nanoencapsulated in gelatin under different storage conditions. Food Chem. 2021;348(September 2020). Available from: https://doi.org/10.1016/j.foodchem.2021.129055

31. Bezerra CF, Camilo CJ, do Nascimento Silva MK, de Freitas TS, Ribeiro-Filho J, Coutinho HDM. Vanillin selectively modulates the action of antibiotics against resistant bacteria. Microb Pathog [Internet]. 2017;113(September):265–8. Available from: https://doi.org/10.1016/j.micpath.2017.10.052

32. Patel J.B., Cockerill R.F., Bradford A.P., Eliopoulos M.G., Hindler A.J., Jenkins G.S., Lewis S.J., Limbago B., Miller A.L., Nicolau P.D., Pwell M., Swenson M.J., Traczewski M.M., Turnidge J.D. WPMZLB. M07-A10: Methods for Dilution Antimicrobial Susceptibility Tests for Bacteria That Grow Aerobically; Approved Standard—Tenth Edition. CLSI (Clinical Lab Stand Institute). 2015;35(2). Available from:

https://doi.org/10.1007/s00259-009-1334-3

33. Rocha RR, Matos MNC, Guerrero JAP, Cavalcante RMB, Melo RS, Azevedo ÁMA, et al. Comparative study of the chemical composition, antibacterial activity and synergic effects of the essential oils of Croton tetradenius baill. And C. pulegiodorus baill. Against Staphylococcus aureus isolates. Microb Pathog. 2021;156(January). Available from: https://doi.org/10.1016/j.micpath.2021.104934

34. Chen L, Remondetto GE, Subirade M. Food protein-based materials as nutraceutical delivery systems. Trends Food Sci Technol. 2006;17(5):272–83.

35. Kumar A, Dixit CK. Methods for characterization of nanoparticles. In: Advances in Nanomedicine for the Delivery of Therapeutic Nucleic Acids. 2017. p. 44–58. Available from: https://doi.org/10.1016/B978-0-08-100557-6.00003-1

36. Danaei M, Dehghankhold M, Ataei S, Hasanzadeh Davarani F, Javanmard R, Dokhani A, et al. Impact of particle size and polydispersity index on the clinical applications of lipidic nanocarrier systems. Pharmaceutics. 2018;10(2):1–17. Available from: https://doi.org/10.3390/pharmaceutics10020057

37. Silverstein RM, Webster FX, Kiemle DJ. Silverstein-Spectrometric Identification of Organic Compounds 7th ed. 7th ed. New York: John Wiley & Sons; 2005. 550 p. Available from: ISBN 1118311655, 9781118311653

38. Guerrero P, Stefani PMM, Ruseckaite RAA, de la Caba K. Functional properties of films based on soy protein isolate and gelatin processed by compression molding. J Food Eng [Internet]. 2011 Jul 1 [cited 2019 Oct 14];105(1):65–72. Available from: https://doi.org/10.1016/j.jfoodeng.2011.02.003

39. Esfahani R, Jafari SM, Jafarpour A, Dehnad D. Loading of fish oil into nanocarriers prepared through gelatin-gum Arabic complexation. Food Hydrocoll [Internet]. 2019;90(December 2018):291–8. Available from: https://doi.org/10.1016/j.foodhyd.2018.12.044

40. Batista JS, Olinda RG, Medeiros VB, Rodrigues CMF, Oliveira AF, Paiva ES, et al. Atividade antibacteriana e cicatrizante do óleo de buriti Mauritia flexuosa L. Ciência Rural. 2012;42(1):136–41. Available from: https://doi.org/10.1590/S0103-84782012000100022

41. Sagiri SS, Anis A, Pal K. Review on Encapsulation of Vegetable Oils: Strategies, Preparation Methods, and Applications. Polym - Plast Technol Eng. 2016;55(3):291–311. Available from: https://doi.org/10.1080/03602559.2015.1050521

42. Can Karaca A, Low NH, Nickerson MT. Potential use of plant proteins in the microencapsulation of lipophilic materials in foods. Trends Food Sci Technol. 2015;42(1):5–12. Available from: https://doi.org/10.1016/j.tifs.2014.11.002

43. da Silva BC, de Oliveira M, Ferreira JGL, Sierakowski MR, Simas-Tosin FF, Orth ES, et al. Polyelectrolyte complexes from gum arabic and gelatin: Optimal complexation pH as a key parameter to obtain reproducible microcapsules. Food Hydrocoll. 2015;46:201–7. Available from: https://doi.org/10.1016/j.foodhyd.2014.12.022

44. Wu Y, Guo J, Yang W, Wang C, Fu S. Preparation and characterization of chitosan-poly(acrylic acid) polymer magnetic microspheres. Polymer (Guildf). 2006;47(15):5287–94. Available from: https://doi.org/10.1016/j.polymer.2006.05.017

45. Zhao Y, Sun Z. Effects of gelatin-polyphenol and gelatin–genipin cross-linking on the structure of gelatin hydrogels. Int J Food Prop. 2018;20(3):S2822–32. Available from: https://doi.org/10.1080/10942912.2017.1381111

46. Durães JA, Drummond AL, Pimentel TAPF, Murta MM, Moreira SGC, Sales MJA. Thermal and structural behavior of Buriti oil/poly(methyl methacrylate) and Buriti oil/polystyrene materials. J Therm Anal Calorim. 2008;92(2):529–34. Available from: https://doi.org/10.1007/s10973-007-8564-z

47. Jayadas NH, Nair KP. Coconut oil as base oil for industrial lubricants-evaluation and modification of thermal, oxidative and low temperature properties. Tribol Int. 2006;39(9):873–8. Available from: https://doi.org/10.1016/j.triboint.2005.06.006

48. Klein T, Longhini R, Bruschi ML, De Mello JCP. Microparticles containing Guaraná extract obtained by spray-drying technique: Development and characterization. Rev Bras Farmacogn. 2015;25(3):292–300. Available from: https://doi.org/10.1016/j.bjp.2015.03.010

49. Liz GR de, Verruck S, Canella MHM, Dantas A, Garcia SG, Maran BM, et al. Stability of bifidobacteria entrapped in goat's whey freeze concentrate and inulin as wall materials and powder properties. Food Res Int. 2020;127(February 2019):10. Available from: https://doi.org/10.1016/j.foodres.2019.108752

50. Escobar-García JD, Prieto C, Pardo-Figuerez M, Lagaron JM. Room temperature nanoencapsulation of bioactive eicosapentaenoic acid rich oil within whey protein microparticles. Nanomaterials. 2021;11(3):1–16. Available from: https://doi.org/10.3390/nano11030575

51. Garcia CC, Franco PIBM, Zuppa TO, Filho NRA, Leles MIG. Thermal stability studies of some cerrado plant oils. J Therm Anal Calorim. 2007;87(3):645–8. Available from: https://doi.org/10.1007/s10973-006-7769-x

52. Vecchio S, Cerretani L, Bendini A, Chiavaro E. Thermal decomposition study of monovarietal extra virgin olive oil by simultaneous thermogravimetry/differential scanning calorimetry: Relation with chemical composition. J Agric Food Chem. 2009;57(11):4793–800. Available from: https://doi.org/10.1021/jf900120v

53. Nde DB, Anuanwen CF. Optimization methods for the extraction of vegetable oils: A review. Processes. 2020;8(2):21. Available from: https://doi.org/10.3390/pr8020209

54. Pereira Lima R, Souza da Luz PT, Braga M, dos Santos Batista PR, Ferreira da Costa CE, Zamian JR, et al. Murumuru (Astrocaryum murumuru Mart.) butter and oils of buriti (Mauritia flexuosa Mart.) and pracaxi (Pentaclethra macroloba (Willd.) Kuntze) can be used for biodiesel production: Physico-chemical properties and thermal and kinetic studies. Ind Crops Prod [Internet]. 2017;97:536–44. Available from: http://doi.org/10.1016/j.indcrop.2016.12.052

55. Schappo FB. Nanopartículas de óleo de palma bruto: estabilidade e citotoxicidade. Programa de Pós-Gaduação em Ciência dos Alimentos. Universidade Federala de Santa Catarina; 2021.

56. International Organization for Standardization. ISO 10993-5: Tests for in vitro cytotoxicity. In: Biological evaluation of medical devices. 2009. p. 42.

57. Zanatta CF, Ugartondo V, Mitjans M, Rocha-Filho PA, Vinardell MP. Low cytotoxicity of creams and lotions formulated with Buriti oil (Mauritia flexuosa) assessed by the neutral red release test. Food Chem Toxicol. 2008;46(8):2776–81. Available from: http://doi.org/10.1016/j.fct.2008.05.001

58. Melo-Silveira RF, Fidelis GP, Pereira Costa MSS, Telles CBS, Dantas-Santos N, de Oliveira Elias S, et al. In vitro antioxidant, anticoagulant and antimicrobial activity and in inhibition of cancer cell proliferation by xylan extracted from corn cobs. Int J Mol Sci. 2012;13(1):409–26. Available from: http://doi.org/10.3390/ijms13010409

59. Ribeiro PPCPPC, E Silva DMDLDMDL, De Assis CFCF, Correia RTPRTP, Damasceno KSFDSCKSFDSC. Bioactive properties of faveleira (Cnidoscolus quercifolius) seeds, oil and press cake obtained during oilseed processing. PLoS One. 2017;12(8):1–12. Available from: http://doi.org/10.1371/journal.pone.0183935

60. Silva DMLE, Damasceno KSFSC, Ribeiro PPC, Alcântra MA, Cordeiro AMTM, Passos TS, et al. Chemical characteristics and antioxidant activity of astaxanthin extracted from shrimp residues using soybean oil. J Braz Chem Soc. 2021;32(6):1277–85. Available from: http://doi.org/10.1371/journal.pone.0183935

61. Zhong Y, Shahidi F. Antioxidant behavior in bulk oil: Limitations of polar paradox theory. J Agric Food Chem. 2012;60(1):4–6. Available from: http://doi.org/10.1021/jf204165g

62. Melo-Silveira RF, Fidelis GP, Viana RLS, Soeiro VC, Da Silva RA, Machado D, et al. Antioxidant and antiproliferative activities of methanolic extract from a neglected agricultural product: Corn cobs. Molecules. 2014;19(4):5360–78. Available from: http://doi.org/10.3390/molecules19045360

63. Mau JL, Lai EYC, Wang NP, Chen CC, Chang CH, Chyau CC. Composition and

antioxidant activity of the essential oil from Curcuma zedoaria. Food Chem. 2003;82(4):583–91. Available from: http://doi.org/10.1016/S0308-8146(03)00014-1

64. Dos Santos PDF, Francisco CRL, Coqueiro A, Leimann FV, Pinela J, Calhelha RC, et al. The nanoencapsulation of curcuminoids extracted from: Curcuma longa L. and an evaluation of their cytotoxic, enzymatic, antioxidant and anti-inflammatory activities. Food Funct. 2019;10(2):573–82. Available from: http://doi.org/10.1039/c8fo02431f

65. Huang X, Liu Y, Zou Y, Liang X, Peng Y, McClements DJ, et al. Encapsulation of resveratrol in zein/pectin core-shell nanoparticles: Stability, bioaccessibility, and antioxidant capacity after simulated gastrointestinal digestion. Food Hydrocoll. 2019;93(December 2018):261–9. Available from: http://doi.org/10.1016/j.foodhyd.2019.02.039

66. Luo Y. Food colloids binary and ternary nanocomplexes: Innovations and discoveries. Colloids Surfaces B Biointerfaces. 2020;196(August):111309. Available from: http://doi.org/10.1016/j.colsurfb.2020.111309

67. Nagaraju PG, Sindhu P, Dubey T, Chinnathambi S, Poornima Priyadarshini CG, Rao PJ. Influence of sodium caseinate, maltodextrin, pectin and their Maillard conjugate on the stability, in vitro release, anti-oxidant property and cell viability of eugenol-olive oil nanoemulsions. Int J Biol Macromol. 2021;183:158–70. Available from: http://doi.org/10.1016/j.ijbiomac.2021.04.122

68. de Campo C, dos Santos PP, Costa TMH, Paese K, Guterres SS, Rios A de O, et al. Nanoencapsulation of chia seed oil with chia mucilage (Salvia hispanica L.) as wall material: Characterization and stability evaluation. Food Chem [Internet]. 2017;234:1–9. Available from: http://dx.doi.org/10.1016/j.foodchem.2017.04.153

69. Bilenler T, Gokbulut I, Sislioglu K, Karabulut I. Antioxidant and antimicrobial properties of thyme essential oil encapsulated in zein particles. Flavour Fragr J. 2015;30(5):392–8. Available from: http://doi.org/10.1002/ffj.3254

70. Coutinho HDM, Costa JGM, Falcão-Silva VS, Siqueira-Júnior JP, Lima EO. Effect of Momordica charantia L. in the resistance to aminoglycosides in methicilin-resistant Staphylococcus aureus. Comp Immunol Microbiol Infect Dis. 2010;33(6):467–71. Available from: http://doi.org/10.1016/j.cimid.2009.08.001

71. de Medeiros VM, do Nascimento YM, Souto AL, Madeiro SAL, Costa VC de O, Silva SMPM, et al. Chemical composition and modulation of bacterial drug resistance of the essential oil from leaves of Croton grewioides. Microb Pathog. 2017 Oct 1;111:468–71. Available from: http://doi.org/10.1016/J.MICPATH.2017.09.034

72. Daferera DJ, Ziogas BN, Polissiou MG. The effectiveness of plant essential oils on the growth of Botrytis cinerea, Fusarium sp. and Clavibacter michiganensis subsp.

michiganensis. Crop Prot. 2003;22(1):39–44. http://doi.org/10.1016/S0261-2194(02)00095-9

73. Coutinho HDM, Costa JGM, Lima EO, Falcão-Silva VS, Siqueira JP. Herbal therapy associated with antibiotic therapy: Potentiation of the antibiotic activity against methicillin - Resistant Staphylococcus aureus by Turnera ulmifolia L. BMC Complement Altern Med. 2009;9:1–4. Available from: http://doi.org/10.1186/1472-6882-9-13

74. Al Ashaal HA, Farghaly AA, Abd El Aziz MM, Ali MA. Phytochemical investigation and medicinal evaluation of fixed oil of Balanites aegyptiaca fruits (Balantiaceae). J Ethnopharmacol. 2010;127(2):495–501. Available from: http://doi.org/10.1016/j.jep.2009.10.007

75. Chen C, Chen Y, Wu P, Chen B. Update on new medicinal applications of gentamicin: Evidence-based review. J Formos Med Assoc. 2014;113(2):72–82. Available from: http://doi.org/10.1016/j.jfma.2013.10.002

76. Garrod LP, Waterworth PM. Effect of medium composition on the apparent sensitivity of Pseudomonas aeruginosa to gentamicin. J Clin Pathol. 1969;22(5):534–8. Available from: http://doi.org/10.1136/jcp.22.5.534

77. Medeiros AA, O'Brien TF, Wacker WEC, Yulug NF. Effect of salt concentration on the apparent in-vitro susceptibility of pseudomonas and other gram-negative bacilli to gentamicin. J Infect Dis. 1971;124(December):S59–64. Available from: http://doi.org/10.1080/10408347.2014.941456

78. Chierentin L, Salgado HRN. Review of Properties and Analytical Methods for the Determination of Norfloxacin. Crit Rev Anal Chem. 2016;46(1):22–39. Available from: http://doi.org/10.1080/10408347.2014.941456

79. Aoyama H, Sato K, Kato T, Hirai K, Mitsuhashi S. Norfloxacin resistance in a clinical isolate of Escherichia coli. Antimicrob Agents Chemother. 1987;31(10):1640–1. Available from: http://doi.org/10.1128/AAC.31.10.1640

80. King A, Warren C, Shannon K, Phillips I. In vitro antibacterial activity of norfloxacin (MK-0366). Antimicrob Agents Chemother. 1982;21(4):604–7. Available from: http://doi.org/10.1128/AAC.21.4.604

81. Diniz-Silva HT, Magnani M, de Siqueira S, de Souza EL, de Siqueira-Júnior JP. Fruit flavonoids as modulators of norfloxacin resistance in Staphylococcus aureus that overexpresses norA. LWT - Food Sci Technol. 2017;85:324–6. Available from: http://doi.org/10.1016/j.lwt.2016.04.003

82. Pereira YF, Costa MDS, Tintino SR, Rocha JE, Rodrigues FFG, Feitosa MK de SB, et al. Modulation of the antibiotic activity by the Mauritia flexuosa (Buriti) fixed oil against methicillin-resistant staphylococcus aureus (MRSA) and other multidrug-resistant (MDR)

bacterial strains. Pathogens. 2018;7(4):1–8. Available from: http://doi.org/10.3390/pathogens7040098

83. Coutinho HDM, Costa JGM, Siqueira-Júnior JP, Lima EO. In vitro anti-staphylococcal activity of Hyptis martiusii Benth against methicillin-resistant Staphylococcus aureus-MRSA strains. Rev Bras Farmacogn. 2008;18(SUPPL.):670–5. Available from: http://doi.org/10.1590/S0102-695X2008000500005

84. Coutinho HDM, Costa JGM, Lima EO, Falcão-Silva VS, Siqueira JP. Enhancement of the antibiotic activity against a multiresistant Escherichia coli by Mentha arvensis L. and chlorpromazine. Chemotherapy. 2008;54(4):328–30. Available from: http://doi.org/10.1159/000151267

85. Nicolson K, Evans G, O'Toole PW. Potentiation of methicillin activity against methicillin-resistant Staphylococcus aureus by diterpenes. FEMS Microbiol Lett. 1999;179(2):233–9. Available from: http://doi.org/10.1016/S0378-1097(99)00417-6

86. Wendakoon CN, Morihiko S. Inhibition of amino acid decarboxylase activity of enterobacter aerogenes by active components in spices. Vol. 58, Journal of Food Protection. 1995. p. 280–3. Available from: http://doi.org/10.4315/0362-028X-58.3.280

87. Leão KMM, Reis LVC, Speranza P, Rodrigues AP, Ribeiro APB, Macedo JA, et al. Physicochemical characterization and antimicrobial activity in novel systems containing buriti oil and structured lipids nanoemulsions. Biotechnol Reports. 2019;24. Available from: http://doi.org/10.1016/j.btre.2019.e00365

88. Wagner H, Ulrich-Merzenich G. Synergy research: Approaching a new generation of phytopharmaceuticals. Phytomedicine. 2009;16(2–3):97–110. Available from: http://doi.org/ 10.1016/j.phymed.2008.12.018

89. Medeiros Barreto H, Cerqueira Fontinele F, Pereira De Oliveira A, Arcanjo DDR, Cavalcanti Dos Santos BH, De Abreu APL, et al. Phytochemical prospection and modulation of antibiotic activity in vitro by lippia origanoides H.B.K. in methicillin resistant staphylococcus aureus. Biomed Res Int. 2014;2014. Available from: http://doi.org/10.1155/2014/305610

90. Sousa EO, Barreto FS. Atividade antibacteriana e interferência de Lantana camara L. e Lantana montevidensis (Spreng.) Briq. na resistência de aminoglicosídeos. Rev Bras Biociências. 2011;9(1):1–5. Available from: ISSN 1980-4849

91. Matias EEF, Santos KKA, Almeida TS, Costa JGM, Coutinho HDM. Enhancement of Antibiotic Activity by Cordia verbenacea DC. Lat Am J Pharm. 2010;29:1049–52.

92. Lee JY, Kim YS, Shin DH. Antimicrobial synergistic effect of linolenic acid and monoglyceride against Bacillus cereus and Staphylococcus aureus. J Agric Food Chem. 2002;50(7):2193–9. Available from: http://doi.org/10.1021/jf011175a

93. Chu-Kung AF, Bozzelli KN, Lockwood NA, Haseman JR, Mayo KH, Tirrell M V. Promotion of peptide antimicrobial activity by fatty acid conjugation. Bioconjug Chem. 2004;15(3):530–5. Available from: http://doi.org/10.1021/bc0341573

94. Bera S, Zhanel GG, Schweizer F. Design, synthesis, and antibacterial activities of neomycin-lipid conjugates: Polycationic lipids with potent gram-positive activity. J Med Chem. 2008;51(19):6160–4. Available from: http://doi.org/10.1021/jm800345u

95. Desbois AP, Smith VJ. Antibacterial free fatty acids: activities, mechanisms of action and biotechnological potential. Appl Microbiol Biotechnol [Internet]. 2010 Jan 15;85:1629+. Available from: https://link.gale.com/apps/doc/A229606459/AONE?u=capes&sid=bookmark-AONE&xid=82eb837a

96. Sousa EO de, Rodrigues FFG, Campos AR, Costa JGM da. <b>Phytochemical analysis and modulation in aminoglycosides antibiotics activity by *Lantana camara* L. Acta Sci Biol Sci [Internet]. 2015 Aug 6;37(2):213. Available from: http://periodicos.uem.br/ojs/index.php/ActaSciBiolSci/article/view/22877

Click here to access/download;Figure;Figura 1A.tif

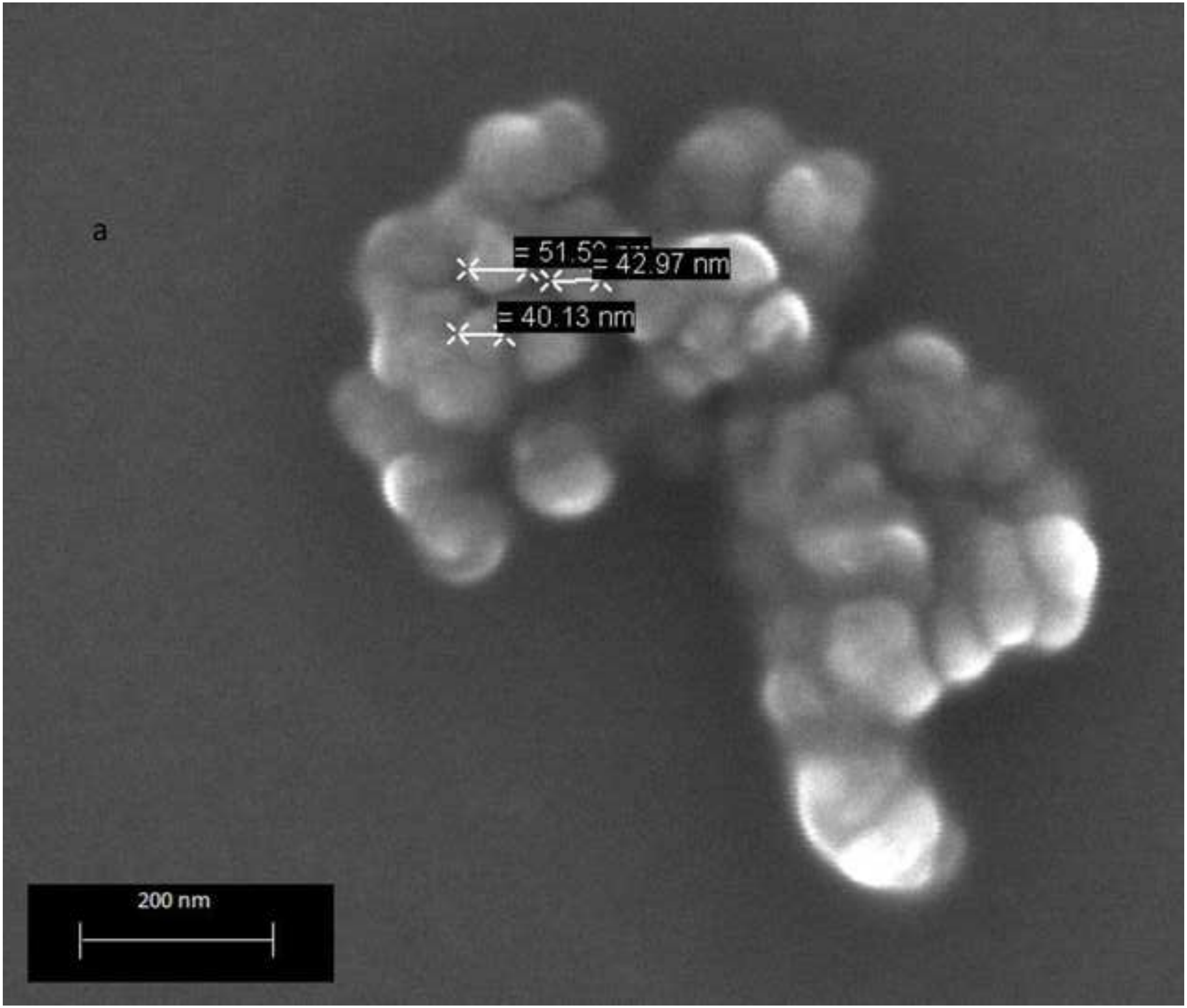

Figure

Click here to access/download;Figure;Figura 1B.tif

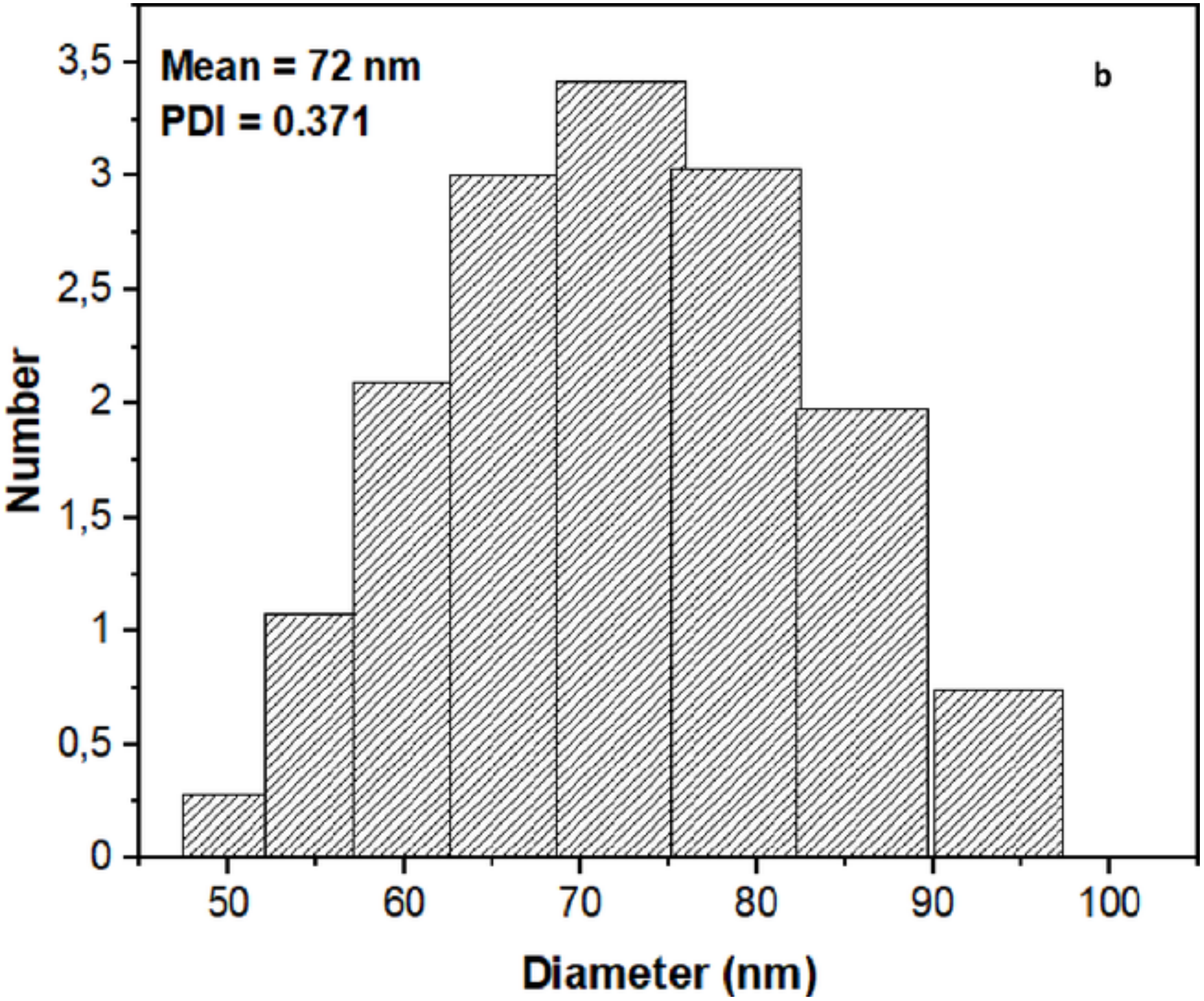

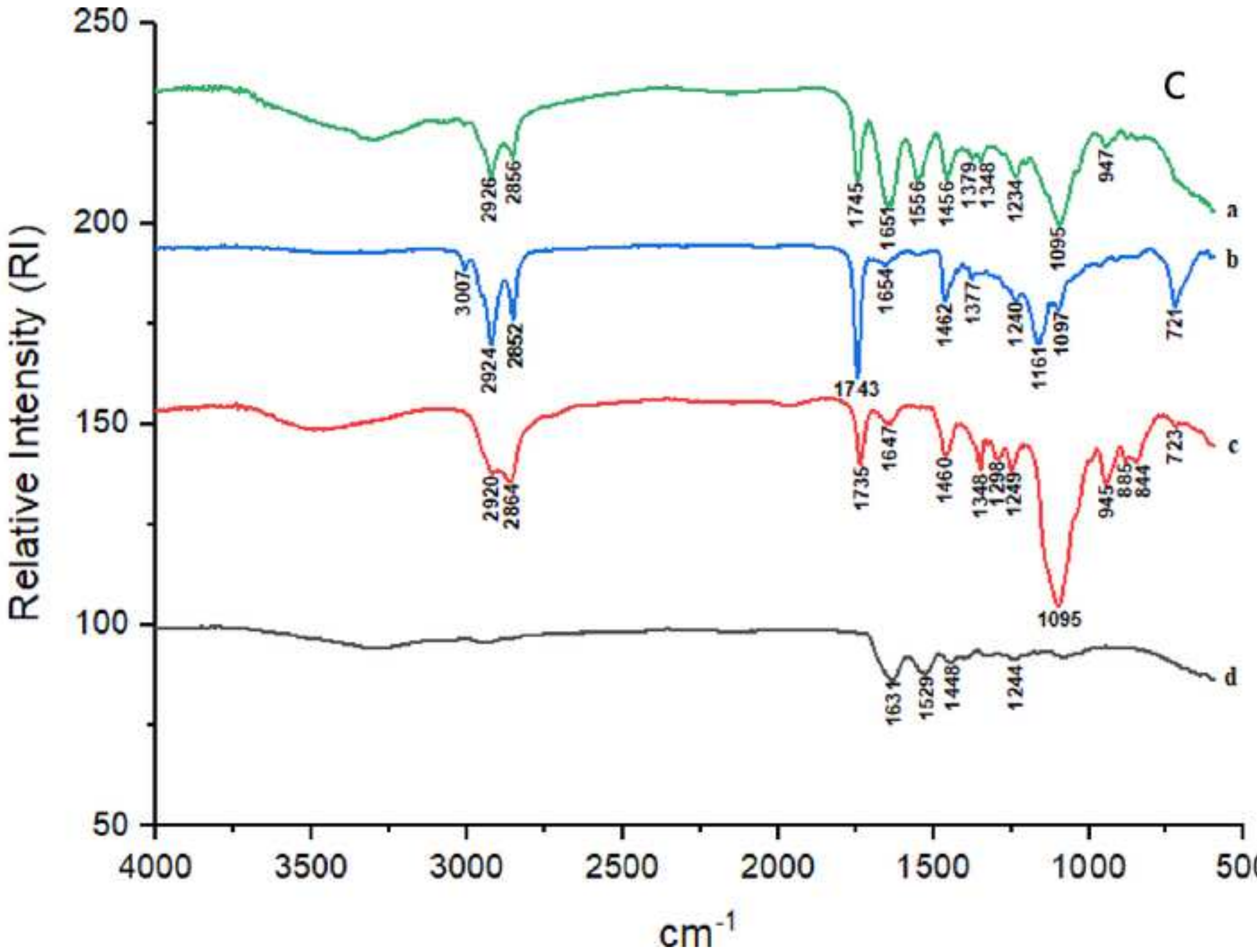

C
Relative Intensity (RI)
250
200
150
100
50
4000
3500
3000
2500
2000
1500
1000
500
cm-1
a
b
c
d
2926
2856
1745
1651
1556
1456
1379
1348
1234
1095
947
3007
2924
2852
1743
1654
1462
1377
1240
1161
1097
721
2920
2864
1735
1647
1460
1348
1298
1249
1095
945
885
844
723
1631
1529
1448
1244

 Click here to access/download;Figure;Figura 2.tif

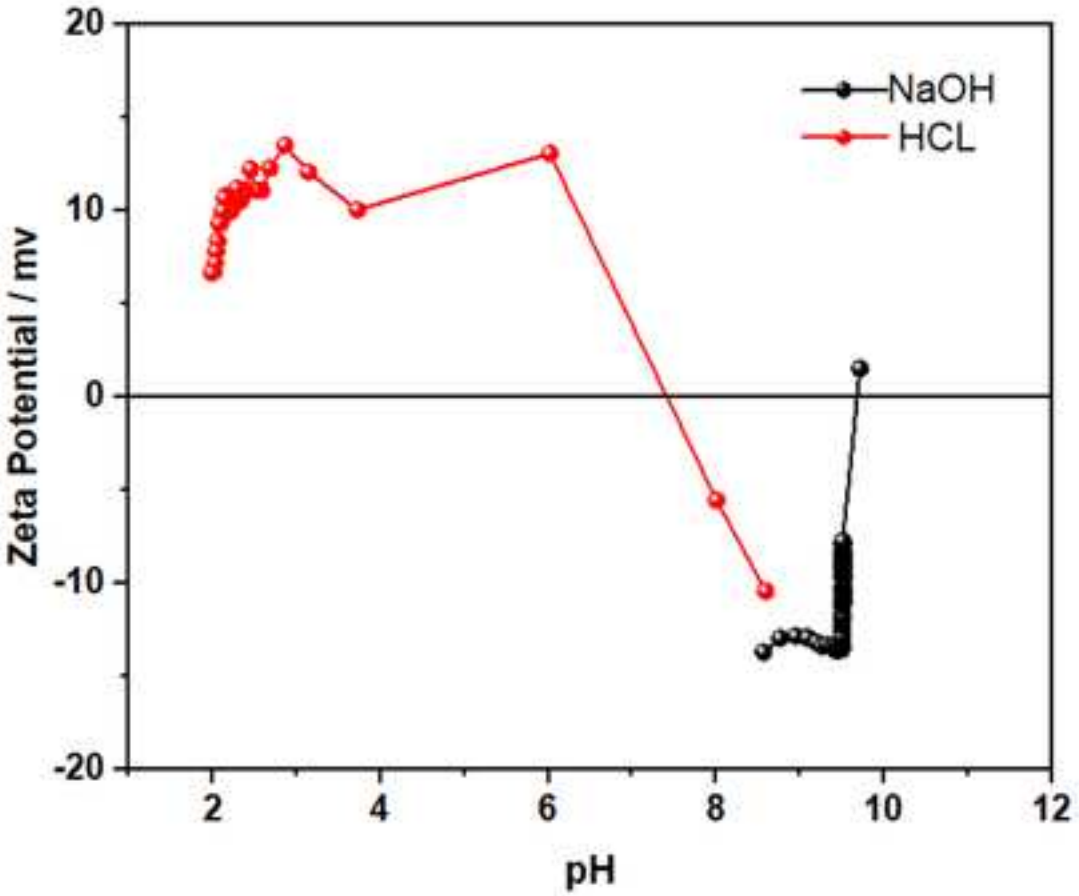

 Click here to access/download;Figure;Figure 3.tif

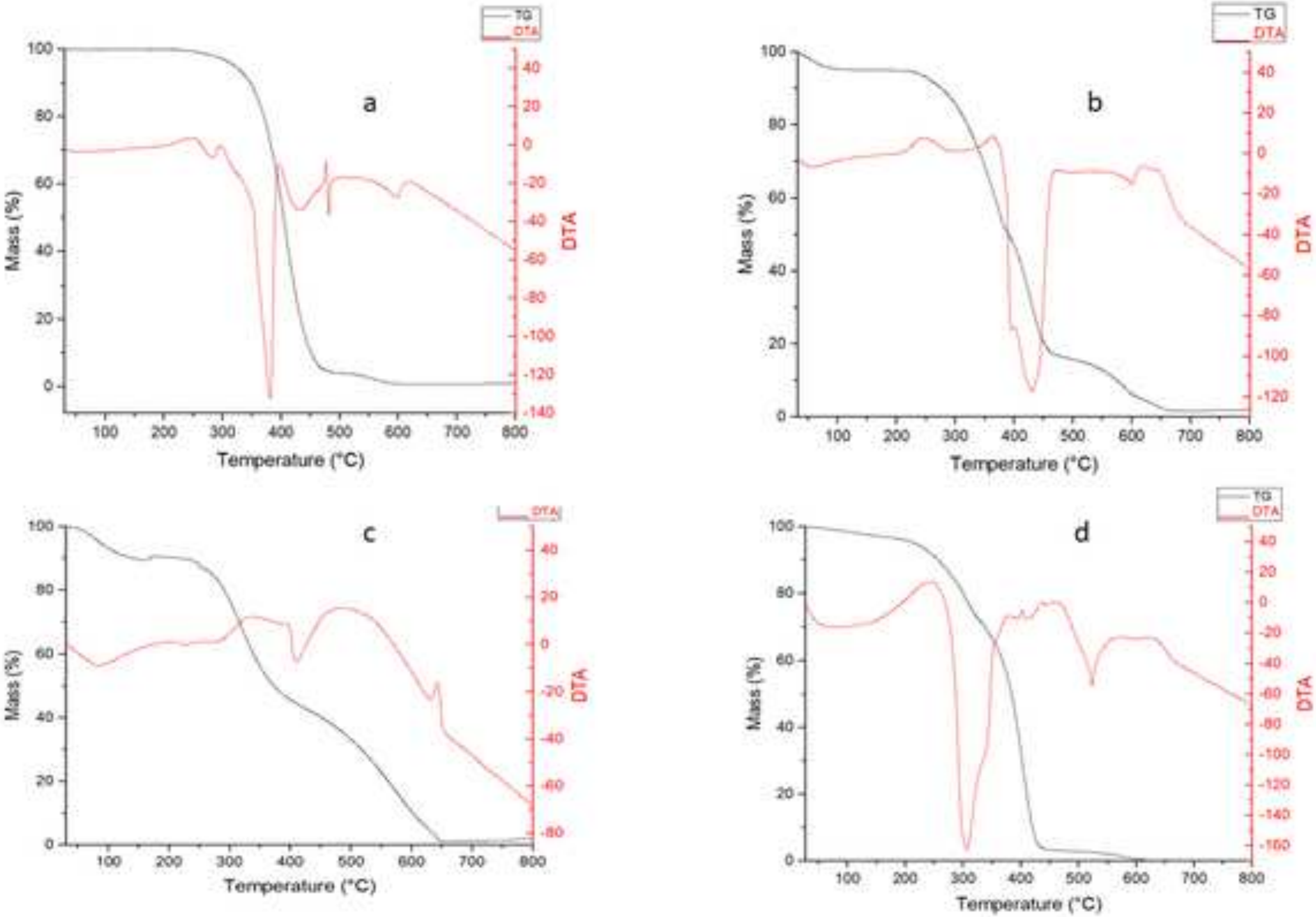

 Click here to access/download;Figure;Figure 4.tif

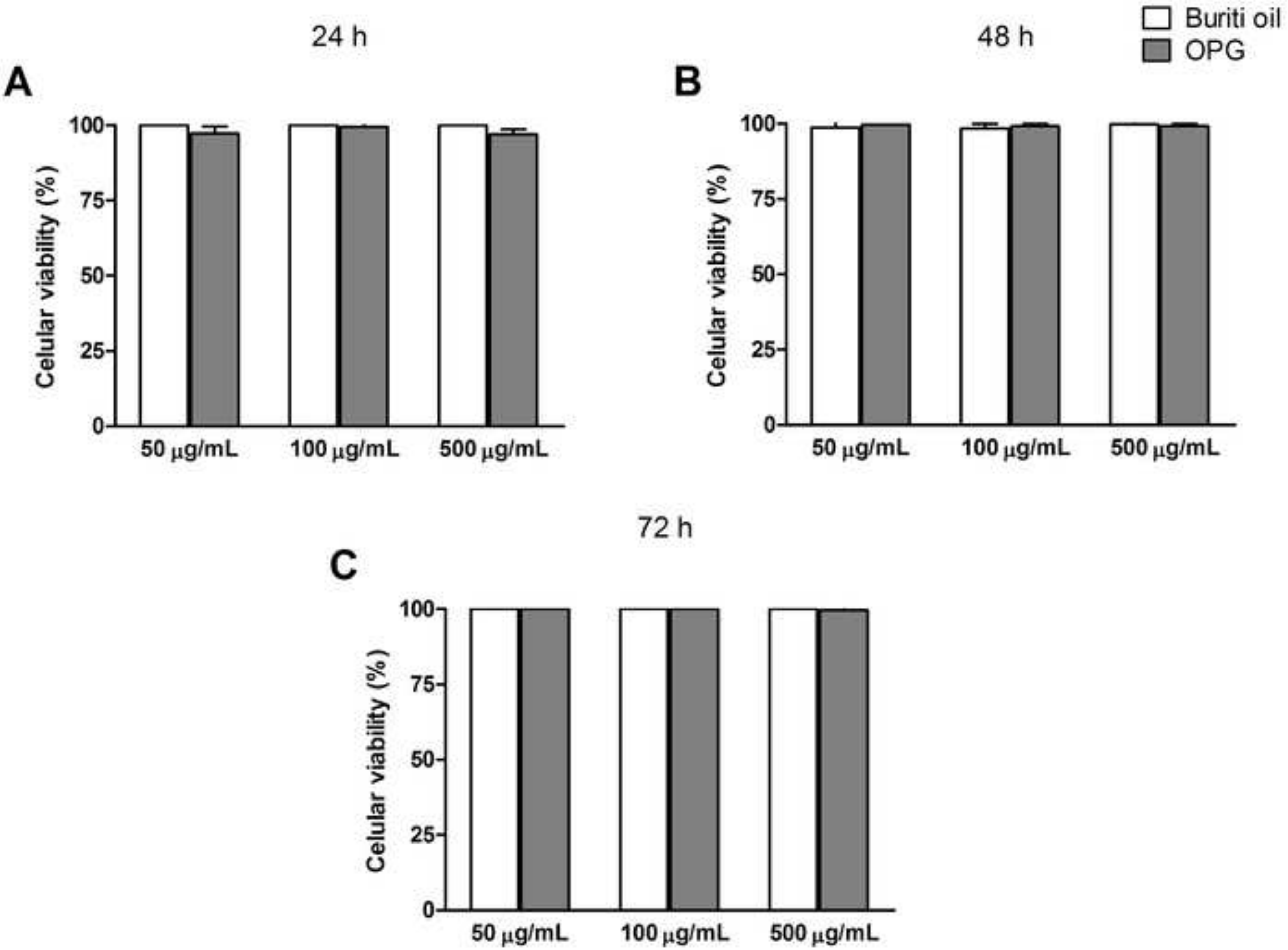

Figure Click here to access/download;Figure;Figure 5.tif

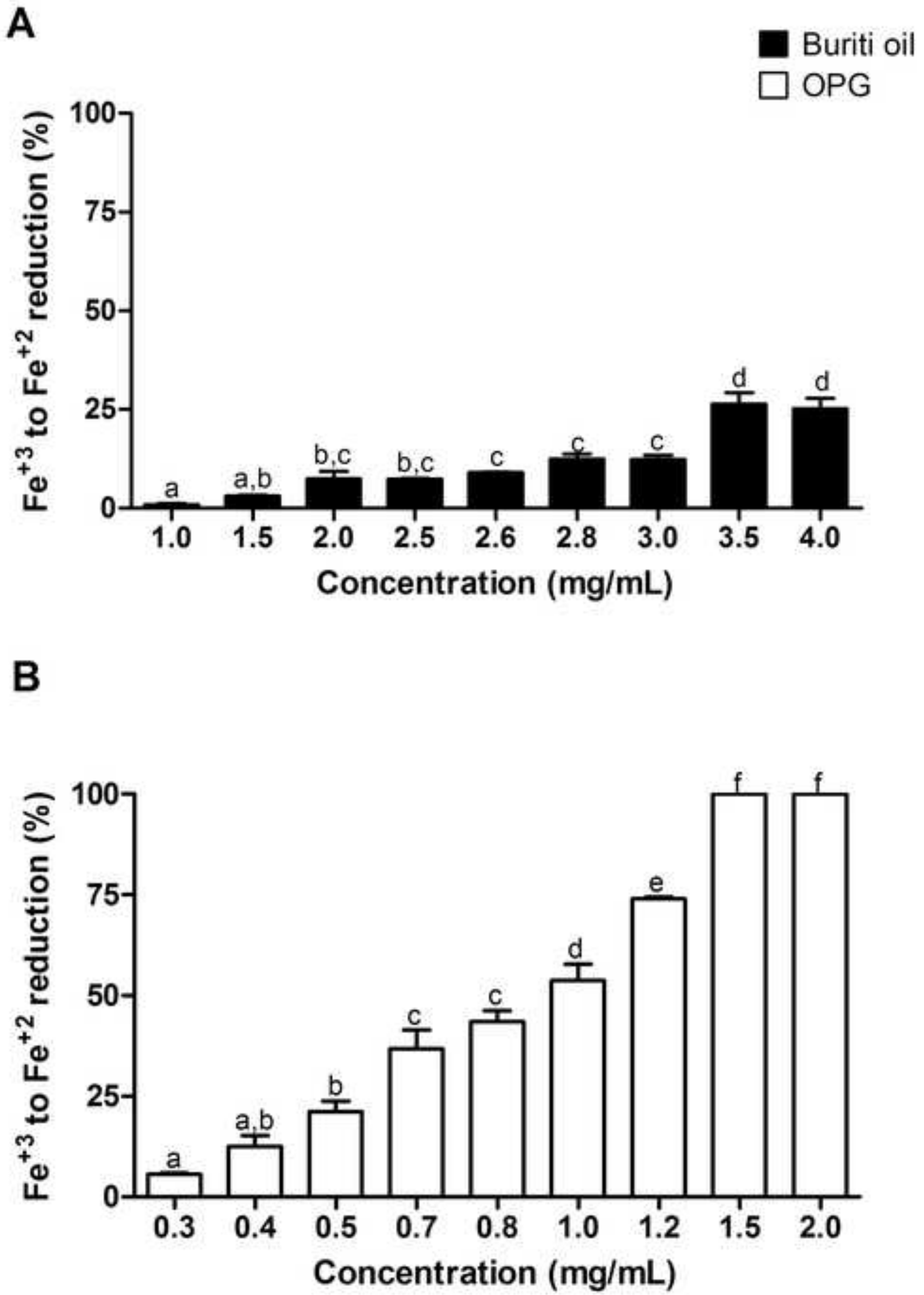

A

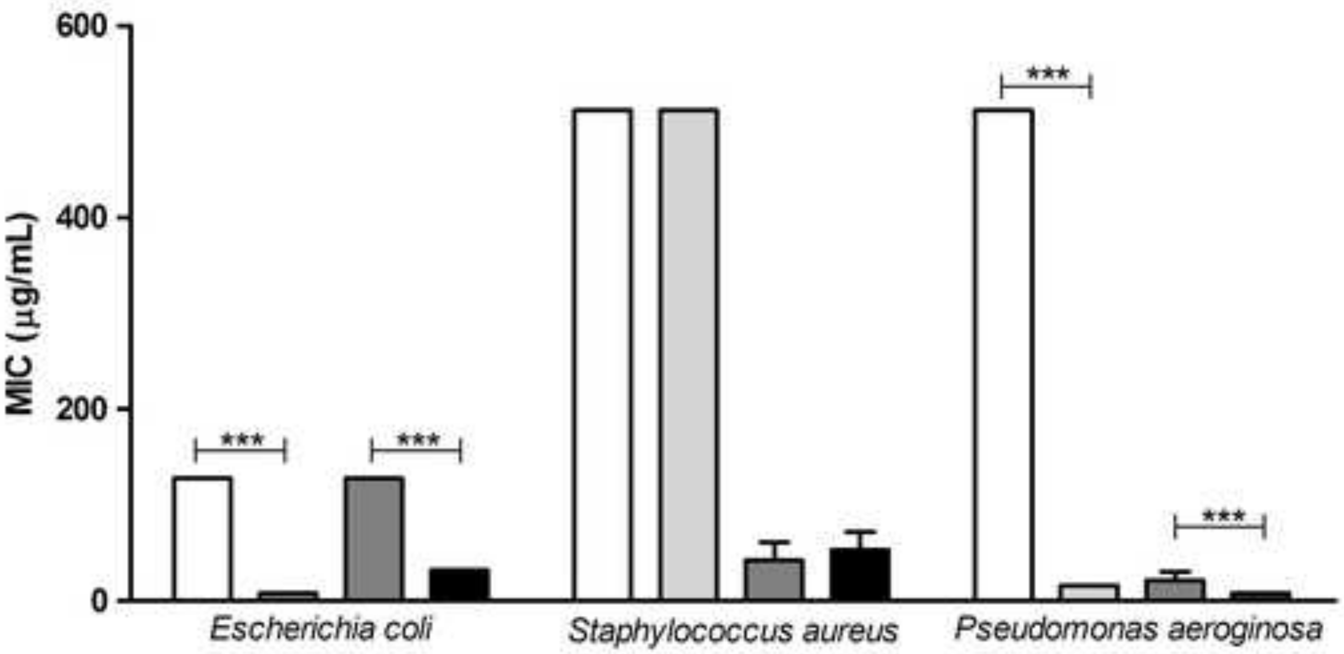
600
400
200
0
MIC (µg/mL)
***
***
***
***
Escherichia coli
Staphylococcus aureus
Pseudomonas aeroginosa

B

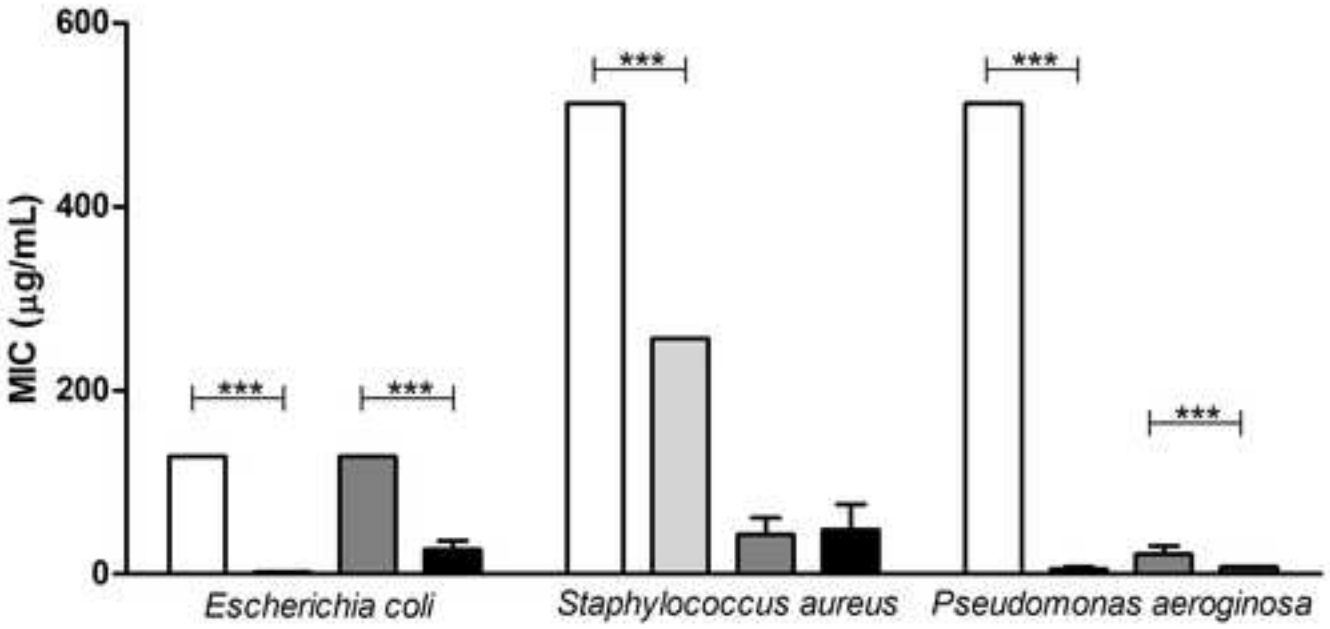
600
400
200
0
MIC (µg/mL)
***
***
***
***
***
Escherichia coli
Staphylococcus aureus
Pseudomonas aeroginosa

Click here to access/download
Supporting Information
Dataset.xlsx

Nanoencapsulation of buriti oil (*Mauritia flexuosa L.f.*) in porcine gelatin enhances the antioxidant potential and improves the effect on the antibiotic activity modulation

[1]Neyna de Santos Morais, [2]Thaís de Souza Passos, [3]Gabriela Rocha Ramos, [3]Victoria Azevedo Freire Ferreira, [4]Susana Margarida Gomes Moreira, [4]Gildácio Pereira Chaves Filho, [3]Ana Paula Gomes Barreto, [5]Pedro Ivo Palacio Leite, [5]Ray Silva de Almeida, [5]Cícera Laura Roque Paula, [6]Rafael Fernandes, [1]Sebastião Ânderson Dantas da Silva, [7]Sara Sayonara da Cruz Nascimento, [1,3]Francisco Canindé de Sousa Júnioar,[1,3]*Cristiane Fernandes de Assis

[1]Nutrition Postgraduate Program, Center for Health Sciences, Federal University of Rio Grande do Norte, Natal, RN, Brazil.

[2]Department of Nutrition, Center for Health Sciences, Federal University of Rio Grande do Norte, Natal, RN, Brazil.

[3]Department of Pharmacy, Center for Health Sciences, Federal University of Rio Grande do Norte, Natal, RN, Brazil.

[4]Department of Cellular and Molecular Biology, Biosciences Center, Federal University of Rio Grande do Norte, Natal, RN, Brazil.

[5]Department of Chemical Biology, Regional University of Cariri, Crato, CE, Brasil.

[6]Chemical Intitute, Federal University of Rio Grande do Norte, Natal, RN, Brazil.

[7]Postgraduate Program in Biotechnology - RENORBIO, Biosciences Center, Federal University of Rio Grande do Norte, Natal, RN, Brazil.

*Corresponding author: cristianeassis@hotmail.com

Abstract

The present study evaluated the cytotoxicity, antioxidant potential, and antimicrobial effect on the antibiotic activity modulation of gelatin nanoparticles containing buriti oil (OPG). The cytotoxicity analysis was performed on Chinese Hamster Ovary Cells (CHO) using a MTT [3-(4,5-dimethylthiazol-2-yl)-2,5-diphenyltetrazolium bromide] test. The antioxidant potential of buriti oil and OPG was determined by total antioxidant capacity, reducing power, and the ABTS *(2,2'-azinobis-3-ethylbenzothiazoline-6-sulfonic acid)* test. The modulating antimicrobial activity was evaluated by determining the minimum inhibitory concentration (MIC) concentration against *Escherichia coli, Pseudomonas aeruginosa, Staphylococcus aureus,* gentamicin and norflaxacillin. The nanoformulation of OPG did not show a cytotoxic effect on CHO cells and had a higher antioxidant potential than free buriti oil ($p<0.05$). The combination of antibiotics with free buriti oil and OPG was more efficient in inhibiting *E. coli* and *P. aeruginosa* than isolated norfloxacillin and gentamicin ($p<0.05$). Regarding the inhibition of *S. aureus*, OPG in combination with norfloxacillin reduced MIC by 50%. Nanoencapsulation was a viable alternative to enhance functionality and adding commercial value to buriti oil.

Introduction

Mauritia palm or buriti (*Mauritia flexuosa* L.f.) is an abundant palm tree in South America, predominant in extensive areas of Brazil and native to areas of the Amazon Forest and Cerrado biome(1). Buriti fruit is considered as a functional food due to its high levels of carotenoids. The pulp is regarded as one of the primary sources of carotenoids found in Brazilian flora diversity, with emphasis on β carotene, which has provitamin activity A(2). In addition to containing minerals (calcium, zinc, sodium), fibers, proteins, and unsaturated fatty acids(3).

Buriti oil can be obtained from the fruit by extracting the pulp and/or pulp and peel by conventional methods (mechanical and chemical extraction)(4–6), capable of influencing the product characteristics. It is of great interest in the cosmetic and food product industries because it has a wide and varied application due to its chemical composition(7).

The oil contains vitamin E considered an important antioxidant capable of interrupting the action of free radicals and protecting the cytoplasmic membranes from oxidation, reducing pre-cancerous lesions(8–10). In addition, it also has unsaturated fatty acids, which are associated with reduced levels of triglycerides and total cholesterol, consequently constituting a protective factor for cardiovascular diseases(11) and a protective effect on platelet activation and thrombosis(12). It also has ascorbic acid (vitamin C), which helps form bone and teeth and acts on the immune system, preventing flu, diabetes, and scurvy(13).

Furthermore, vegetable oils have a potential effect against microorganisms such as bacteria(5). Thus, the search for new compounds with antibacterial activity has been the objective of some research to solve the problem associated with bacterial resistance, which has become common in recent decades, increasing the concern of health authorities(14,15). In this context, natural products have been an essential tool for being a source of new compounds with antibacterial properties, which can modulate and increase the activity and efficiency of conventional antibiotics or be resistance modifying agents(15).

Thus, it is well-known that buriti oil has numerous biological properties(16). However, bioactive compounds are susceptible to degradation due to sensitivity to processing and storage factors such as temperature, pH, presence of light, and oxygen (17,18). The oil's oxidation process causes unpleasant tastes, loses nutrients, and produces many toxic compounds

(hydroperoxides and aldehydes) that cause mutation, aging, stroke, emphysema, heart disease, and cancer(19). Another technological obstacle associated with the use of buriti oil in foods is associated with its lipophilic nature, which makes it challenging to apply in products with aqueous matrices, limiting the development of new products(20).

In this context, nanoencapsulation is a strategy that can be used to protect substances against environmental factors (heat, light, oxygen, humidity), improve stability, acceptability, and handling conditions, restrict contact with other components and control the release and delivery of bioactives to the target site. In addition, reducing the size to the nanometric scale (< 100 nm) facilitates dispersion of lipophilic substances in an aqueous matrix due to the increase in the contact surface. It can also preserve or enhance the biological properties of bioactive compounds(19–22). Thus, nanoencapsulation can increase the antioxidant and antimicrobial potential, among other functionalities, reducing the concentration of the bioactive of interest necessary to achieve the desired effect (23).

Considering the properties present in vegetable oils, especially buriti oil, and the current needs of the food and pharmaceutical industry, our research group has been developing studies with nanoencapsulated vegetable oils (20,22). Castro et al. (20) investigated the nanoencapsulation of buriti oil using different encapsulating agents, namely porcine gelatin (OPG) and the combination of sodium alginate and porcine gelatin (OAG), to evaluate the effect on water dispersibility and antimicrobial activity. The results showed that OPG presented the smallest particle size [51.0 (6.07) nm] and the best chemical interaction between the materials present in the system. As a result, OPG showed greater dispersibility in water [85.62% (7.82)] and potentiated the antimicrobial activity of buriti oil in 59%, 62%, and 43% against the bacteria *Pseudomonas aeruginosa*, *Klebsiella pneumonia,* and *Staphylococcus aureus*, respectively.

Therefore, due to the potential of buriti oil both from an economic and biotechnological point of view and the results obtained by Castro et al. (20), it is of fundamental importance to deepen the knowledge regarding the effects of nanoencapsulation on the biological properties of buriti oil. The resistance of microorganisms to antibiotics has increased interest in research that seeks new drugs with antimicrobial potential or that act by modulating the activity of existing antibiotics. This study aimed to assess the cytotoxicity, antioxidant potential, and modulating action on antibiotics of nanoparticles based on porcine gelatin containing buriti oil (OPG).

Materials and Methods

Materials

Buriti oil from the *Mauritia flexuosa* species was supplied by the Plantus® S.A. cosmetics company (Nísia Floresta, in the Rio Grande do Norte, Brazil) with an IBD certificate (Agricultural and Food Inspections and Certifications). The samples were transported under protection from light and refrigerated to the Bromatology Laboratory of the Department of Pharmacy at the Federal University of Rio Grande do Norte (UFRN), where they were stored at 4 ºC.

Porcine gelatin (Type A), the surfactant Tween 20, 2,2'-azinobis (3-ethylbenzothiazoline-6-sulfonic acid) (ABTS), and resazurin obtained from Sigma-Aldrich®.

Microorganisms

Multiresistant strains of *Staphylococcus aureus* 10, *Pseudomonas aeruginosa* 24, and *Escherichia coli* 06 were supplied by the Laboratory of Microbiology and Molecular Biology of the Regional University of Cariri - URCA (Crato - CE, Brazil). The Chinese hamster ovary cell line (CHO-K1 cells) was purchased from ATCC® CCL-61™ (American Type Culture Collection), and Dulbecco's Modified Eagle Culture Medium (DMEM; Gibco, Gaithersbug, MD, USA) was used, while 3-4,5-Dimethyl-thiazol-2-yl-2,5-diphenyltetrazolium bromide (MTT) was purchased from Invitrogen (MTT; Invitrogen, Oregon, USA).

Methods

Obtaining the nanoformulation containing buriti oil

The nanoformulation based on porcine gelatine containing buriti oil (OPG) was obtained through the technique of O/W emulsification followed by dispersion of a solution containing encapsulating agent in the obtained emulsion based on Medeiros et al.(24) with modifications proposed by Castro et al.(20). Porcine gelatin (Sigma®) and Tween 20 (Sigma®) were used as encapsulating agent and surfactant, respectively. After the encapsulation process, the resulting emulsion was subjected to drying by lyophilization (LioTop L101) at -57 ºC and pressure of 43 µHg.

Characterization of the obtained nanoparticles

The new batch of OPG obtained for this study was characterized to determine its morphology, particle diameter, and chemical interactions by Scanning Electron Microscopy, Dynamic Light Scattering, and Fourier Transform Infrared Spectroscopy, respectively, according to Castro et al.(20). Furthermore, it was also evaluated concerning the distribution of charges on the surface of the particles and in terms of thermal resistance by Zeta Potential, Thermogravimetry, and Differential Thermal Analysis, respectively.

Scanning electron microscopy (SEM)

OPG was suspended in acetone, and the dispersion was dropped onto silicon plates attached to stubs using carbon tape. It was subsequently analyzed at different magnifications in high vacuum at 2-3 kV and without metallization using a FEG-SEM ZEISS microscope (AURIGA).

Dynamic light scattering (DLS)

The nanoformulation was subjected to crosslinking with glutaraldehyde to measure the diameter of the particles, according to Castro et al.(20), to promote particle deagglomeration. It was subsequently dispersed using an ultrasonic bath in DMSO (dimethylsulfoxide - 1 $mg.mL^{-1}$). Measurements were performed in triplicate (60 s each) using only 2 mL of suspension. Data were analyzed using the NANO-flex Control 0.9.7 software program. The entire experiment was carried out in triplicate. The data obtained referring to the mean and standard deviation were plotted in the Origin® 8 program to obtain the histogram with the particle size distribution.

Fourier transform infrared spectroscopy (FTIR)

Buriti oil, porcine gelatin, Tween 20, and OPG were homogenized separately with potassium bromide (KBr). Then they were macerated and pressed to obtain pellets. The spectra were recorded in transmittance with the mid-infrared region (400 to 4000 cm^{-1}). A Shimadzu spectrometer (FTIR-8400S, IRAFFINITY-1 series, IR SOLUTION version 1.60 software) was used with a scan number of 32 and resolution of 4 cm^{-1}.

Zeta Potential

The Zeta Potential measurements of the OPG nanoformulation were determined using a STABINO II Particle charge Titration device (Colloid Metrix). First, 10 mg of the sample was

diluted in 10 mL of ultrapure water (≥ 18 MΩ cm^{-1}) and then transferred to the cylindrical Teflon cell. The zeta potential measurement with pH variation (similar to a titration) consisted of individually adding aliquots (10 μL) of a strong acid (HCl - 0.1 M) or a strong base (NaOH – 0.025 M).

Thermogravimetry (tg) and differential thermal analysis (DTA)

We employed thermoanalytical analyzes to evaluate the buriti oil, Tween 20, porcine gelatin, and OPG nanoformulation. Thus, from 6 to 7 mg of each material were evaluated in a Shimadzu® DTG-60 thermal analyzer with a heating rate of 10 $°C.min^{-1}$ in the temperature range of 30 °C to 800 °C in a nitrogen atmosphere with a flow of 50 $mL.min^{-1}$.

It is noteworthy that the variations in the residual mass of the materials concerning the variation in time and temperature were provided by the equipment. The Origin Pro Graphing & Analysis version 9.8.0.200 software program from OriginLab® was used to build the TG and DTA graphs.

Incorporation efficiency (%)

The incorporation efficiency was determined based on El-Messery et al.(25) with modifications. 15 mL of hexane in 1.5 g of the nanoformulation were added under agitation in a TE-421 rotary incubator (Tecnal®) for 2 min, and then the mixture was filtered on Whatman No.1 filter paper.

Next, the content retained on the filter paper was washed twice with 20 mL of hexane. The liquid phase was collected, and the hexane was removed in an incubator at 60 °C for 48 h. The remaining extract represents the unencapsulated oil present on the surface of the encapsulates. The amount of nanoencapsulated oil was obtained through the difference between the initial amount of oil used to promote the nanoencapsulation process and the free oil present on the surface of the nanoparticles after the hexane evaporates, as described below (Equation 1). The average amount of OPG obtained from the triplicate produced and measured with the aid of an analytical balance was considered (Edutec, EEQ9003F-B).

$$EO\ (g) = OUE - RO \quad Equation\ (1)$$

EO: Quantity of encapsulated oil; OUE: Weight of oil used in encapsulation; RO: Weight of remaining oil.

The encapsulation incorporation was calculated using Equation 2, described below according to El-Messery et al.(25), considering the average amount of OPG obtained from the triplicate produced and measured using an analytical scale (Edutec, EEQ9003F-B).

$$EI\ (\%) = \left[\frac{EO}{IO}\right] x100\ Equation\ (2)$$

EI: Encapsulation incorporation; EO: Amount of encapsulated oil; IO: Initial oil quantity

Cytotoxicity evaluation

MTT assay

Chinese hamster ovary (CHO) cells were seeded in 96-well plates at a density of $2x10^3$ cells.mL^{-1} and cultured in α-MEM medium supplemented with 10% FCS (fetal bovine serum), 1% antibiotic and antimycotic solution, and 1% glutamine (basal medium).

At the end of 24 hours, the medium was changed with the medium containing the buriti oil and OPG samples at concentrations 50, 100, and 500 µg.mL^{-1}, and cytotoxicity was evaluated after 24, 48, and 72 hours. Cells maintained in the basal medium were used as a negative control. At the end of each time, 1 mg.mL^{-1} of MTT solubilized in PBS buffer was added, the supernatant was aspirated after incubation at 37 °C for 4 hours, and the formazan crystals solubilized with 200 µl of DMSO. Absorbance was measured at 570 nm in a plate reader (BioTek, µQuant model), and cell viability was calculated according to Equation 3 described below.

$$MTT\ reduction(\%) = \frac{Abs\ treatment}{Abs\ negative\ control}\ x\ 100\ Equation\ (3)$$

Antioxidant activity

Total antioxidant capacity (TAC)

The method proposed by Prieto et al(26). was used to determine TAC (Total antioxidant capacity). In each test tube, 100 µL of 40 mM sulfuric acid-ammonium molybdate, 280 mM sodium phosphate, and 100 µL of the sample solutions were added at a concentration of 2 mg.mL^{-1} and then 700 µL of distilled water in the test tubes.

Then, the tubes were shaken and incubated in a water bath (QUIMIS, Mod. Q334M-28) at 90 ºC for 90 minutes to subsequently take the absorbance reading by spectrophotometry (Biospectrum, Mod. SP-220) at 695 nm. The experiment was carried out in triplicate using solutions containing the encapsulating agents as a control. Antioxidant activity was expressed in milligrams of ascorbic acid per gram of sample (mg AA.g of sample^{-1}). The standard curve was constructed using different ascorbic acid concentrations (25 -250 mg.g^{-1}).

Reducing power test

The reducing power of the samples was quantified as described by Wang et al.(27). First, 4 mL of a reaction mixture containing different buriti oil sample (1-50 mg.mL^{-1}) and OPG (3-20 mg.mL^{-1}) concentrations in phosphate buffer (0.2 M, pH 6.6) were incubated with potassium ferrocyanide (1% m/v) at 50 ºC for 20 minutes. The reaction was terminated by a TCA solution (10% m/v). The solution was then mixed with distilled water and iron chloride (0.1% m/v), and the absorbance was measured at 700 nm. The control was carried out with the encapsulating agent. The result was expressed in the reduction of Fe^{+3} to Fe^{+2} (%).

Antioxidant activity by 2,2'-azinobis-3-ethylbenzothiazoline-6-sulfonic acid (ABTS•)

The ability to scavenge the 2,2'-azinobis-3-ethylbenzothiazoline-6-sulfonic acid (ABTS•) radical cation was determined by adapting the methodology described by Rufino et al.(28). The ABTS• solution was prepared by adding ABTS• (7 mM) and potassium persulfate (2.45 mM) and incubated at room temperature in the dark for 16 h. Next, the ABTS solution (1 mL) was diluted in DMSO to obtain an absorbance of 0.8, at a wavelength of 734 nm (29,30). Finally, 200 µL of this ABTS• radical solution was carefully added to the microplate well together with 40 µL of the evaluated samples.

The control was prepared to contain 200 µL of ABTS and 40 µL of DMSO. The absorbance reading at 734 nm was performed in a microplate reader (Loccus – Model LMR FLEX). After reading the absorbances, the antioxidant activity (AA) percentage was estimated using Equation 4 described below.

$$ABTS\ radical\ inhibition = 100\ X\ \frac{Abs\ control - Abs\ sample}{Abs\ control} \quad Equ[illegible]o\ (4)$$

The linear regression curve was determined to calculate the concentration required for the samples (crude oil and OPG) to inhibit 50% of the ABTS radical (IC 50).

Determination of the modulating antimicrobial activity of antibiotics

The antimicrobial activity test was performed by determining the Minimum Inhibitory Concentration (MIC) of antibiotics (norfloxacin and gentamycin) and modulating activity using buriti oil and OPG (dilution of in DMSO, in the concentration of 1024 $\mu g.mL^{-1}$).

The bacteria used were multiresistant *Pseudomonas auriginosa* 24, *Staphylococcus aureus* 10, and *Escherichia coli* 06 strains collected in exams provided by the Regional University of Cariri (Juazeiro do Norte/CE). The origin and resistance profile of these strains were described by Bezerra et al.(31).

Bacteria cultures of 10^7 $CFU.mL^{-1}$ kept in agar were subcultured in brain-heart broth (Brain Heart Infusion - BHI) at 37 ºC for 24 h. Then, the distribution medium was serially prepared at 1:10, and 100 µL were added into Eppendorfs of BHI medium in 900 µL of inoculum. This distribution medium was transferred to the microplate, 100 µL in each well. Norfloxacin and gentamycin antibiotics were added to the test, and their inhibitory effects were investigated in their combinations with buriti oil and OPG. Both concentrations of antibiotics, crude buriti oil and OPG, were fixed at 1024 $\mu g.mL^{-1}$ with a dilution of 100 µL in octuplicate. Controls were included in the trials, namely the medium and the inoculum, the porcine gelatin, and the Triton X-100. The filled plates were incubated at 35 (±2)ºC for 24 hours(32).

Next, an indicator solution of sodium resazurin was prepared in sterile distilled water at a concentration of 0.01% (m/v) to evidence the MIC of the samples. After incubation, 20 µL of the indicator solution was added to each well and the plates underwent an incubation period of 1 hour at room temperature. A change from blue to pink color due to the reduction of resazurin indicated bacterial growth at 37 ºC (32). MIC was defined as the lowest concentration capable of inhibiting microbial growth, as evidenced by the unaltered blue color.

According to the equations 4,5,6 below, the fractional inhibitory concentration index (FICi) measurement was determined to demonstrate the synergistic effect. The FICi index was interpreted according to the European Committee for Antimicrobial Susceptibility Testing (30), considering the synergistic effect FICi ≤ 0.5 (33).

$$FIC\ buriti\ oil\ or\ OPG = \frac{MIC\ of\ buriti\ oil\ \ or\ OPG\ in\ combination}{MIC\ \ of\ buriti\ oil}\ Equation\ (4)$$

$$FIC\ antibiotic = \frac{MIC\ of\ antibiotic\ in\ combination}{MIC\ of\ antibiotic}\ Equation\ (5)$$

$$FIC\ index\ (FICi) = \ (FIC\ oil\ buriti\ or\ OPG + FIC\ antibiotic)\ Equation\ (6)$$

Statistical analysis

Statistical analysis was performed using Graph Pad Prism version 5.0 software. Results were expressed as mean and standard deviation. First, the data obtained for the analyzes related to antioxidant potential and antimicrobial activity and modulation of antibiotic action were evaluated for normality using the Shapiro-Wilk test. Data were evaluated using the Student's t-test to determine the antioxidant potential through CAT and ABTS tests and assess antimicrobial activity and cytotoxicity. An analysis of variance and Tukey's post-test were used for the reducing power. A value of $p<0.05$ was considered statistically significant in all cases.

Results and Discussion

Characterization of porcine gelatin-based nanoparticles containing buriti oil (OPG)

The micrographs obtained through Scanning Electron Microscopy (SEM) analysis (Fig 1A) showed particles with a spherical shape, smooth surface without cracks and physical size at the nanometer scale (<100 nm), demonstrating good protection of the core through the encapsulation. The results obtained by Castro et al.(20) for buriti oil nanoencapsulated in porcine gelatin (OPG) were similar. Lira et al.(22) showed the same characteristics observed for shape and surface in all formulations containing quinoa oil.

Figure 1. Characterization of buriti oil nanoencapsulated in porcine gelatin. A. Micrograph obtained by Scanning Electron Microscopy. B. Dynamic light scattering. C. Fourier Transform Infrared Spectroscopy of nanoencapsulated buriti oil (a), buriti oil (b), Tween 20 (c), porcine gelatin (d).

The data obtained for particle diameter using DLS (Fig 1B) showed mean diameter and polydispersion index for OPG equal 72.0(0.68) and 0.371(0.0251), respectively. Thus, it is possible to state that OPG presented unimodal size distribution and reinforced the physical

diameter observed by SEM, confirming that it is a nanoparticle. The nanometric particle size enables the food industry to improve the bioavailability of poorly soluble substances, such as lipids and natural antioxidants(34).

Castro et al.(20) found values of 51.00 (6.07) nm and 0.40 (0.05) for particle size and polydispersion index for OPG, respectively, constituting values close to those found in this work. Particles with a large diameter and size distribution can affect the texture and compromise the incorporated bioactive compounds, which did not occur in this study. The polydispersion index of less than 1 indicates homogeneity in the analyzed particle(20,35). Thus, a particle that presents itself more homogeneously is inserted in a product more efficiently, not affecting the texture, therefore being positive for consumer use(35,36).

Fig 1C shows the FTIR spectra obtained for nanoencapsulated buriti oil, buriti oil, Tween 20, porcine gelatin. Hydrocarbon groups were detected in the buriti oil spectrum (Fig 1C, line b) by vibrational bands in the range of 2924 - 2852 cm^{-1} (C-H), in addition to bands in the region of 3007 cm^{-1} and 1462 cm^{-1}, which respectively characterize the –OH and $-CH_3$ groups, meaning that they reflect the strong constitution of this oil described in the literature as a source ingredient of carotenoids [16]. The stretching of the vibrational band at 1743 cm^{-1} indicates the presence of double bonds in the oil chain (C = C; C = O), which shows the content of unsaturated fatty acids(37).

The vibrational band at 1651 cm^{-1} in the porcine gelatin spectrum (Fig 1C, line d) reflects the presence of the C=O bond (amide I), according to Silverstein & Webster(37). Another band is observed at 1529 cm-1, indicating amide (N-H)(38).

The spectrum of the Tween 20 surfactant (Fig 1C, line c) presented vibrations at 2920 cm^{-1} and 2864 cm^{-1} referring to stretching of the asymmetric and symmetrical methylene vibrations; at 1735 cm^{-1}, referring to the C=O connection; and at 1095 cm^{-1}, referring to the vibration stretching of $-CH_2-O-CH_2-$(37).

When observing the spectrum obtained for OPG (Fig 1C, line a), it can be seen that there was an interaction between porcine gelatin, buriti oil, and Tween 20 surfactant due to the attenuation and/or displacement of the vibrational bands that characterize the presence of the buriti oil (3007, 2924, 2852 and 1743 cm^{-1}), indicating protection of the oil in the obtained particles. The formation of new bands was also observed, which were not observed in the spectra of the

raw materials (1556, 1348, and 1097 cm^{-1}), which may indicate hydrophobic interactions between buriti oil and the non-polar amino acids of porcine gelatin. Castro et al.(20) also observed chemical interactions between OPG constituents, meaning buriti oil with porcine gelatin.

The encapsulation efficiency determines the oil content (%) that was successfully encapsulated inside the particles, also being an indicator of free oil present on the surface of the particles(39). The nanoformulation yield was 13.65g (1.14), and the result of the encapsulation efficiency was 89.56% (1.14). According to the literature is considered high efficiency, or when ≥ 80%(40,41).

Thus, this analysis confirmed the results obtained through the FTIR, which indicated a chemical interaction between gelatin, buriti oil, and Tween 20, which managed to provide greater oil retention. This result can be explained by the fact that proteins have physicochemical properties which favor forming and stabilizing emulsions than carbohydrates, thus allowing greater oil retention in the particles(20,42).

It was necessary to ensure that the new batch of synthesized nanoformulation had the same physical and chemical characteristics observed in Castro. et al. (2020). Therefore, it was necessary to carry out the characterization to confirm that the diameter, morphology, chemical interactions, and incorporation efficiency were similar to the batch obtained by Castro et al. (2020), ensuring that the material's functionality would be preserved and possible to continue the study. This step is of fundamental importance to guarantee the standardization of each batch of nanoformulation obtained.

Because of these characterization results, it can be stated that the process for obtaining nanoparticles used in this study is standardized and reproducible, which is essential to maintain the OPG nanoformulation properties even when obtaining new batches. Thus, it is possible to affirm that the nanoparticles developed have the same physical and chemical characteristics as those obtained by Castro et al. (20) as desired, being possible to proceed with the study for further investigation of physical and chemical characterization, safety, antioxidant potential, and antimicrobial activity.

Zeta potential

Fig 2 shows OPG Zeta Potential values with the pH variation that resulted in a change in charge density around the nanoparticle. Low zeta potential values show a tendency to settle in

an aqueous medium. Still, this low charge indicates steric stability of OPG as they are values with charges close to zero, so they can aggregate more quickly, but they also have eased to disperse(43).

Figure 2. Zeta Potential of buriti oil nanoencapsulated in porcine gelatin under different pH conditions.

As the nanoparticle is composed of porcine gelatin and has an amphoteric character, changes in pH alter the OPG surface charge between positive and negative values(21). It is noteworthy that this change in surface charge influenced by the pH of the medium is an interesting feature when working with the application of nanoparticles in biofilms(44,45).

Thermogravimetry (TG) and differential thermal analysis (DTA)

The TG/DTA curves of crude buriti oil, OPG, porcine gelatin, and Tween 20 are shown in Fig 3.

Figure 3. Thermogravimetry graphs and differential thermal analysis. a) Porcine gelatin b) Tween 20 c) Buriti oil d) OPG.

Buriti oil presents TG mass loss onset at approximately 365 °C associated with three endothermic events in the DTA between 239 and 446ºC, which may be related to the complete breakdown of fatty acids(46). The total degradation of buriti oil ends at 596 ºC and thus presents two stages of mass loss, with a total loss of 98.87%. The thermal stability of buriti oil is high when compared to other vegetable oils such as coconut oil (257 ºC) and sesame oil (282 ºC)(46,47).

The OPG TG curve in Fig 3B shows an initial and progressive mass loss up to approximately 100 °C, corresponding to the endothermic peak in the DTA, which may indicate water evaporation in the sample(48). Then, the decomposition of the nanoparticle starts before the buriti oil at a temperature onset of 248 °C. After this event, mass loss occurs during the endothermic decomposition peak in both free buriti oil and nanoformulation. The mass loss is 95% and 83%, respectively, which may be related to the decomposition of the material wall(49). The OPG DTA curve presents an exothermic peak characteristic of the sample crystallization, as the XRD indicates that the sample is partially amorphous, confirming this event.

The TG curve of porcine gelatin in Fig 3C had an initial thermal degradation between 35 and 160 °C, which may be related to the loss of adsorbed and bound water commonly seen with

air-dried gelatin(45), confirmed by the endothermic peak in the DTA in the same temperature range. It still has the beginning of its degradation between 250 °C and 656 °C, with two stages of very evident mass loss and with a total loss of 98%. In the DTA curve, it is possible to confirm the degradation with two endothermic peaks and one exothermic peak in this same interval.

The Tween 20 TG curve in Fig 3D showed four decomposition stages. The first stage corresponds to water loss (32-196ºC) and the DTA curve presenting an endothermic peak in this temperature range, which corresponds to dehydration or desolvation. The beginning of Tween 20 degradation can be seen in the TG curve at 198 °C with the end of degradation at 654 °C, with total mass loss in the TG curve of 100%.

In evaluating the TG curves, it was possible to note that buriti oil is more stable than the other samples. However, although OPG has a lower initial degradation temperature than pure buriti oil, it is still a very high temperature for a product with a greater possibility of handling and, consequently, application than pure oil. Tween 20 and porcine gelatin may have contributed to maintaining buriti oil's stability in the form of a nanoparticle and the same amount of degradation steps. Escobar-García et al.(50) performed a thermal analysis of oil rich in free eicosapentaenoic acid (EPA) and microencapsulated in concentrated whey protein and observed that the encapsulated sample had a delay in the degradation curve than the unencapsulated sample, with this result being attributed to the protection of the encapsulating agent(51).

Table 1 shows the steps of oil decomposition, encapsulating agent, Tween 20, and nanoencapsulated. Buriti oil has two degradation stages, with the first stage between 239.09 °C - 446.21 °C with a significant mass loss of 87.45%. The initial degradation temperature is similar to the data found for buriti oil in the study of Lima et al. (231.9 °C), but it differs concerning the mass loss, which was 59.6%. The results of the studies are often contradictory, as vegetable oils are very complex because they have a complex chemical composition, including lipid oxidation products, free fatty acids, phenolic compounds, glycerides, and non-glycerides that are present in oils in different proportions(52).

Table 1: The stages (TG) and mass loss of buriti oil, porcine gelatin, Tween 20 and OPG

Sample	Number of decomposition steps	Start decomposition temperature/ °C	End decomposition temperature /°C	Mass loss (%)
Buriti oil	1	239.09	446.21	87.45
	2	446.00	596.00	11.42
Porcine gelatin	1	38.10	166.71	10.07
	2	252.69	380.57	38.66
	3	380.57	648.62	47.20
Tween 20	1	32.84	196.17	3.80
	2	198.34	313.14	20.35
	3	313.14	432.29	71.42
	4	432.29	654.29	4.70
OPG	1	36.62	107.08	4.35
	2	218.00	388.47	44.32
	3	388.47	463.93	32.93
	4	463.93	672.59	15.93

The physicochemical characteristics of buriti oils are different due to their location and other factors(53). There are disagreements among authors regarding these degradation steps. Garcia et al.(51) found only two steps for the degradation of buriti oil since the mass loss was initially high. There are some hypotheses for these differences in vegetable oils, mainly regarding their fatty acid composition. Lima et al.(54) associated the mass losses in two stages: the triglycerides' evaporation and/or pyrolysis. Garcia et al.(51) reported the first and second weight loss as the oxidation of unsaturated and saturated fatty acids, respectively. The third step was reported as the decomposition of the polymer formed during the oxidation process.

OPG showed four degradation stages, and in the first stage, there was a mass loss of 4.35% at an initial temperature of 36.62 °C to 107.08 °C. Porcine gelatin and Tween 20 present an initial degradation temperature between 38.10 °C and 32.84 °C, respectively. This data suggests that the initial degradation observed in OPG may come from the degradation of gelatin and Tween 20. The second stage of nanoformulation degradation occurs at 218 to 388.47°C, with a mass loss of less than 50%, partially corresponding to buriti oil. This data shows the OPG mass loss anticipation compared to free buriti oil, and however, the oil has a loss greater than 87%.

According to the data, it is observed that the stability of the free oil was higher in terms of initial decomposition temperature. Still, its degradation is very drastic, while the nanoformulation loses mass more slowly. It is important to emphasize that OPG is more stable than porcine gelatin and Tween 2 0 and that it has stability approaching that of pure buriti oil. In a way, OPG has significant advantages as it is a product with better handling, is more innovative, and maintains high oil stability. These data are important for the food area since nanoencapsulation exerts the possibility of using the product at different temperatures.

Table 2 presents the data of the DTA curves for buriti oil, encapsulating agent, Tween 20, and nanoencapsulated. It is possible to observe that all samples showed four endothermic or exothermic events characteristic of the analyzed samples. DTA can accurately measure the enthalpic transition temperatures of energy absorption and release. In this case, it is observed that the buriti oil and the nanoencapsulated present four characteristic endothermic events of their decomposition. The first endothermic decomposition event in buriti oil occurs at 259.03 °C with a maximum peak of 282.98 °C (ΔH = 115.86). In comparison, this event seems to happen at a lower temperature of 214.05 °C in OPG, with a maximum peak at 245.47 ºC (ΔH=286.40), corroborating the data obtained in the TG. It is also possible to visualize the endothermic peak in the nanoformulation characteristic of the dehydration of the encapsulating agent and Tween 20 around 31 and 78 ºC.

Table 2: The stages DTA of buriti oil, porcine gelatin, Tween 20, and OPG.

Sample	Stage	T_{onset}/ °C	T_{peak}/ °C	**Δ**H (J/g)
Buriti oil	1	259.03 ↓	282.98	115.86
	2	350.09 ↓	381.57	2.85x10^{6*}
	3	400.41 ↓	432.56	964.53
	4	476.98 ↓	480.94	98.64
Porcine Gelatin	1	37.69 ↓	84.81	509.39
	2	215.42 ↓	227.48	12.87
	3	399.46 ↓	409.59	370.38
	4	633.63 ↑	641.97	114.80
Tween 20	1	31.77 ↓	78.14	592.78
	2	281.13 ↓	305.44	1.90x10^{6*}
	3	397.64 ↑	402.05	6.03

	4	500.21↓	523.07	354.57
OPG	1	31.78 ↓	58.95	219.36
	2	214.05 ↓	245.47	286.40
	3	387.36 ↓	431.87	$6.18x10^{6}$*
	4	583.34 ↓	601.43	154.45

*J/Kg; ↓ Endothermic ↑ Exothermic

Cytotoxicity - MTT assay

The toxicity investigation of nanoparticles from essential oils, vegetables, and algae is necessary due to the unpredictable behavior that nanoscale materials have demonstrated(55). The International Organization for Standardization (ISO 10993) compiles several tests on biomaterial/biological tissue interaction and determines that biocompatibility starts with evaluating material from cytotoxicity(56). Part 5 of ISO 10993 is responsible for suggesting the tests and conditions necessary for evaluating the cytotoxicity test(56). According to the standard, a reduction greater than 30% in cell viability in the tests is indicative of the material's cytotoxicity.

Fig 4 shows the MTT assay result. According to the data at the tested concentrations (0.5, 2.5, and 5 $µg.mL^{-1}$), crude buriti oil and OPG were not cytotoxic to CHO cells. There is no significant difference between buriti oil and OPG. Zanatta et al.(57) evaluated the cytotoxic effect of an emulsion containing buriti oil on 3T3 cells (1000 $mg.mL^{-1}$) and found no toxic effect, corroborating the results found in this work.

Figure 4. Cell viability (%) through the MTT assay in CHO-KI cells evaluated at different times (A – 24h, B – 48h, and C – 72h) and concentrations of buriti oil and OPG.

When reduced to < 70%, the viability has cytotoxic potential, which was not seen in this study(56). Therefore, the buriti oil and the nanoformulation results are favorable because it does not present toxicity to the cells, assuming that their use is safe.

Total antioxidant capacity (TAC)

TAC is determined using the phosphomolybdenum blue complex to reduce Mo^{+6} to Mo^{+5} by antioxidant compounds and the formation of green Mo^{+5} complexes(58). The results obtained (Table 3) showed that the nanoencapsulation process increased the antioxidant capacity of buriti

oil threefold. This result can be explained by the greater dispersibility in water observed for the OPG due to the obtained particle size, suggesting an increase in the contact surface for chemical interaction with water. Besides, chemical interactions between the materials of the system, as evidenced in the FTIR, consequently increased the antioxidant activity of the encapsulate (20,22).

.

Table 3 – Total antioxidant capacity of buriti oil and OPG

Samples	mg AA. g^{-1}
Buriti oil	14.60 (1.63)^{a}
OPG	48.34 (3.71)^{b}

OPG: buriti oil nanoencapsulated in a porcine gelatin
AA: ascorbic acid
Mean and standard deviation (SD), n = 3. The different lowercase letters indicate a statistical difference.

TAC analyses for crude and encapsulated buriti oil were not found in the literature. Ribeiro et al.(59) evaluated the total antioxidant capacity of oil from faveleira seeds. The value found was 0.04 (0.00) mg AA. g^{-1}, constituting a lower result than its methanolic fraction [0.08 (0.00) mg AA. g^{-1}] and higher than its non-polar fraction [0.02 (0.00) mg AA. g^{-1}].

Silva et al.(60) extracted astaxanthin from shrimp of the *Litopenaeus vannamei* species using soybean oil and evaluated the antioxidant potential. The TAC results showed that the astaxanthin-pigmented soybean oil had higher antioxidant activity than soybean oil (control).

Zhong and Shahidi(61) compared the antioxidant activities of polar and non-polar compounds. They observed that a higher concentration of non-polar samples is needed to achieve the optimal antioxidant activity. These data corroborate this study since the buriti oil concentration required to present antioxidant activity was higher than that of OPG, which has a solubility in water.

Reducing power

The reducing power assesses the ability of a sample to donate electrons in the presence of ferric chloride under acidic conditions and thus reduce Fe^{+3} to Fe^{+2}(62). The results (Fig 5) showed that a lower OPG concentration than crude buriti oil was necessary to present similar

antioxidant activity. Therefore, it is noteworthy that there is 0.98 mg of buriti oil in 2 $mg.mL^{-1}$ of OPG according to the encapsulation efficiency and considering the amount and proportion of the encapsulating agents. It is possible to note that this concentration was sufficient to promote a reduction from Fe^{+3} to Fe^{+2} in 100%.

Figure 5. Reducing power test of crude buriti oil (A) and OPG nanoformulation. The data obtained presented parametric distribution. Therefore, the ANOVA test with Tukey's post-test was used to determine the significant differences. *Equal letters indicate that the values do not differ statistically ($p > 0.05$).

On the other hand, 1 $mg.\ mL^{-1}$ of crude buriti oil promoted a reduction from Fe^{+3} to Fe^{+2} by only 0.74%. The encapsulate concentration must take into account that there are also encapsulating agents in addition to the oil (swine gelatin and Tween 20 in a ratio of 1:2.15). In addition, encapsulation efficiency must also be considered, as not all of the oil placed in the system was encapsulated.

Based on this, the result obtained demonstrates that the nanoencapsulation process enhanced the reducing power of buriti oil, with the reduction being greater as the concentration used in the test increases. No reduction power studies were found for crude or encapsulated buriti oil. In analyzing turmeric essential oil, Mau et al.(63) observed that the reducing power increases as the concentration of oil increases, corroborating the data found in this work.

Antioxidant activity by 2,2'-azinobis-3-ethylbenzothiazoline-6-sulfonic acid (ABTS•)

Nanoencapsulation is known to increase the antioxidant potential of natural molecules(64–66). ABTS radical scavenging was evaluated using buriti oil and OPG (Table 4).

Table 4 – Antioxidant activity (IC 50) buriti oil and OPG.

**Samples**	**IC50 ($mg.\ mL^{-1}$)**
Buriti oil	4.18 (0.05)[a]
OPG	0.94 (0.02)[b]

OPG: buriti oil nanoencapsulated in a porcine gelatin
Mean and standard deviation (SD), n = 3. The different lowercase letters indicate a statistical difference.

The results obtained demonstrate that the technological process of nanoencapsulation favored the antioxidant activity since the amount of OPG used was about four times smaller than that of buriti oil to reach the IC50.

Lira et al.(22) performed an analysis for crude quinoa oil and its three nanoencapsulated formulations (encapsulated with porcine gelatin, encapsulated with whey protein, and porcine gelatin in aqueous phase 1, and encapsulated with whey protein in aqueous phase 1 and gelatin in aqueous phase 2). The IC 50 of the formulations was lower than that of the oil, corroborating the data found in this study.

Eugenol essential oil (EO) was nanoencapsulated by the O/W emulsification method with sodium caseinate, maltodextrin and Tween 80(67). The results showed that the nanoencapsulated eugenol EO had a lower IC50 for the ABTS radical assay than crude eugenol(67).

This analysis showed that the OPG nanoparticle has greater antioxidant activity than its unencapsulated form. Thus, nanoscale diameter allows for increased contact surface for chemical interactions, increased water dispersibility, bioaccessibility, and bioavailability compared to micro or macro scale size (12). Therefore, nanoencapsulated oils are protected against oxidation of their bioactive constituents and have a higher solubility in water (20,22,68). This difference is relevant for its applications in food matrices to prolong the biological action and provide health benefits (65,69).

Determination of the modulating activity of antibiotics

Due to the emergence of multidrug-resistant strains to antibiotics, combined drugs are used against these resistance mechanisms(70). Antimicrobial resistance, in general, alters antibiotic action through the following mechanisms: modification of the antimicrobial target (decreased drug affinity), decreased drug absorption, activation of efflux mechanisms to expel the harmful compound (overexpression efflux pumps), or global changes in critical metabolic pathways through the modulation of regulatory networks (71). One example is plant extracts that exhibit synergistic activity against microorganisms, as they present a complex mixture of natural products

and present a low risk in the development of bacterial resistance(72). Some natural products such as plant extracts, essential oils(73), and fixed oils(74) have modified the activity of antibiotics. Fig 6 shows the result of the MIC of buriti oil and OPG combined with norfloxacillin and gentamycin antibiotics.

Figure 6. MIC values (µg.mL^{-1}) of antibiotics in a bacterial growth assay in the presence of buriti oil (A) and buriti oil nanoencapsulated in porcine gelatin (OPG) (B). The tests were performed under the following conditions: norfloxacillin (white bar), norfloxacillin + crude buriti oil/OPG (light gray bar), gentamicin (dark gray bar), and gentamicin + crude buriti oil/OPG (black bar). ***p<0.0001.

Gentamycin is a drug used to treat infections, especially by Gram-negative bacteria, such as *E. coli* and *P. aeruginosa*, and it can also be used in combination(75). Its MIC for *E. coli* and *P. aeruginosa* is 1 µg.mL^{-1} and 2 µg.mL^{-1}, respectively(76,77). Norfloxacin is a broad-spectrum antibiotic used for Gram-positive and Gram-negative bacteria(78); it has a MIC of 25 µg.mL^{-1} for *E. coli*, 2 µg.mL^{-1} for *P. aeruginosa*, and 128 µg.mL^{-1} for *S. aureus*(79–81). Fixed buriti oil has a MIC >1024 for several strains, including *E. coli*, *P. aeruginosa*, and *S. aureus*(82).

Modulation with vegetable oils promotes better action on bacteria when there is a synergism of this combination, reducing the amount or increasing the medication used(82). The mechanisms of action between antibiotic and oil(83,84) may be related to the interaction with the lipid bilayer and cell membrane, affecting the respiratory chain and energy production by the bacteria(85), or even interference with bacterial enzymatic systems that may enhance the mechanism of action of antibiotics in combination with oils(86).

The combination of antibiotics with crude buriti oil (Fig 5A) and OPG (Fig 6B) showed greater efficiency in inhibiting the action of the *E. coli* bacteria when compared to isolated antibiotics. The combination of norfloxacillin and gentamycin antibiotics with the crude buriti oil reduced the MIC by 93.75% and 75%, respectively. Combining the same antibiotics with OPG reduced the MIC of norfloxacillin by 98% and gentamycin by 75% in *E. coli*. For gentamycin, the nanoencapsulated oil maintained the modulating activity of the crude oil in 75%.

Leão et al.(87) observed that a nanoemulsion containing buriti oil inhibited the microbial growth of *E. coli* by 61% at a concentration of 3.14 µg.mL^{-1}, demonstrating a bacteriostatic effect. With this, the researchers observed that the physicochemical characteristics of the nanoemulsion

contribute to its biological activity. The particle size can directly affect the antimicrobial activity of the emulsion based on essential oils, meaning a smaller size (nanometric scale) induces a greater inactivation of *E. coli* when compared to conventional emulsions(87).

Norfloxacillin alone, when compared with norfloxacillin combined with crude buriti oil (Fig 6A), did not present modulating activity for *S. aureus* with a statistically significant difference ($p>0.05$). However, the antibiotic activity combined with OPG decreased the MIC by 50% (Fig 6B). For gentamycin combined with crude oil such as OPG, there was an antagonism characterized by an increase in MIC by 100% in both demonstrated cases. Gentamycin does not act on Gram-positive bacteria, as *S. aureus*(75), showing that the nanoformulation maintained the crude buriti oil activity. Two main mechanisms are involved in antagonistic effects associated with natural products and antibiotics: chelation of antibiotic constituents by the natural product and competition of substances for the same binding site(88).

Norfloxacillin combined with buriti oil and OPG had greater inhibition for *P. aeruginosa* bacteria than norfloxacillin alone, with a statistical difference between them ($p<0.05$) and a 99% reduction in MIC with OPG and 97% with crude buriti oil. Gentamycin showed a significant difference in the two combinations (with free oil and OPG), in which there was a 50% reduction in MIC. In this case, both the free oil and OPG had a synergistic effect with the antibiotics.

Oils modify the antimicrobial effect depending on the antibiotic, oil tested and the bacterial species analyzed(14), which can be attributed to an interaction of the oil with the plasma membrane of the bacteria, increasing the permeability of the plasma membrane to the antibiotic(14,89,90).

Pereira et al.(82) used two antibiotics (gentamycin and amikacin) and two bacterial strains (*S. aureus* and *E. coli*) to verify the modulating activity of fixed buriti oil. The synergistic effect was observed in both antibiotics for *S. aureus*, with no MIC changes in *E. coli*.

The combination of buriti oil and OPG associated with norfloxacillin presenting FICi values ≤ 0.5 shows the synergistic effect for *E. coli* e *P. aeruginosa (Table 5).*

Table 5: Fractional inhibitory concentration (FIC) and fractional inhibitory concentration indices (FICi) of Buriti oil, OPG, norfloxacillin, and gentamycin.

**Microorganism**	**MIC** $_{\text{buriti oil}}$	**MIC** $_{\text{Norfloxacillin}}$	**MIC** $_{\text{Norfloxacillin+Buriti oil}}$	**FICi**

	($\mu g.mL^{-1}$)	($\mu g.mL^{-1}$)	($\mu g.mL^{-1}$)	
E. coli	64	128	8	0.2
P. aeruginosa	128	512	16	0.1
	**MIC $_{OPG}$ ($\mu g.mL^{-1}$)**	**MIC $_{Norfloxacillin}$ ($\mu g.mL^{-1}$)**	**MIC $_{Norfloxacillin+OPG}$ ($\mu g.mL^{-1}$)**	**FICi**
E. coli	16	128	2	0.1
P. aeruginosa	32	512	4	0.1

MIC: minimum inhibitory concentration, FIC: Fractional inhibitory concentration, FICi: fractional inhibitory concentration index, OPG: buriti oil nanoencapsulated in porcine gelatin.

Combinations of natural products and antibiotics(91) can affect more than one target for bacterial growth inhibition. This strategy is known as “herbal shotgun” or “synergistic multi-effect targeting,” in which different therapeutic components collaborate in a synergistic-agonistic way(88).

It is believed that both the antibacterial potential and the modulating activity of antibiotics attributed to vegetable oils are, at least in part, associated with the fatty acids present in the composition of the product since some fatty acids have already been shown to be able to improve the antimicrobial activity. And inhibit bacterial growth(15,92). It is reported that the potential of vegetable oils to act as antibacterial modulators is in part associated with the detergent property of fatty acids against the amphipathic structure of bacterial cell membranes (15). In this context, the synergistic effect observed against *E.coli* and *P. aeroginosa* may be associated with the detergent properties of fatty acids.

The literature points out that long-chain unsaturated fatty acids, such as oleic and palmitic, demonstrate antibacterial activity. In addition, the conjugated use of fatty acids and peptides or antibiotics can potentiate antimicrobial activity due to increased membrane permeability (93,94). This ability to solubilize membrane components (lipids and proteins) creates gaps in this structure that will affect metabolic processes essential for acquiring energy for the bacterial cell, such as the electron transport chain and oxidative phosphorylation. Membrane damage can also inhibit enzyme activity and toxic peroxidation (95). In addition, the presence of hydrophobic compounds in vegetable oils can increase the cell's permeability to antibiotics,

resulting in greater efficiency and reducing the minimum concentration necessary for the antibiotic to act against the bacteria (15,96).

The present study is innovative and unprecedented in the literature because it manages to evaluate the effect of the nanoformulation in the modulation of the antibiotic activity of known drugs, and not only of the non-encapsulated vegetable oil as presented in other studies. Thus, this study showed promising results for using free buriti oil and OPG in association with modifying antibiotic resistance against multiresistant strains such as *E. coli* and *P. aeroginosa*.

OPG is synthesized using low-cost materials. In addition, obtaining it is scalable, and drying by lyophilization is already used in the food industry. Thus, the nanoformulation has the potential to be incorporated into foods to add nutritional value due to the presence of buriti oil and its bioactive compounds, and also potential use in food packaging to act in the inhibition of pathogenic bacteria. Thus, OPG presents itself as a viable alternative to promote the preservation and/or enhancement of the oil's bioactive properties, which favors its use by the food and pharmaceutical industry, in addition to generating benefits to consumers' health. The application of nanotechnology in materials of plant origin promotes the valorization of this resource. It can contribute to the local development of the buriti-producing region within the perspective of circular economy and sustainable production.

References

1. Cattani IM, Baruque-Ramos J. Brazilian buriti palm fiber (Mauritia flexuosa Mart.). In Natural fibres: Advances in science and technology towards industrial applications. Springer, Dordrecht. 2016;89–98. Available from: https://doi.org/10.1007/978-94-017-7515-1

2. Cândido TLN, Silva MR, Agostini-Costa TS. Bioactive compounds and antioxidant capacity of buriti (Mauritia flexuosa L.f.) from the Cerrado and Amazon biomes. Food Chem [Internet]. 2015;177:313–9. Available from: http://dx.doi.org/10.1016/j.foodchem.2015.01.041

3. Cândido TLN, Silva MR. Comparison of the physicochemical profiles of buriti from the Brazilian Cerrado and the Amazon region. Food Sci Technol. 2017;37:78–82. Available from: https://doi.org/10.1590/1678-457X.32516

4. Silva SM, Sampaio KA, Taham T, Rocco SA, Ceriani R, Meirelles AJA. Characterization of oil extracted from buriti fruit (Mauritia flexuosa) grown in the Brazilian Amazon Region. JAOCS, J Am Oil Chem Soc. 2009;86(7):611–6. Available from:

https://doi.org/10.1007/s11746-009-1400-9

5. Ferreira BS, De Almeida CG, Faza LP, De Almeida A, Diniz CG, Da Silva VL, et al. Comparative properties of amazonian oils obtained by different extraction methods. Molecules. 2011;16(7):5874–85. Available from: https://doi.org/10.3390/molecules16075875

6. de Souza FG, de Araújo FF, de Paulo Farias D, Zanotto AW, Neri-Numa IA, Pastore GM. Brazilian fruits of Arecaceae family: An overview of some representatives with promising food, therapeutic and industrial applications. Food Res Int. 2020;138(September). Available from: https://doi.org/10.1016/j.foodres.2020.109690

7. Zanatta CF, Mitjans M, Urgatondo V, Rocha-Filho PA, Vinardell MP. Photoprotective potential of emulsions formulated with Buriti oil (Mauritia flexuosa) against UV irradiation on keratinocytes and fibroblasts cell lines. Food Chem Toxicol. 2010;48(1):70–5. Available from: https://doi.org/10.1016/j.fct.2009.09.017

8. Aquino JDS, Vasconcelos MHDA, Pessoa DCNDP, Soares JKB, Prado JPDS, Mascarenhas RDJ, et al. Intake of cookies made with buriti oil (: Mauritia flexuosa) improves vitamin A status and lipid profiles in young rats. Food Funct. 2016;7(10):4442–50. Available from: https://doi.org/10.1039/c6fo00770h

9. Hamacek FR, Della Lucia CM, Silva BP da, Moreira AVB, Pinheiro-Sant'ana HM. Buriti of the cerrado of Minas Gerais, Brazil: physical and chemical characterization and content of carotenoids and vitamins. Food Sci Technol. 2018;38(suppl 1):263–9. Available from: https://doi.org/10.1590/fst.15417

10. Galli F, Azzi A, Birringer M, Cook-Mills JM, Eggersdorfer M, Frank J, et al. Vitamin E: Emerging aspects and new directions. Free Radic Biol Med. 2017;102(June 2016):16–36. Available from: https://doi.org/10.1016/j.freeradbiomed.2016.09.017

11. Leslie MA, Cohen DJA, Liddle DM, Robinson LE, Ma DWL. A review of the effect of omega-3 polyunsaturated fatty acids on blood triacylglycerol levels in normolipidemic and borderline hyperlipidemic individuals. Lipids Health Dis. 2015;14(1). Available from: https://doi.org/10.1186/s12944-015-0049-7

12. Fuentes E, Rodríguez-Pérez W, Guzmán L, Alarcón M, Navarrete S, Forero-Doria O, et al. Mauritia flexuosa presents in vitro and in vivo antiplatelet and antithrombotic activities. Evidence-based Complement Altern Med. 2013;2013. Available from: https://doi.org/10.1155/2013/653257

13. Almeida MMB, de Sousa PHM, Arriaga ÂMC, do Prado GM, Magalhães CE de C, Maia GA, et al. Bioactive compounds and antioxidant activity of fresh exotic fruits from northeastern Brazil. Food Res Int. 2011 Aug 1;44(7):2155–9. Available from: https://doi.org/10.1016/J.FOODRES.2011.03.051

14. Oliveira RAG de, Lima EO, Vieira WL, Freire KRL, Trajano VN, Lima IO, et al. Estudo da interferência de óleos essenciais sobre a atividade de alguns antibióticos usados na clínica. Rev Bras Farmacogn. 2006;16(1):77–82. Available from: https://doi.org/10.1590/s0102-695x2006000100014

15. Saraiva RA, Matias EFF, Coutinho HDM, Costa JGM, Souza HHF, Fernandes CN, et al. Synergistic action between Caryocar coriaceum Wittm. fixed oil with aminoglycosides in vitro. Eur J Lipid Sci Technol. 2011;113(8):967–72. Available from: https://doi.org/10.1002/ejlt.201000555

16. Moser P, Ferreira S, Nicoletti VR. Buriti oil microencapsulation in chickpea protein-pectin matrix as affected by spray drying parameters. Food Bioprod Process [Internet]. 2019;117:183–93. Available from: https://doi.org/10.1016/j.fbp.2019.07.009

17. Moser P, Ferreira S, Nicoletti VR. Buriti oil microencapsulation in chickpea protein/pectin matrix as affected by spray drying parameters. Food Bioprod Process. 2019;34. Available from: https://doi.org/10.1016/j.fbp.2019.07.009

18. Santana AA, Kurozawa LE, de Oliveira RA, Park KJ. Influence of Process Conditions on the Physicochemical Properties of Pequi Powder Produced by Spray Drying. Dry Technol. 2013;31(7):825–36. Available from: https://doi.org/ 10.1080/07373937.2013.766619

19. Sharma S, Cheng S-F, Bhattacharya B, Chakkaravarthi S. Efficacy of free and encapsulated natural antioxidants in oxidative stability of edible oil: Special emphasis on nanoemulsion-based encapsulation. Trends Food Sci Technol [Internet]. 2019 Sep;91(July):305–18. Available from: https://doi.org/10.1016/j.tifs.2019.07.030

20. Castro GMMA, Passos TS, Nascimento SS da C, Medeiros I, Araújo NK, Maciel BLL, et al. Gelatin nanoparticles enable water dispersibility and potentialize the antimicrobial activity of Buriti (Mauritia flexuosa) oil. BMC Biotechnol. 2020;20(1):1–13. Available from: https://doi.org/10.1186/s12896-020-00649-4

21. Kaushik P, Dowling K, Barrow CJ, Adhikari B. Microencapsulation of omega-3 fatty acids: A review of microencapsulation and characterization methods. J Funct Foods. 2015;19:868–81. Available from: https://doi.org/10.1016/j.jff.2014.06.029

22. Lira KHD da S, Passos TS, Ramalho HMM, Rodrigues D da SR, Vieira É de A, Cordeiro AMT de, et al. Whey protein isolate-gelatin nanoparticles enable the water-dispersibility and potentialize the antioxidant activity of quinoa oil (Chenopodium quinoa). PLoS One. 2020;30:1–17. Available from: https://doi.org/10.1371/journal.pone.0240889

23. Faridi Esfanjani A, Jafari SM. Biopolymer nano-particles and natural nano-carriers for nano-encapsulation of phenolic compounds. Colloids Surfaces B Biointerfaces. 2016 Oct 1;146:532–43. Available from: https://doi.org/10.1016/J.COLSURFB.2016.06.053

24. Medeiros AK de OC, Gomes C de C, Amaral MLQ de A, Medeiros LDG de, Medeiros I, Porto DL, et al. Nanoencapsulation improved water solubility and color stability of carotenoids extracted from Cantaloupe melon (Cucumis melo L.) [Internet]. Vol. 270, Food Chemistry. Elsevier; 2019 [cited 2019 Oct 1]. 562–572 p. Available from: https://doi.org/10.1016/J.FOODCHEM.2018.07.099

25. El-Messery TM, Altuntas U, Altin G, Özçelik B. The effect of spray-drying and freeze-drying on encapsulation efficiency, in vitro bioaccessibility and oxidative stability of krill oil nanoemulsion system. Food Hydrocoll. 2020;106(March). Available from: https://doi.org/10.1016/j.foodhyd.2020.105890

26. Prieto P, Pineda M, Aguilar M. Spectrophotometric quantitation of antioxidant capacity through the formation of a …. Anal Biochem [Internet]. 1999;269:337–41. Available from: Available from: https://doi.org/ 10.1037/a0037168

27. Wang J, Zhang Q, Zhang Z, Li Z. Antioxidant activity of sulfated polysaccharide fractions extracted from Laminaria japonica. Int J Biol Macromol [Internet]. 2008 Mar 1 [cited 2019 Feb 27];42(2):127–32. Available from: https://doi.org/10.1016/J.IJBIOMAC.2007.10.003

28. Rufino M do S, Alves RE, Brito ES De, Morais SM De, Sampaio CDG, Saura-calixto FD. Metodologia Científica: Determinação da atividade antioxidante total em frutas pela captura do radical livre ABTS. Comunicado Técnico 128. 2007. p. 0–3. Available from: ISSN 1679-6535

29. Harb TB, Torres PB, Pires JS, Santos DYAC, Chow F. Ensaio em microplaca do potencial antioxidante através do sistema quelante de metais para extratos de algas. Inst Biociências da Univ São Paulo. 2016;(April 2018):2–6. Available from: https://doi.org/10.13140/RG.2.2.35838.69446

30. de Oliveira GLR, Medeiros I, Nascimento SS da C, Viana RLS, Porto DL, Rocha HAO, et al. Antioxidant stability enhancement of carotenoid rich-extract from Cantaloupe melon (Cucumis melo L.) nanoencapsulated in gelatin under different storage conditions. Food Chem. 2021;348(September 2020). Available from: https://doi.org/10.1016/j.foodchem.2021.129055

31. Bezerra CF, Camilo CJ, do Nascimento Silva MK, de Freitas TS, Ribeiro-Filho J, Coutinho HDM. Vanillin selectively modulates the action of antibiotics against resistant bacteria. Microb Pathog [Internet]. 2017;113(September):265–8. Available from: https://doi.org/10.1016/j.micpath.2017.10.052

32. Patel J.B., Cockerill R.F., Bradford A.P., Eliopoulos M.G., Hindler A.J., Jenkins G.S., Lewis S.J., Limbago B., Miller A.L., Nicolau P.D., Pwell M., Swenson M.J., Traczewski M.M., Turnidge J.D. WPMZLB. M07-A10: Methods for Dilution Antimicrobial Susceptibility Tests for Bacteria That Grow Aerobically; Approved Standard—Tenth Edition. CLSI (Clinical Lab Stand Institute). 2015;35(2). Available from:

https://doi.org/10.1007/s00259-009-1334-3

33. Rocha RR, Matos MNC, Guerrero JAP, Cavalcante RMB, Melo RS, Azevedo ÁMA, et al. Comparative study of the chemical composition, antibacterial activity and synergic effects of the essential oils of Croton tetradenius baill. And C. pulegiodorus baill. Against Staphylococcus aureus isolates. Microb Pathog. 2021;156(January). Available from: https://doi.org/10.1016/j.micpath.2021.104934

34. Chen L, Remondetto GE, Subirade M. Food protein-based materials as nutraceutical delivery systems. Trends Food Sci Technol. 2006;17(5):272–83.

35. Kumar A, Dixit CK. Methods for characterization of nanoparticles. In: Advances in Nanomedicine for the Delivery of Therapeutic Nucleic Acids. 2017. p. 44–58. Available from: https://doi.org/10.1016/B978-0-08-100557-6.00003-1

36. Danaei M, Dehghankhold M, Ataei S, Hasanzadeh Davarani F, Javanmard R, Dokhani A, et al. Impact of particle size and polydispersity index on the clinical applications of lipidic nanocarrier systems. Pharmaceutics. 2018;10(2):1–17. Available from: https://doi.org/10.3390/pharmaceutics10020057

37. Silverstein RM, Webster FX, Kiemle DJ. Silverstein-Spectrometric Identification of Organic Compounds 7th ed. 7th ed. New York: John Wiley & Sons; 2005. 550 p. Available from: ISBN 1118311655, 9781118311653

38. Guerrero P, Stefani PMM, Ruseckaite RAA, de la Caba K. Functional properties of films based on soy protein isolate and gelatin processed by compression molding. J Food Eng [Internet]. 2011 Jul 1 [cited 2019 Oct 14];105(1):65–72. Available from: https://doi.org/10.1016/j.jfoodeng.2011.02.003

39. Esfahani R, Jafari SM, Jafarpour A, Dehnad D. Loading of fish oil into nanocarriers prepared through gelatin-gum Arabic complexation. Food Hydrocoll [Internet]. 2019;90(December 2018):291–8. Available from: https://doi.org/10.1016/j.foodhyd.2018.12.044

40. Batista JS, Olinda RG, Medeiros VB, Rodrigues CMF, Oliveira AF, Paiva ES, et al. Atividade antibacteriana e cicatrizante do óleo de buriti Mauritia flexuosa L. Ciência Rural. 2012;42(1):136–41. Available from: https://doi.org/10.1590/S0103-84782012000100022

41. Sagiri SS, Anis A, Pal K. Review on Encapsulation of Vegetable Oils: Strategies, Preparation Methods, and Applications. Polym - Plast Technol Eng. 2016;55(3):291–311. Available from: https://doi.org/10.1080/03602559.2015.1050521

42. Can Karaca A, Low NH, Nickerson MT. Potential use of plant proteins in the microencapsulation of lipophilic materials in foods. Trends Food Sci Technol. 2015;42(1):5–12. Available from: https://doi.org/10.1016/j.tifs.2014.11.002

43. da Silva BC, de Oliveira M, Ferreira JGL, Sierakowski MR, Simas-Tosin FF, Orth ES, et al. Polyelectrolyte complexes from gum arabic and gelatin: Optimal complexation pH as a key parameter to obtain reproducible microcapsules. Food Hydrocoll. 2015;46:201–7. Available from: https://doi.org/10.1016/j.foodhyd.2014.12.022

44. Wu Y, Guo J, Yang W, Wang C, Fu S. Preparation and characterization of chitosan-poly(acrylic acid) polymer magnetic microspheres. Polymer (Guildf). 2006;47(15):5287–94. Available from: https://doi.org/10.1016/j.polymer.2006.05.017

45. Zhao Y, Sun Z. Effects of gelatin-polyphenol and gelatin–genipin cross-linking on the structure of gelatin hydrogels. Int J Food Prop. 2018;20(3):S2822–32. Available from: https://doi.org/10.1080/10942912.2017.1381111

46. Durães JA, Drummond AL, Pimentel TAPF, Murta MM, Moreira SGC, Sales MJA. Thermal and structural behavior of Buriti oil/poly(methyl methacrylate) and Buriti oil/polystyrene materials. J Therm Anal Calorim. 2008;92(2):529–34. Available from: https://doi.org/10.1007/s10973-007-8564-z

47. Jayadas NH, Nair KP. Coconut oil as base oil for industrial lubricants-evaluation and modification of thermal, oxidative and low temperature properties. Tribol Int. 2006;39(9):873–8. Available from: https://doi.org/10.1016/j.triboint.2005.06.006

48. Klein T, Longhini R, Bruschi ML, De Mello JCP. Microparticles containing Guaraná extract obtained by spray-drying technique: Development and characterization. Rev Bras Farmacogn. 2015;25(3):292–300. Available from: https://doi.org/10.1016/j.bjp.2015.03.010

49. Liz GR de, Verruck S, Canella MHM, Dantas A, Garcia SG, Maran BM, et al. Stability of bifidobacteria entrapped in goat’s whey freeze concentrate and inulin as wall materials and powder properties. Food Res Int. 2020;127(February 2019):10. Available from: https://doi.org/10.1016/j.foodres.2019.108752

50. Escobar-García JD, Prieto C, Pardo-Figuerez M, Lagaron JM. Room temperature nanoencapsulation of bioactive eicosapentaenoic acid rich oil within whey protein microparticles. Nanomaterials. 2021;11(3):1–16. Available from: https://doi.org/10.3390/nano11030575

51. Garcia CC, Franco PIBM, Zuppa TO, Filho NRA, Leles MIG. Thermal stability studies of some cerrado plant oils. J Therm Anal Calorim. 2007;87(3):645–8. Available from: https://doi.org/10.1007/s10973-006-7769-x

52. Vecchio S, Cerretani L, Bendini A, Chiavaro E. Thermal decomposition study of monovarietal extra virgin olive oil by simultaneous thermogravimetry/differential scanning calorimetry: Relation with chemical composition. J Agric Food Chem. 2009;57(11):4793–800. Available from: https://doi.org/10.1021/jf900120v

53. Nde DB, Anuanwen CF. Optimization methods for the extraction of vegetable oils: A review. Processes. 2020;8(2):21. Available from: https://doi.org/10.3390/pr8020209

54. Pereira Lima R, Souza da Luz PT, Braga M, dos Santos Batista PR, Ferreira da Costa CE, Zamian JR, et al. Murumuru (Astrocaryum murumuru Mart.) butter and oils of buriti (Mauritia flexuosa Mart.) and pracaxi (Pentaclethra macroloba (Willd.) Kuntze) can be used for biodiesel production: Physico-chemical properties and thermal and kinetic studies. Ind Crops Prod [Internet]. 2017;97:536–44. Available from: http://doi.org/10.1016/j.indcrop.2016.12.052

55. Schappo FB. Nanopartículas de óleo de palma bruto: estabilidade e citotoxicidade. Programa de Pós-Gaduação em Ciência dos Alimentos. Universidade Federala de Santa Catarina; 2021.

56. International Organization for Standardization. ISO 10993-5: Tests for in vitro cytotoxicity. In: Biological evaluation of medical devices. 2009. p. 42.

57. Zanatta CF, Ugartondo V, Mitjans M, Rocha-Filho PA, Vinardell MP. Low cytotoxicity of creams and lotions formulated with Buriti oil (Mauritia flexuosa) assessed by the neutral red release test. Food Chem Toxicol. 2008;46(8):2776–81. Available from: http://doi.org/10.1016/j.fct.2008.05.001

58. Melo-Silveira RF, Fidelis GP, Pereira Costa MSS, Telles CBS, Dantas-Santos N, de Oliveira Elias S, et al. In vitro antioxidant, anticoagulant and antimicrobial activity and in inhibition of cancer cell proliferation by xylan extracted from corn cobs. Int J Mol Sci. 2012;13(1):409–26. Available from: http://doi.org/10.3390/ijms13010409

59. Ribeiro PPCPPC, E Silva DMDLDMDL, De Assis CFCF, Correia RTPRTP, Damasceno KSFDSCKSFDSC. Bioactive properties of faveleira (Cnidoscolus quercifolius) seeds, oil and press cake obtained during oilseed processing. PLoS One. 2017;12(8):1–12. Available from: http://doi.org/10.1371/journal.pone.0183935

60. Silva DMLE, Damasceno KSFSC, Ribeiro PPC, Alcântra MA, Cordeiro AMTM, Passos TS, et al. Chemical characteristics and antioxidant activity of astaxanthin extracted from shrimp residues using soybean oil. J Braz Chem Soc. 2021;32(6):1277–85. Available from: http://doi.org/10.1371/journal.pone.0183935

61. Zhong Y, Shahidi F. Antioxidant behavior in bulk oil: Limitations of polar paradox theory. J Agric Food Chem. 2012;60(1):4–6. Available from: http://doi.org/10.1021/jf204165g

62. Melo-Silveira RF, Fidelis GP, Viana RLS, Soeiro VC, Da Silva RA, Machado D, et al. Antioxidant and antiproliferative activities of methanolic extract from a neglected agricultural product: Corn cobs. Molecules. 2014;19(4):5360–78. Available from: http://doi.org/10.3390/molecules19045360

63. Mau JL, Lai EYC, Wang NP, Chen CC, Chang CH, Chyau CC. Composition and

antioxidant activity of the essential oil from Curcuma zedoaria. Food Chem. 2003;82(4):583–91. Available from: http://doi.org/10.1016/S0308-8146(03)00014-1

64. Dos Santos PDF, Francisco CRL, Coqueiro A, Leimann FV, Pinela J, Calhelha RC, et al. The nanoencapsulation of curcuminoids extracted from: Curcuma longa L. and an evaluation of their cytotoxic, enzymatic, antioxidant and anti-inflammatory activities. Food Funct. 2019;10(2):573–82. Available from: http://doi.org/10.1039/c8fo02431f

65. Huang X, Liu Y, Zou Y, Liang X, Peng Y, McClements DJ, et al. Encapsulation of resveratrol in zein/pectin core-shell nanoparticles: Stability, bioaccessibility, and antioxidant capacity after simulated gastrointestinal digestion. Food Hydrocoll. 2019;93(December 2018):261–9. Available from: http://doi.org/10.1016/j.foodhyd.2019.02.039

66. Luo Y. Food colloids binary and ternary nanocomplexes: Innovations and discoveries. Colloids Surfaces B Biointerfaces. 2020;196(August):111309. Available from: http://doi.org/10.1016/j.colsurfb.2020.111309

67. Nagaraju PG, Sindhu P, Dubey T, Chinnathambi S, Poornima Priyadarshini CG, Rao PJ. Influence of sodium caseinate, maltodextrin, pectin and their Maillard conjugate on the stability, in vitro release, anti-oxidant property and cell viability of eugenol-olive oil nanoemulsions. Int J Biol Macromol. 2021;183:158–70. Available from: http://doi.org/10.1016/j.ijbiomac.2021.04.122

68. de Campo C, dos Santos PP, Costa TMH, Paese K, Guterres SS, Rios A de O, et al. Nanoencapsulation of chia seed oil with chia mucilage (Salvia hispanica L.) as wall material: Characterization and stability evaluation. Food Chem [Internet]. 2017;234:1–9. Available from: http://dx.doi.org/10.1016/j.foodchem.2017.04.153

69. Bilenler T, Gokbulut I, Sislioglu K, Karabulut I. Antioxidant and antimicrobial properties of thyme essential oil encapsulated in zein particles. Flavour Fragr J. 2015;30(5):392–8. Available from: http://doi.org/10.1002/ffj.3254

70. Coutinho HDM, Costa JGM, Falcão-Silva VS, Siqueira-Júnior JP, Lima EO. Effect of Momordica charantia L. in the resistance to aminoglycosides in methicilin-resistant Staphylococcus aureus. Comp Immunol Microbiol Infect Dis. 2010;33(6):467–71. Available from: http://doi.org/10.1016/j.cimid.2009.08.001

71. de Medeiros VM, do Nascimento YM, Souto AL, Madeiro SAL, Costa VC de O, Silva SMPM, et al. Chemical composition and modulation of bacterial drug resistance of the essential oil from leaves of Croton grewioides. Microb Pathog. 2017 Oct 1;111:468–71. Available from: http://doi.org/10.1016/J.MICPATH.2017.09.034

72. Daferera DJ, Ziogas BN, Polissiou MG. The effectiveness of plant essential oils on the growth of Botrytis cinerea, Fusarium sp. and Clavibacter michiganensis subsp.

michiganensis. Crop Prot. 2003;22(1):39–44. http://doi.org/10.1016/S0261-2194(02)00095-9

73. Coutinho HDM, Costa JGM, Lima EO, Falcão-Silva VS, Siqueira JP. Herbal therapy associated with antibiotic therapy: Potentiation of the antibiotic activity against methicillin - Resistant Staphylococcus aureus by Turnera ulmifolia L. BMC Complement Altern Med. 2009;9:1–4. Available from: http://doi.org/10.1186/1472-6882-9-13

74. Al Ashaal HA, Farghaly AA, Abd El Aziz MM, Ali MA. Phytochemical investigation and medicinal evaluation of fixed oil of Balanites aegyptiaca fruits (Balantiaceae). J Ethnopharmacol. 2010;127(2):495–501. Available from: http://doi.org/10.1016/j.jep.2009.10.007

75. Chen C, Chen Y, Wu P, Chen B. Update on new medicinal applications of gentamicin: Evidence-based review. J Formos Med Assoc. 2014;113(2):72–82. Available from: http://doi.org/10.1016/j.jfma.2013.10.002

76. Garrod LP, Waterworth PM. Effect of medium composition on the apparent sensitivity of Pseudomonas aeruginosa to gentamicin. J Clin Pathol. 1969;22(5):534–8. Available from: http://doi.org/10.1136/jcp.22.5.534

77. Medeiros AA, O'Brien TF, Wacker WEC, Yulug NF. Effect of salt concentration on the apparent in-vitro susceptibility of pseudomonas and other gram-negative bacilli to gentamicin. J Infect Dis. 1971;124(December):S59–64. Available from: http://doi.org/10.1080/10408347.2014.941456

78. Chierentin L, Salgado HRN. Review of Properties and Analytical Methods for the Determination of Norfloxacin. Crit Rev Anal Chem. 2016;46(1):22–39. Available from: http://doi.org/10.1080/10408347.2014.941456

79. Aoyama H, Sato K, Kato T, Hirai K, Mitsuhashi S. Norfloxacin resistance in a clinical isolate of Escherichia coli. Antimicrob Agents Chemother. 1987;31(10):1640–1. Available from: http://doi.org/10.1128/AAC.31.10.1640

80. King A, Warren C, Shannon K, Phillips I. In vitro antibacterial activity of norfloxacin (MK-0366). Antimicrob Agents Chemother. 1982;21(4):604–7. Available from: http://doi.org/10.1128/AAC.21.4.604

81. Diniz-Silva HT, Magnani M, de Siqueira S, de Souza EL, de Siqueira-Júnior JP. Fruit flavonoids as modulators of norfloxacin resistance in Staphylococcus aureus that overexpresses norA. LWT - Food Sci Technol. 2017;85:324–6. Available from: http://doi.org/10.1016/j.lwt.2016.04.003

82. Pereira YF, Costa MDS, Tintino SR, Rocha JE, Rodrigues FFG, Feitosa MK de SB, et al. Modulation of the antibiotic activity by the Mauritia flexuosa (Buriti) fixed oil against methicillin-resistant staphylococcus aureus (MRSA) and other multidrug-resistant (MDR)

bacterial strains. Pathogens. 2018;7(4):1–8. Available from: http://doi.org/10.3390/pathogens7040098

83. Coutinho HDM, Costa JGM, Siqueira-Júnior JP, Lima EO. In vitro anti-staphylococcal activity of Hyptis martiusii Benth against methicillin-resistant Staphylococcus aureus-MRSA strains. Rev Bras Farmacogn. 2008;18(SUPPL.):670–5. Available from: http://doi.org/10.1590/S0102-695X2008000500005

84. Coutinho HDM, Costa JGM, Lima EO, Falcão-Silva VS, Siqueira JP. Enhancement of the antibiotic activity against a multiresistant Escherichia coli by Mentha arvensis L. and chlorpromazine. Chemotherapy. 2008;54(4):328–30. Available from: http://doi.org/10.1159/000151267

85. Nicolson K, Evans G, O'Toole PW. Potentiation of methicillin activity against methicillin-resistant Staphylococcus aureus by diterpenes. FEMS Microbiol Lett. 1999;179(2):233–9. Available from: http://doi.org/10.1016/S0378-1097(99)00417-6

86. Wendakoon CN, Morihiko S. Inhibition of amino acid decarboxylase activity of enterobacter aerogenes by active components in spices. Vol. 58, Journal of Food Protection. 1995. p. 280–3. Available from: http://doi.org/10.4315/0362-028X-58.3.280

87. Leão KMM, Reis LVC, Speranza P, Rodrigues AP, Ribeiro APB, Macedo JA, et al. Physicochemical characterization and antimicrobial activity in novel systems containing buriti oil and structured lipids nanoemulsions. Biotechnol Reports. 2019;24. Available from: http://doi.org/10.1016/j.btre.2019.e00365

88. Wagner H, Ulrich-Merzenich G. Synergy research: Approaching a new generation of phytopharmaceuticals. Phytomedicine. 2009;16(2–3):97–110. Available from: http://doi.org/ 10.1016/j.phymed.2008.12.018

89. Medeiros Barreto H, Cerqueira Fontinele F, Pereira De Oliveira A, Arcanjo DDR, Cavalcanti Dos Santos BH, De Abreu APL, et al. Phytochemical prospection and modulation of antibiotic activity in vitro by lippia origanoides H.B.K. in methicillin resistant staphylococcus aureus. Biomed Res Int. 2014;2014. Available from: http://doi.org/10.1155/2014/305610

90. Sousa EO, Barreto FS. Atividade antibacteriana e interferência de Lantana camara L. e Lantana montevidensis (Spreng.) Briq. na resistência de aminoglicosídeos. Rev Bras Biociências. 2011;9(1):1–5. Available from: ISSN 1980-4849

91. Matias EEF, Santos KKA, Almeida TS, Costa JGM, Coutinho HDM. Enhancement of Antibiotic Activity by Cordia verbenacea DC. Lat Am J Pharm. 2010;29:1049–52.

92. Lee JY, Kim YS, Shin DH. Antimicrobial synergistic effect of linolenic acid and monoglyceride against Bacillus cereus and Staphylococcus aureus. J Agric Food Chem. 2002;50(7):2193–9. Available from: http://doi.org/10.1021/jf011175a

93. Chu-Kung AF, Bozzelli KN, Lockwood NA, Haseman JR, Mayo KH, Tirrell M V. Promotion of peptide antimicrobial activity by fatty acid conjugation. Bioconjug Chem. 2004;15(3):530–5. Available from: http://doi.org/10.1021/bc0341573

94. Bera S, Zhanel GG, Schweizer F. Design, synthesis, and antibacterial activities of neomycin-lipid conjugates: Polycationic lipids with potent gram-positive activity. J Med Chem. 2008;51(19):6160–4. Available from: http://doi.org/10.1021/jm800345u

95. Desbois AP, Smith VJ. Antibacterial free fatty acids: activities, mechanisms of action and biotechnological potential. Appl Microbiol Biotechnol [Internet]. 2010 Jan 15;85:1629+. Available from: https://link.gale.com/apps/doc/A229606459/AONE?u=capes&sid=bookmark-AONE&xid=82eb837a

96. Sousa EO de, Rodrigues FFG, Campos AR, Costa JGM da. <b>Phytochemical analysis and modulation in aminoglycosides antibiotics activity by *Lantana camara* L. Acta Sci Biol Sci [Internet]. 2015 Aug 6;37(2):213. Available from: http://periodicos.uem.br/ojs/index.php/ActaSciBiolSci/article/view/22877

Universidade Federal do Rio Grande do Norte
Departamento de Farmácia

Natal, February 21th, 2022

Dear Editor-in-Chief at the PLos One,

Thank you for considering our manuscript entitled ***"Nanoencapsulation of buriti oil in porcine gelatin enhances the antioxidant potential and improves the effect on the antibiotic activity modulation"*** for revision. We have carefully read the reviewers' comments, answered all questions in this letter, and made the requested changes in the revised manuscript (highlighted in yellow). We hope the revised manuscript is now suitable for publication in PLos One.

Best regards,

Cristiane Fernandes de Assis
Department of Pharmacy
Federal University of Rio Grande do Norte
Phone: (55 84) 99152-7007
E-mail: cristianeassis@hotmail.com

Answers to reviewer #1:

The authors are carried out research for nanoformulation of buriti oil by using porcin gelatin in a scientific way, however, the need to describe the criteria was fixed in the selection of buriti fruits and formulation or production process is not well described. In the introduction part, the research gap is not clear?

Thank you for the comment. The suggestions were accepted. We made the proper changes in the results text, highlighted in yellow. The buriti oil used in the study was kindly donated by a company (Plantus® S/A) with an IBD certificate, which markets the product in Brazil and exports it to other countries. Regarding the nanoparticle synthesis process, the protocol followed was the same as that standardized

by Castro et al. (1) (publication of our research group), aiming at obtaining a new batch of OPG with the same physical and chemical characteristics observed by Castro et al.(1) to continue the investigations related to the effect of nanoencapsulation on the functionality of buriti oil.

The results and discussion part have a good delivery to the information but lacking in scientific support studies. This study was already carried out by Castro et al. and Garcia et al. in terms of Characterization OPG and what is your innovation or what would you tell to the scientific community or food industrialist?

Thank you for the comment. Our research group has been developing studies with nanoencapsulated vegetable oils (1,2). The present study is a continuation of the study of Castro et al. (1) that investigated the nanoencapsulation of buriti oil using a different encapsulating agent [porcine gelatin (OPG) and the combination of sodium alginate and porcine gelatin (OAG)], to evaluate the effect on water dispersibility and antimicrobial activity. The investigation pointed to best results for OPG, the smallest particle size [51.0 (6.07) nm], chemical interaction between the materials present in the system, greater dispersibility in water [85.62% (7.82)]. It potentiated the antimicrobial activity of buriti oil in 59%, 62%, and 43% against the bacteria Pseudomonas aeruginosa, Klebsiella pneumonia, and Staphylococcus aureus, respectively.

Therefore, it is of fundamental importance to deepen the knowledge regarding the effects of nanoencapsulation on the biological properties of buriti oil to assess the antioxidant potential and modulating action on antibiotics of nanoparticles based on porcine gelatin containing buriti oil (OPG). Thus, it was necessary to ensure that the new batch of synthesized nanoformulation had the same physical and chemical characteristics observed in Castro. et al.(1) Therefore, it was necessary to carry out the characterization to confirm that the diameter, morphology, chemical interactions and incorporation efficiency were similar to the batch obtained by Castro et al. (1), ensuring that the material's functionality would be preserved and possible to continue the study. This step is of fundamental importance to guarantee the standardization of each batch of nanoformulation obtained. The present study is innovative and unprecedented in the literature because it manages to evaluate the effect of the nanoformulation in the modulation of the antibiotic activity of known drugs, and not only of the non-encapsulated vegetable oil as presented in other studies. Interest in products developed through Nanotechnology has increased not only in the food industry but also in the pharmaceutical industry due to properties such as the high dissolution rate associated with the increased permeability of the active compound through the intestinal wall, increased physical and chemical stability of the

core, the ease of handling, transport, and incorporation into the matrix of a product. In addition, to enabling water solubility, bioaccessibility and bioavailability of lipophilic substances, preservation and enhancement of functionalities, and enabling the reduction of the concentration of the bioactive of interest necessary to achieve the desired biological effect.

Units are not well described as per author guidelines. Grammatical mistakes appeared in the manuscript and it was corrected the manuscript. Mandatory for reframing the conclusion part in the first 4 lines, the present form of conclusion is not opt for the manuscript. Figure quality is not good and mandatory need to improve the quality in all figures. Corrections were mentioned in the manuscript and need to carry out. Check the references and some of the references were missing in DOI?
Thank you for the comment. The suggestions were accepted. We made the proper changes in the results text, highlighted in yellow. We have carefully addressed the manuscript's grammar, usage, and overall readability. This English review was carried out by a native English speaker with experience in international research. We also used the software Grammarly (full version) to further check for gross mistakes. We are fully available to respond to any further questions, attending to other suggestions. Besides, the units and conclusions were adjusted based on the author's guidelines. The reference list was revised for the insertion of the DOI, and the resolution of the figures was improved.

Answers to reviewer #2:

The findings of the investigation are interesting, it uses suitable experiments to show the characteristic of the nanoencpasulated material and its action for antibiotic activity modulation. However there are some weakness in the manuscript that need to be observed. I suggest to accept the manuscript after major revision. My comments are given below.
*Thank you for considering our manuscript "**Nanoencapsulation of buriti oil in porcine gelatin enhances the antioxidant potential and improves the effect on the antibiotic activity modulation**" for revision.*

1. How encapsulation facilitates the delivery of buriti oil. Author should perform the release experiment for its practical application.
The present study is a continuation of a study previously published by our research group (Castro et al.,(1), which proposed to enable the nanoencapsulation of buriti oil, aiming to obtain a formulation

with a nanoscale diameter, distribution of homogeneous size, capable of allowing the dispersibility of the oil in water, and potentiating the antimicrobial activity, that is, increasing the action using a smaller amount of buriti oil. Thus, Castro et al.(1) tested different materials (sodium alginate, porcine gelatin, the combination of both) and obtained a formulation (OPG – nanoencapsulated buriti oil in porcine gelatin) that achieved the expected results. Based on this, the main focus of the present study was to deepen the investigation of the effect of nanoencapsulation on the buriti oil functionalities, such as the antioxidant activity and modulation of the antimicrobial activity of known antibiotics and the impact on thermal stability. In addition, it was of fundamental importance to evaluate the toxicity of OPG in cells, aiming at future studies in animal models. Therefore, future studies by our research group will determine in vitro simulated digestion to obtain information on controlled release, antioxidant and anti-inflammatory effects, complementing the information with studies in eutrophic animal models and diet-induced obesity.

2. Why nanoencapsulation increased the antioxidant activity of OPG

Nanoscale diameter allows for increased contact surface for chemical interactions, increased water dispersibility, bioaccessibility, and bioavailability compared to micro or macro scale size (3). Therefore, nanoencapsulated oils are protected against oxidation of their bioactive constituents and have a higher solubility in water (1,2,4). These associated factors guarantee an increase in the antioxidant potential.

3. Why author selected porcine gelatin as wall polymer, state the prominent reason. There are other biocompatible polymers also.

Porcine gelatin is a low-cost material, widely used in the food, cosmetic and pharmaceutical industry, as a coating, binding, gelling agent in confectionery, capsules and food supplements, creams, lotions, among others, due to its structural stability, and physicochemical, functional and nutritional properties. It can act as an emulsifier in oil-in-water emulsions, promoting an increase in the physical-chemical stability of polyunsaturated fats. In addition, the gelling property is of great interest for applications in the health area, as it helps in the adhesion of the particle to the mucosa to promote the controlled release.

4. Authors demonstrated herbal shotgun or synergistic multi-effect targeting in this investigation. This is very interesting finding. But, how author confirmed that the efficacy was due to synergism. In

support of this statement statistical model showing the synergistic data is required.

Thank you for the comment. The suggestions were accepted. We made the proper changes in the results text, highlighted in yellow. We inserted in the article the methodology and the result (Table 5) in which we demonstrate the synergistic effect of buriti oil / OPG and antibiotics.

5. SEM micrograph is not very clear.

Thank you for the comment. The resolution of the micrographs was improved using a tool indicated by PLos One.

6. Author found the combination of antibiotics with crude buriti oil and OPG showed greater efficiency in inhibiting the action of the *E. coli* bacteria when compared to isolated antibiotics. Why this enhancement in efficacy was found. Give the prominent reason.

According to published studies, the antibacterial potential and modulating activity of antibiotics attributed to oils are, at least in part, associated with the fatty acids present in their composition since some fatty acids have already been shown to be able to improve antibiotic activity and inhibit the growth of bacteria (5,6). Studies suggest that oils' potential to act as antibacterial or antibiotic modulators may be associated with the detergent property of fatty acids against the amphipathic structure of bacterial cell membranes (7). This detergent property can act on the membrane (lipids and proteins), creating pores in the structure that will affect the metabolic processes of bacteria that are essential for their survival (8). Furthermore, the hydrophobic compounds in oils can increase the cell's permeability to the antibiotic, increasing the efficiency and decreasing the MIC of the antibiotic (5,9). It should be noted that these studies and possible mechanisms of action were included in the discussion of the present study, and the changes were highlighted in yellow.

7. Before adding commercial value to buriti oil, investigation about cost effectiveness is an important parameter to be considered. Author must incorporate the cost effectivity for encapsulation and how it can be commercially used?

Thank you for the comment. The suggestions were accepted. We made the proper changes in the results text, highlighted in yellow. The nanoformulation is synthesized using low-cost materials. In addition, obtaining it is scalable, and drying by lyophilization is already used in the food industry. Thus, the

nanoformulation has the potential to be incorporated into foods to add nutritional value due to the presence of buriti oil and its bioactive compounds, and also potential use in food packaging to act in the inhibition of pathogenic bacteria.

8. Author presented this in line 142-145 as “After the encapsulation process, the resulting emulsion was subjected to drying by lyophilization (LioTop L101) at -57ºC and pressure of 43 μHg for further characterization, evaluation of cytotoxicity, antioxidant potential, and effect on the modulation of antibiotic activity”. Why author performed the experiments of cytotoxicity, antioxidant potential, and antibiotic modulatory activity by lyophilized sample? What is the difference, if the experiment was performed through prepared emulsion? I think, after lyophilization, there is a change of aggregation and agglomeration of particles, therefore, chances of experimental hindrance about the results.

Based on the literature (10,11) one of the advantages of the emulsification technique is to use the emulsions obtained in fluid or solid form. Therefore, powder forms can be obtained by drying the fluid emulsion by spray-drying or lyophilization. Thus, the drying of emulsions expands the range of applications in food, packaging, cosmetics, and pharmaceuticals, as it facilitates the dispersion of the material in an aqueous matrix. Based on this, the emulsion obtained was dried to expand its use in several products and not just to carry out the investigations presented in this study.

9. Why Tween 20 was used as surfactant in this investigation? Why not Tween-80, based on HLB values it is more used for nanoencapsulation.

Based on published studies, such as the one by Yuan et al. (12) , from the group of polysorbates, Tween 20 stands out as the surfactant with the highest hydrophilic-lipophilic balance (HLB), approximately 16.7, being possible to promote the stabilization of the interface between oil and water, allowing to obtain particles of smaller size compared to other polysorbates.

REFERENCES

1. Castro GMMA, Passos TS, Nascimento SS da C, Medeiros I, Araújo NK, Maciel BLL, et al. Gelatin nanoparticles enable water dispersibility and potentialize the antimicrobial activity of Buriti (Mauritia flexuosa) oil. BMC Biotechnol. 2020;20(1):1–13.
2. Lira KHD da S, Passos TS, Ramalho HMM, Rodrigues D da SR, Vieira É de A, Cordeiro

AMT de, et al. Whey protein isolate-gelatin nanoparticles enable the water-dispersibility and potentialize the antioxidant activity of quinoa oil (Chenopodium quinoa). PLoS One. 2020;30:1–17.

3. Tabibiazar M, Davaran S, Hashemi M, Homayonirad A, Rasoulzadeh F, Hamishehkar H, et al. Design and fabrication of a food-grade albumin-stabilized nanoemulsion. Food Hydrocoll [Internet]. 2015;44:220–8. Available from: http://dx.doi.org/10.1016/j.foodhyd.2014.09.005

4. de Campo C, dos Santos PP, Costa TMH, Paese K, Guterres SS, Rios A de O, et al. Nanoencapsulation of chia seed oil with chia mucilage (Salvia hispanica L.) as wall material: Characterization and stability evaluation. Food Chem [Internet]. 2017;234:1–9. Available from: http://dx.doi.org/10.1016/j.foodchem.2017.04.153

5. Saraiva RA, Matias EFF, Coutinho HDM, Costa JGM, Souza HHF, Fernandes CN, et al. Synergistic action between Caryocar coriaceum Wittm. fixed oil with aminoglycosides in vitro. Eur J Lipid Sci Technol. 2011;113(8):967–72.

6. Pereira YF, Costa MDS, Tintino SR, Rocha JE, Rodrigues FFG, Feitosa MK de SB, et al. Modulation of the antibiotic activity by the Mauritia flexuosa (Buriti) fixed oil against methicillin-resistant staphylococcus aureus (MRSA) and other multidrug-resistant (MDR) bacterial strains. Pathogens. 2018;7(4):1–8.

7. Chu-Kung AF, Bozzelli KN, Lockwood NA, Haseman JR, Mayo KH, Tirrell M V. Promotion of peptide antimicrobial activity by fatty acid conjugation. Bioconjug Chem. 2004;15(3):530–5.

8. Desbois AP, Smith VJ. Antibacterial free fatty acids: activities, mechanisms of action and biotechnological potential. Appl Microbiol Biotechnol [Internet]. 2010 Jan 15;85:1629+. Available from: https://link.gale.com/apps/doc/A229606459/AONE?u=capes&sid=bookmark-AONE&xid=82eb837a

9. Sousa EO de, Rodrigues FFG, Campos AR, Costa JGM da. <b>Phytochemical analysis and modulation in aminoglycosides antibiotics activity by *Lantana camara* L. Acta Sci Biol Sci [Internet]. 2015 Aug 6;37(2):213. Available from: http://periodicos.uem.br/ojs/index.php/ActaSciBiolSci/article/view/22877

10. Ferreira CD, Nunes IL. Oil nanoencapsulation: development, application, and incorporation into the food market. Nanoscale Res Lett. 2019;14.

11. Comunian TA, Silva MP, Moraes ICF, Favaro-Trindade CS. Reducing carotenoid loss during storage by co-encapsulation of pequi and buriti oils in oil-in-water emulsions followed by freeze-drying: Use of heated and unheated whey protein isolates as emulsifiers. Food Res Int

[Internet]. 2020;130:108901. Available from: http://www.sciencedirect.com/science/article/pii/S0963996919307872

12. Yuan Y, Gao Y, Zhao J, Mao L. Characterization and stability evaluation of β-carotene nanoemulsions prepared by high pressure homogenization under various emulsifying conditions. Food Res Int [Internet]. 2008;41(1):61–8. Available from: http://www.sciencedirect.com/science/article/pii/S0963996907001627
